# High-throughput continuous evolution of compact Cas9 variants targeting single-nucleotide-pyrimidine PAMs

**DOI:** 10.1038/s41587-022-01410-2

**Published:** 2022-09-08

**Authors:** Tony P. Huang, Zachary J. Heins, Shannon M. Miller, Brandon G. Wong, Pallavi A. Balivada, Tina Wang, Ahmad S. Khalil, David R. Liu

**Affiliations:** 1 Merkin Institute of Transformative Technologies in Healthcare, The Broad Institute of Harvard and MIT, Cambridge, MA USA; 2 Department of Chemistry and Chemical Biology, Harvard University, Cambridge, MA USA; 3 Howard Hughes Medical Institute, Harvard University, Cambridge, MA USA; 4 Biological Design Center, Boston University, Boston, MA USA; 5 Department of Biomedical Engineering, Boston University, Boston, MA USA; 6 Department of Chemistry, University of Wisconsin-Madison, Madison, WI USA; 7 Wyss Institute for Biologically Inspired Engineering, Harvard University, Boston, MA USA

**Keywords:** Genetic engineering, Biotechnology

## Abstract

Despite the availability of Cas9 variants with varied protospacer-adjacent motif (PAM) compatibilities, some genomic loci—especially those with pyrimidine-rich PAM sequences—remain inaccessible by high-activity Cas9 proteins. Moreover, broadening PAM sequence compatibility through engineering can increase off-target activity. With directed evolution, we generated four Cas9 variants that together enable targeting of most pyrimidine-rich PAM sequences in the human genome. Using phage-assisted noncontinuous evolution and eVOLVER-supported phage-assisted continuous evolution, we evolved Nme2Cas9, a compact Cas9 variant, into variants that recognize single-nucleotide pyrimidine-PAM sequences. We developed a general selection strategy that requires functional editing with fully specified target protospacers and PAMs. We applied this selection to evolve high-activity variants eNme2-T.1, eNme2-T.2, eNme2-C and eNme2-C.NR. Variants eNme2-T.1 and eNme2-T.2 offer access to N_4_TN PAM sequences with comparable editing efficiencies as existing variants, while eNme2-C and eNme2-C.NR offer less restrictive PAM requirements, comparable or higher activity in a variety of human cell types and lower off-target activity at N_4_CN PAM sequences.

## Abstract (web summary)

Directed evolution creates new compact Cas9 variants that target most single pyrimidine nucleotide PAMs.

## Main

CRISPR–Cas9 has enabled the development of genome-manipulating technologies that have transformed the life sciences and advanced new treatments for genetic disorders into the clinic^1,2^. Target sites engaged by Cas9 must contain a protospacer-adjacent motif (PAM) that is recognized through a protein–DNA interaction before single-guide RNA (sgRNA) binding^1^. While not prohibitive for some gene editing applications, such as target gene disruption, this PAM requirement limits the applicability of precision gene editing methods, including base editing, prime editing or site-specific DNA integration^3,4^. For these technologies, the target modification must occur either at a specific distance or within a certain range of the PAM^3^. Thus, the availability of a PAM sequence compatible with a Cas protein that retains robust activity in mammalian cells strongly determines the application scope of precision gene editing. Indeed, recent ex vivo and in vivo therapeutic base editing to rescue sickle-cell disease^5^ and progeria^6^ in mice used evolved or engineered Cas9 variants to precisely position the base editor at CACC or NGA PAMs (where N is A, C, G or T), respectively.

The limitations imposed by PAM restrictions have motivated efforts to engineer or evolve Cas protein variants with broadened or altered PAM compatibility. These approaches have generated variants of the most widely used Cas9 from *Streptococcus pyogenes* (SpCas9)^7–11^, which offers robust mammalian cell activity and engages sites with NGG PAMs^1^. The wild-type and evolved or engineered variants of SpCas9 described so far can collectively access essentially all purine-containing PAMs and a subset of pyrimidine-containing PAMs^7–11^.

Researchers have also parsed the genomes of other bacterial species or bacteriophage to identify Cas variants with different PAM requirements^3,12^. These Cas variants vary dramatically in size, PAM compatibility and enzymatic activity^3,4,13^. Unfortunately, most of these natural homologs are less well characterized, less active in mammalian cells or have highly restrictive PAM requirements compared to SpCas9 (ref. ^13^), limiting their use for precision gene editing applications and the ease with which they can be modified. As such, engineering or evolution of non-SpCas9 orthologs has been uncommon, with only a few reported examples^14–16^.

New engineering or evolution methods to address the limitations of reprogramming non-SpCas9 orthologs could provide new precision gene editing capabilities that expand on and complement the suite of commonly used SpCas9-derived variants. Nme2Cas9, a Cas9 variant from *Neisseria meningitidis*, is an attractive Cas ortholog for evolving PAM compatibility^17^. The wild-type enzyme is active on N_4_CC PAMs, and thus may serve as a promising starting point to all pyrimidine PAMs previously inaccessible by SpCas9 variants. In addition, Nme2Cas9 has a smaller size than SpCas9 (1,082 versus 1,368 amino acids (aa)), making it attractive for future delivery applications. Nme2Cas9 has also shown robust activity in mammalian cells as both a nuclease and a base editor^17,18^.

Here we report the directed evolution of Nme2Cas9 (ref. ^17^), expanding its PAM scope from the N_4_CC requirement of the wild-type protein to include most N_4_YN sequences, where Y is C or T. To enable the evolution of this non-SpCas9 ortholog, we developed and integrated three technologies. First, we established a new, generalizable selection strategy requiring both PAM recognition and functional editing activity. We carried out selections in parallel across single PAM sequences using phage-assisted non-continuous evolution (PANCE)^19^ and a high-throughput eVOLVER-enabled^20^ phage-assisted continuous evolution (ePACE) platform. Last, we developed a high-throughput base editing-dependent PAM-profiling assay (BE-PPA) to rapidly and thoroughly characterize evolving Nme2Cas9 variants and to guide evolutionary trajectories. With these developments, we evolved four Nme2Cas9 variants that enable robust precision genome editing at PAMs with a single specified pyrimidine nucleotide: eNme2-C, eNme2-C.NR, eNme2-T.1 and eNme2-T.2. The evolved Nme2 variants exhibit comparable (eNme2-T.1 and eNme2-T.2) or more robust (eNme2-C) base editing and lower off-target editing than SpRY, the only other engineered variant capable of accessing similar PAMs for a subset of target sites^7^. Together, these new variants offer broad PAM accessibility that is complementary to the suite of PAMs previously targetable by SpCas9-derived variants. Moreover, the selection strategy developed in this study is highly scalable and general. Because of the lack of target site requirements, this selection could in principle be applied to evolve functional activities in any Cas ortholog or to optimize editing at a specific PAM or target site.

## Results

We hypothesized that our continuous evolution system, PACE^21^, in which the propagation of M13 bacteriophage is coupled to the desired activity of a protein of interest, could be used to evolve Nme2Cas9 variants with expanded pyrimidine-rich PAM scope. Previously, we broadened the PAM scope of SpCas9 variants using a one-hybrid, DNA-binding PACE circuit^8,9^. In those efforts, SpCas9 variants encoded on selection phage (SP) capable of simply binding the target PAM(s) successfully produce gene III (*gIII*), a gene essential for phage propagation. The resulting SpCas9 variants could access most NR PAM sequences (where R is A or G), but efforts to apply the DNA-binding selection to evolve pyrimidine-PAM recognition were less successful^8,9^.

**Fig. 1:**
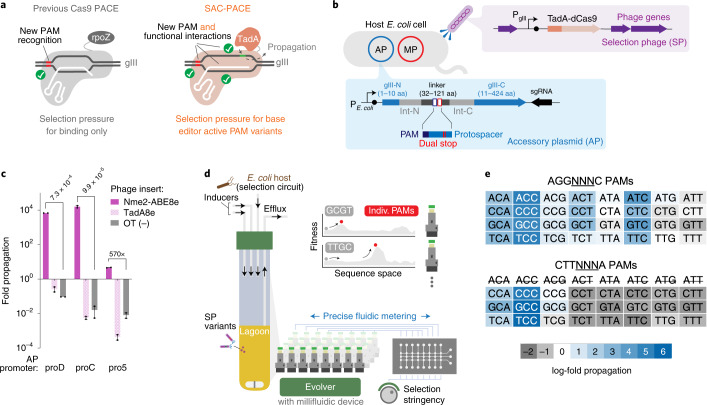
Development of a function-dependent Cas9 selection and the ePACE platform for automated parallel evolution. **a**, Overview of previous Cas9 PACE (left) requiring only PAM binding, compared to the SAC-PACE developed in this study, which requires both PAM binding and subsequent base editing. **b**, The selection circuit in SAC-PACE. The SP encodes an adenine base editor in place of *gIII*. In the host cells, an accessory plasmid (AP) contains a *cis* intein-split *gIII*, with a linker (31–121 aa) containing stop codons. MP, mutagenesis plasmid 6 (ref. ^39^). **c**, Overnight phage propagation assays to test the selection stringency of SAC-PACE with various AP promoter strengths. Mean ± s.e.m. are shown and are representative of *n* = 2 independent biological replicates. **d**, Overview of ePACE, enabling parallel lagoon evolution of a Cas9 variant on single PAMs (Supplementary Figs. 1–4). **e**, Overnight propagation assays of wild-type Nme2-ABE8e on two sets of 32 N_3_NYN PAMs. Fold-propagation was measured by qPCR and is reflective of the average of two independent biological replicates. The eight CTTAYNA PAMs are excluded as they introduce an additional stop codon in the accessory plasmid, preventing Cas-dependent propagation.

While this binding selection could be adapted to evolve Nme2Cas9, fundamental differences between the activities of SpCas9 and Nme2Cas9 could impede efforts to evolve the PAM scope of the latter. Nme2Cas9, and more broadly Type II-C Cas variants, may have slower nuclease kinetics relative to SpCas9 (ref. ^13^). This weaker nuclease activity is attributed to slower Cas9 helicase activity, as artificially introduced bulges mimicking partially unwound DNA in the PAM-proximal region increase the cleavage rate of Type II-C Cas variants but not of SpCas9 (ref. ^13^). This theory is supported by observations that miniaturized SpCas9 variants with partially deleted domains have reduced DNA-binding affinity that can also be rescued by the introduction of PAM-proximal bulges in target DNA^22^. Because a primary motivation for broadening PAM compatibility is to improve the applicability of precision gene editing technologies that require DNA unwinding^3^, it is critical that a selection preserves or improves R-loop formation, maintenance and nuclease activation. Notably, these Cas properties are dependent on domains outside the PAM-interacting domain (PID), which has been the focus of rational engineering approaches^7,10,14,15^. Together, this analysis indicates that while DNA-binding selections or PID engineering can yield robust SpCas9 variants with altered PAM compatibilities, the same type of binding-only selection applied to the evolution of Nme2Cas9 or similar Cas orthologs may not yield both desired PAM recognition and efficient downstream activity (Fig. 1a). This hypothesis motivated us to envision a new, functional selection in PACE for evolving PAM compatibility.

### Development of a general functional selection for evolving PAM compatibility in PACE

To develop a functional selection for Cas9-based genome editing agents with altered PAM compatibilities, we combined elements of a DNA-binding selection^8,9^ with a base editing (BE) selection^23,24^, such that both new PAM recognition and subsequent BE within the protospacer are required to pass the selection. Although we previously developed BE selections to evolve high-activity adenine and cytidine deaminases^23,24^, these selections place targeted nucleotides within the coding sequence of T7 RNA polymerase (T7 RNAP). This selection strategy is not broadly applicable to evolve altered PAM compatibility since changing the target PAM and protospacer likely requires changing the coding sequence of T7 RNAP. Furthermore, evolved variants with high activity that edit over large activity windows may inadvertently alter the activity of T7 RNAP through bystander editing.

To address these limitations, we designed a new selection strategy in which the target protospacer and PAM can be fully specified without affecting the coding sequence of the gene responsible for selection survival (Fig. 1b). To achieve this programmability, we used the splicing capabilities of inteins, protein elements that insert and remove themselves from other proteins in *cis*, leaving only a small (roughly 3- to 10-aa) extein scar^25,26^. We hypothesized that *trans* split-inteins could function effectively as *cis*-splicing elements when the N- and C-inteins are fused together with a linker containing a programmed PAM and protospacer. We used the split-intein pair from *N. punciforme* (Npu)^27^ since we previously showed that *gIII* split after Leu 10 with the Npu intein supports robust phage propagation after *trans* splicing^28^.

**Extended Data Fig. 1:**
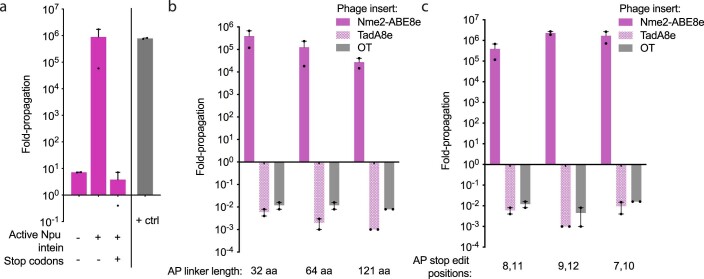
Validation of the sequence-agnostic Cas (SAC-PACE) PACE selection. **(a)** Overnight propagation assay to test the requirements of active intein splicing and stop codons to turn on or off, respectively, the SAC-PACE circuit. Inactive intein was generated by introducing the C1A mutation^41^ in the C-intein and the positive control (+ctrl) was a host strain containing pJC175e^19^. **(b)** Overnight propagation assay to test the linker length limitations of SAC-PACE, OT phage did not contain Nme2-ABE8e or TadA8e. **(c)** Overnight propagation assay to test the relative activity of Nme2-ABE8e phage when the target adenines within the stop codons are placed at different locations in the 23 nucleotide Nme2Cas9 protospacer (counting the PAM as positions 24-29). For (**a-c**), Mean±SEM is shown and are representative of *n* = *2* independent biological replicates. Fold-propagation is calculated as the ratio of titer after overnight propagation over inoculating titer.

To test whether the reconfigured *cis*-splicing Npu intein supports phage propagation, we constructed an accessory plasmid with the N- and C-terminal halves of the Npu intein fused together with a flexible 32-aa linker and inserted into the coding sequence of *gIII* after Leu 10 under the control of the phage shock promoter (psp)^29^ (Fig. 1b). When infected with Δ*gIII*-phage, host cells containing this accessory plasmid supported robust phage propagation in a splicing-dependent manner similar to cells containing psp-driven wild-type *gIII*. Installation of stop codons within the linker sequence reduced phage propagation by >10^5^-fold relative to the unmutated construct (Extended Data Fig. 1a), indicating that this selection, which we term sequence-agnostic Cas-PACE (SAC-PACE), should enable robust selection of variants capable of correcting targeted stop codons.

Next, we tested whether adenine base editing could support phage propagation in SAC-PACE. Indeed, on host cells harboring an accessory plasmid containing *gIII* with two stop codons flanked by a cognate Nme2Cas9 N_4_CC PAM, phage encoding dead Nme2Cas9 fused to the adenosine deaminase TadA8e (ref. ^23^) (Nme2-ABE8e) enriched 10^2^- to 10^6^-fold after overnight propagation, depending on the expression level of the *gIII*-construct (Fig. 1c). In contrast, phage containing only TadA8e or a nontargeting gene de-enriched in these host cells below the limit of detection at any tested expression level, indicating a large base editing-dependent dynamic range for this selection.

To test the generality of the selection circuit, we generated a series of APs containing linkers between 32 and 121 aa or with stop codons placed at different positions within the protospacer (Extended Data Fig. 1b,c). Although propagation decreased with increasing linker length, the maximum tested linker length of 121 aa still supported strong overnight propagation sufficient to support phage survival during PACE (>10^4^-fold)^19^. This linker length can encode up to ten simultaneous protospacer/PAM combinations (23 to 30 nucleotides (nt) in length) with at least 7 nt between targets, a spacing shown to be compatible for multiple Cas protein binding events^30^. Together, these results indicate that the SAC-PACE selection is a highly flexible system that could be used to evolve the PAM scope of Cas variants.

### A high-throughput platform for ePACE

Previous efforts to evolve SpCas9 on specific PAM sequences (NAG, NAC, NAT, etc.) yielded variants with both higher activity and specificity compared to variants evolved on a broad set of pooled PAMs^9^. Evolving on specific PAM sequences using traditional PACE methodology, however, is limited by throughput, since PACE is inherently challenging to parallelize due to cost, space and design complexity, requiring temperature-controlled rooms and fluid-handling equipment^31^. This constraint limits the number of conditions that can be explored in a PACE campaign, a drawback given the difficulty of predicting the set of conditions that will evolve molecules with desired properties.

To address this throughput challenge and enable large-scale parallel PACE of Nme2Cas9 toward specific PAMs, we developed ePACE (Fig. 1d and Supplementary Figs. 1–3). The ePACE system combines the continuous mutagenesis and selection of PACE with the highly scalable, customizable and automated eVOLVER continuous culture platform, which has already proved effective for directed evolution^32^. Three key design features of eVOLVER make it an ideal choice for facilitating parallel PACE selections. First, eVOLVER enables individual programmatic control of continuous culture conditions, allowing the platform to simultaneously operate PACE chemostat cell reservoirs and lagoons on a standard laboratory benchtop. Second, eVOLVER can scale in a cost-effective manner to arbitrary throughput, enabling large-scale parallelization of miniature PACE reactors. Last, the do-it-yourself and open-source nature of eVOLVER allow it to be rapidly adapted and reconfigured for new actuation elements, making it amenable to the customization necessary to run PACE (Supplementary Figs. 1–3). Integrating PACE and eVOLVER enables the simultaneous execution of PACE experiments across eight different PAMs (or other selection conditions) in parallel. Given that PACE experiments typically require 1–2 weeks each, this eightfold increase in throughput represents a 2–4-month reduction in experimental time compared to traditional single-lagoon PACE at a tenfold reduction in cost.

To facilitate and automate the liquid handling needs of PACE in eVOLVER, we developed customized ‘millifluidic’ integrated peristaltic pumps (IPPs), inspired by integrated microfluidics^33^, that can be inexpensively manufactured using laser cutting to achieve accurate, tunable small volume flow rates (<0.1–40 µl s^−1^) (Supplementary Figs. 2 and 3 and Supplementary Note 1). Briefly, IPPs enable accurate and tunable metering of liquids through the sequential actuation of consecutively arranged pneumatic valves. We characterized several IPP valve sizes and cycle frequencies to generate calibration curves of achievable flow rates and verified robustness of these pumps over roughly 6 million actuations over 7 days, well over the typical load necessary for PACE (Supplementary Figs. 2 and 3). To test the evolutionary capabilities of ePACE, we evolved a folding-defective (G32D/I33S) maltose-binding protein (MBP) variant validated in traditional PACE^28^. Previously, this folding-defective MBP was evolved using a two-hybrid selection scheme to optimize both soluble expression of the MBP variant and binding to an anti-MBP monobody^28^. We replicated this evolution using ePACE, yielding evolved MBP variants with mutations at residues clustered around the monobody-MBP interaction interface (D32G, A63T, R66L) that we previously observed in PACE (Supplementary Fig. 4)^28^. These results demonstrate that eVOLVER equipped with IPP devices can successfully support and automate PACE, validating the ePACE platform for high-throughput continuous directed evolution.

### Development of a high-throughput base editing-dependent PAM-profiling method

Next, we developed a method to rapidly profile the PAM scope of Nme2Cas9 variants that emerge during evolution. Assessing PAM compatibility by testing individual sites in mammalian cells is throughput-limited. Although many library-based PAM-profiling methods have been described, these methods rely on nuclease activity (PAM depletion^10^, PAMDA^7,15^, TXTL PAM profiling^34^, CHAMP^35^, etc.) or Cas protein binding activity (PAM-SCANR^36^, CHAMP^35^, etc.), which may not fully reflect PAM compatibility in precision gene editing applications such as base editing. We previously reported a mammalian cell base editing profiling assay^9,37^; however, this method is both slower and costlier than cell-free^34,35^ or *E. coli-*based^7,10,15,36^ methods, making it better suited for the characterization of late-stage variants.

**Extended Data Fig. 2:**
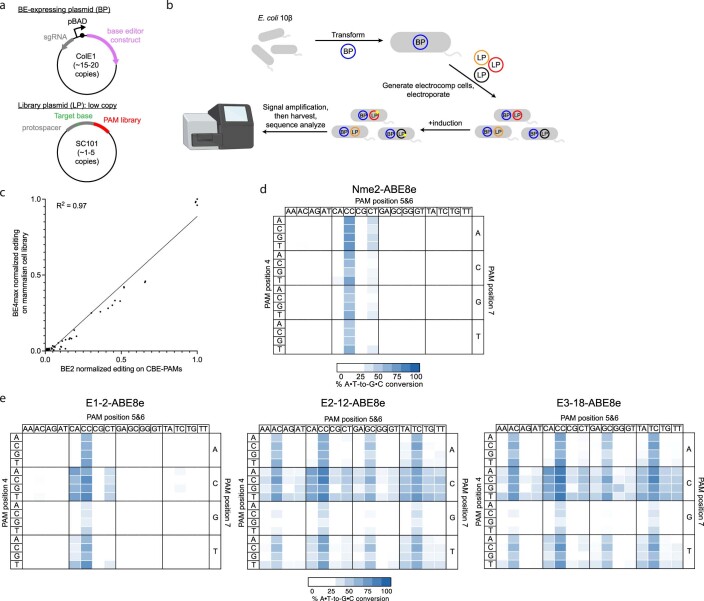
Base editing dependent PAM profiling assay (BE- PPA). **(a)** Schematic of BE-PPA constructs. A BE-expressing plasmid (BP) containing the base editor to be evaluated is cloned along with a library plasmid (LP) containing a target protospacer and target base (adenine or cytosine for ABE-PPA or CBE-PPA, respectively) flanked by a library of PAMs of interest. **(b)** BE- PPA workflow. A cell line containing the BP is first generated, then the LP is electroporated into that cell line before base editor expression is induced. Induced cells are grown for 22–36 hours (with dilution after 24 hours if necessary), before plasmid DNA is harvested and sequenced by high-throughput sequencing. **(c)** Comparison of the BE-PPA assay against existing mammalian cell base editing PAM profiling^9^. Each point represents 1 of 64 NNN PAMs, normalized to the activity of the highest PAM for BE2 (rAPOBEC1-dSpCas) along the x- axis in BE-PPA or for BE4max along the y-axis for the previously assessed mammalian library. All points reflect the average normalized activity of *n* = 2 independent biological replicates. The line reflects a simple ordinary least squares (OLS) regression, with the R-squared value shown. **(d, e)** Heat maps showing ABE-PPA activity of (d) wild-type Nme2-ABE8e and (e) representative clones from ePACE1-3 on the set of 256 N_3_NNNN PAMs (PAM positions 1-3 fixed, see Supplementary Table 1). Values are raw % A•T-to-G•C conversion observed for one replicate of each base editor.

To address the need to rapidly assess the PAM specificities of newly evolved Cas9 variants in base editor form, we developed a base editing-dependent PAM-profiling assay (BE-PPA). In BE-PPA, a protospacer or library of protospacers containing target adenines (ABE-PPA) or cytosines (CBE-PPA) is installed upstream of a library of PAM sequences (Extended Data Fig. 2a,b). This library is transformed into *E. coli* along with a plasmid expressing a base editor of interest. Since base editing at each PAM is measured independently of other PAMs, BE-PPA offers greater sensitivity compared to nuclease-based assays. The PAM profile we observed for BE2 (rAPOBEC1-dSpCas9-UGI) using CBE-PPA closely matched (*R*^2^ = 0.97) the PAM profile we previously observed for the related CBE, BE4, in mammalian human embryonic kidney 293T (HEK293T) cells^9^ (Extended Data Fig. 2c and Supplementary Table 1), validating BE-PPA as a rapid base editor PAM-profiling method.

### Strategy for evolving the PAM scope of Nme2Cas9

**Fig. 2:**
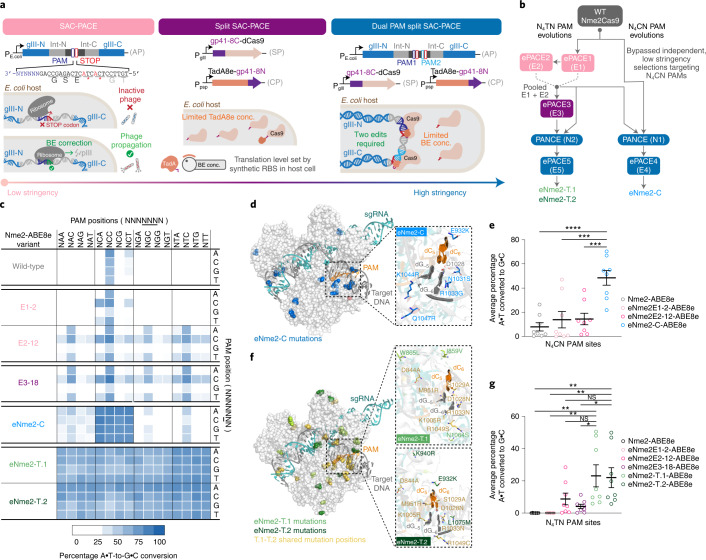
Evolution of Nme2Cas9 variants with broadened PAM activity. **a**, SAC-PACE modifications increasing selection stringency. Left, original selection scheme; middle, split SAC-PACE selection and right, dual-PAM split SAC-PACE. **b**, Evolution campaigns toward Nme2Cas9 variants with N_4_CN or N_4_TN PAM compatibility. **c**, Summary heat map showing ABE-PPA activity for representative variants across both evolutionary trajectories. Values plotted are raw observed percentage A•T-to-G•C conversion for one replicate of each base editor. **d**, Mutation overview of the eNme2-C variant, mapped onto the crystal structure of wild-type Nme2Cas9 (Protein Data Bank (PDB) 6JE3), mutated positions are shown in blue. The inset shows the wild-type PAM and PAM-interacting residues (D1028, R1033), with evolved mutations listed. **e**, Summary dot plots showing the progression of mammalian cell adenine base editing activity at eight N_4_CN PAM-containing sites for representative variants from the N_4_CN evolution trajectory. **f**, Mutation overview of the eNme2-T.1 and eNme2-T.2 variants, mapped onto the crystal structure of wild-type Nme2Cas9 (PDB 6JE3). Positions mutated in both variants are shown in yellow. Mutations unique to eNme2-T.1 are shown in light green. Mutations unique to eNme2-T.2 are shown in dark green. The insets show the wild-type PAM and PAM-interacting residues (D1028, R1033), along with new mutations listed. **g**, Summary dot plots showing the progression of mammalian cell adenine base editing activity at eight N_4_TN PAM-containing sites for representative variants from the N_4_TN evolution trajectory. For **e** and **g**, each point represents the average editing of *n* = 3 independent biological replicates measured at the maximally edited position within each genomic site. Mean ± s.e.m. is shown and reflects the average activity and standard error of the pooled genomic site averages. NS, *P* > 0.05; *P ≤ 0.05; **P ≤ 0.01, ***P ≤ 0.001, ****P ≤ 0.0001. *P* values determined by Sidak’s multiple comparisons test following ordinary one-way analysis of variance. For **e**, eNme2-C-ABE8e versus Nme2-ABE8e (*P* < 0.0001), eNme2E1-2-ABE8e (*P* = 0.0003), eNme2E2-12-ABE8e (*P* = 0.0004) and for **g** eNme2-T.1-ABE8e versus Nme2-ABE8e (*P* = 0.0020), eNme2E1-2-ABE8e (*P* = 0.0020), eNme2E2-12-ABE8e (*P* = 0.1252), eNme2E3-18-ABE8e (*P* = 0.0157) or eNme2-T.2-ABE8e versus Nme2-ABE8e (*P* = 0.0037), eNme2E1-2-ABE8e (*P* = 0.0037), eNme2E2-12-ABE8e (*P* = 0.1947) and eNme2E3-18-ABE8e (*P* = 0.0272).

Having validated the SAC-PACE selection, the ePACE system for high-throughput continuous evolution and the BE-PPA method for profiling PAM compatibility of base editors, we next identified desirable target PAMs for evolving Nme2Cas9. In overnight propagation assays, phage containing Nme2-ABE8e exhibited modest to strong propagation (N_3_NCG < N_3_NCA < N_3_NCT < N_3_NCC) on the set of 16 N_3_NCN PAMs, and strong propagation on N_3_NTC PAMs if the base immediately downstream of the canonical six base pair PAM was a C (PAM position 7, NNNNNNN, counting the canonical PAM as positions 1–6), likely due to PAM slippage (Fig. 1e)^38^. This initial activity suggested an overall evolution campaign along two trajectories (Fig. 2b): a more difficult trajectory toward activity on N_4_TN PAMs that could require several selection stringencies, and a simpler trajectory toward N_4_CN-active variants. If successful, these variants could together enable targeting of PAM sequences largely complementary to the PAM scope of existing, high-activity SpCas9 variants.

### Low-stringency evolution of Nme2Cas9 toward N_4_TN PAM sequences

We first used our evolution platform to perform parallel SAC-PACE selections to evolve Nme2Cas9 variants toward specific N_4_TN PAM sequences (Fig. 2). We envisioned using the initial activity of wild-type Nme2Cas9 on some N_4_TC PAMs (Fig. 1d) as an evolutionary stepping-stone to access other N_4_TN PAMs. Using the original (low-stringency) SAC-PACE selection featuring one protospacer, two stop codons and one target PAM (Fig. 2a, left panel), we evolved wild-type Nme2-ABE8e on host cells containing APs with each of the eight possible N_3_YTN APs and the mutagenesis plasmid (MP6)^39^ (ePACE1, Fig. 2b). As expected, all APs aside from those containing a N_3_TTC or N_3_CTC PAM washed out rapidly. However, those two PAM-containing lagoons persisted at up to 2 volumes per hour and yielded Nme2Cas9 variants with PAM-dependent mutational convergence (Supplementary Figs. 5 and 6a). Consensus mutations occurred both inside (I1025S, R1033K, S1043R for CTC PAM variants, Y1035C/H for TTC PAM variants) and outside the PID (Y441C, K581R, D844V/G for CTC PAM variants; I462V, N616S, D844V for TTC PAM variants), suggesting potential PAM-specific and PAM-independent improvements to Nme2Cas9. Indeed, early evolved variants (for example, E1-2-ABE8e) supported base editing activity on noncanonical PAMs and improved activity on wild-type N_4_CC PAMs in human cells (Supplementary Fig. 6b). Expanded PAM activity appeared strongest on N_4_CN PAMs and was minimal on N_4_TN PAMs.

We reseeded all PAM lagoons with pooled phage from the two surviving PAMs (ePACE2) (Fig. 2b). All lagoons now exhibited strong propagation at up to 2.5 volumes per hour (Supplementary Fig. 7), but surviving phage appeared to lose the Nme2-ABE8e cassette, indicating recombination to bypass the selection (Supplementary Figs. 8a–c and 9 and Supplementary Note 2). We sequenced clones that did not show recombination and found mutations that again appeared to cluster by PAM/lagoon both in and outside the PID (Supplementary Fig. 10a). In mammalian cells, while expanded PAM compatibility did extend to some N_4_TN PAMs, activity appeared to be site-dependent while moderate activity on N_4_CN PAMs was retained (Supplementary Fig. 10b). These ePACE1 and ePACE2 outcomes suggested that the low-stringency SAC-PACE selection may be insufficient to generate highly active Nme2Cas9 PAM variants.

We used ABE-PPA to profile the PAM compatibility of wild-type Nme2-ABE8e and a representative ABE variant from both ePACE1 (E1-2-ABE8e) and ePACE2 (E2-12-ABE8e) that had exhibited improved mammalian cell base editing activity on N_4_YN PAMs (Fig. 2c, Extended Data Fig. 2d,e and Supplementary Table 1). While both evolved variants exhibited improved activity on N_4_CD (where D is A, G or T) PAMs over Nme2-ABE8e (17, 23 and 32% average A•T-to-G•C conversion for Nme2-ABE8e, E1-2-ABE8e and E2-12-ABE8e, respectively), only the more evolved variant, E2-12-ABE8e, exhibited improved N_4_TN PAM activity (2, 2 and 39% average A•T-to-G•C conversion for Nme2-ABE8e, E1-2-ABE8e and E2-12-ABE8e, respectively). This result indicates a model in which broadened activity on N_4_CN PAMs precedes activity on N_4_TN PAMs.

Further examination of the ABE-PPA data indicated that broadened PAM activity of early evolved Nme2Cas9 variants was primarily driven by an acquired C preference at the undesired PAM position 7, a position not recognized by the wild-type enzyme^40^. While E1-2-ABE8e and E2-12-ABE8e progressively improve base editing activity compared to wild-type Nme2-ABE8e on N_4_YNC PAM sites (18, 29 and 58% average A•T-to-G•C conversion for Nme2-ABE8e, E1-2-ABE8e and E2-12-ABE8e, respectively), base editing activity was improved to a lesser extent at N_4_YND PAM sites (14, 14 and 33% average A•T-to-G•C conversion for Nme2WT ABE8e, E1-2-ABE8e and E2-12-ABE8e, respectively). This discrepancy suggested the need for higher selection stringency to restrict the survival of Cas variants that acquire expanded PAM recognition at undesired positions.

### Increasing SAC-PACE selection stringency to evolve high-activity Nme2Cas9 variants

In previous efforts evolving SpCas9, restricting the amount of active enzyme and requiring additional PAM recognition via a multi-PAM system increased selection stringency and enabled evolution of higher activity variants^9^. We hypothesized similar strategies could be implemented in SAC-PACE to evolve high-activity Nme2Cas9 variants while preventing selectivity at undesired PAM positions (Fig. 2a). To limit the amount of active base editor, we used a split-intein strategy with the base editor split at the linker between TadA8e and dNme2Cas9, which we hypothesized could tolerate the insertion of an extein scar (split SAC-PACE) (Fig. 2a, middle panel). We selected the fast-splicing gp41-8 intein pair^41,42^ as the Npu intein pair was already in use in the accessory plasmid. In overnight propagation assays, only host cells containing a psp-driven TadA8e-gp41-8N construct on a complementary plasmid enabled survival of SP expressing gp41-8C-dNme2Cas9 (Supplementary Fig. 11 and Supplementary Note 3). Since we can control the expression level of the TadA8e construct on the complementary plasmid, this result validated the ability of the split SAC-PACE selection to limit base editor concentrations while continuing to select for evolving Cas9-containing SP.

Using the intermediate-stringency split SAC-PACE selection, we further evolved Nme2Cas9 variants that had emerged from low-stringency selections. We pooled endpoint phage from ePACE1 and ePACE2 and cloned them into the split SP architecture, then seeded those SP into the split SAC-PACE selection (ePACE3) (Fig. 2b). All targeted PAMs exhibited moderate phage persistence (>10^5^ titers) within at least one lagoon at or above 2 volumes per hour (Supplementary Fig. 12). Sequenced clones from lagoons other than the one targeting an N_3_CTG PAM showed very strong mutational convergence across lagoons and PAMs, suggesting that the resulting Nme2Cas9 variants likely were not acquiring PAM specificity at the positions defined in our evolutions (PAM positions 4 and 6) (Supplementary Fig. 13a). ABE-PPE profiling of a representative variant from ePACE3 (E3-18-ABE8e) that had exhibited activity on N_4_TN PAM sites in mammalian cells (Supplementary Fig. 13b) showed comparable activity (31 and 39% average A•T-to-G•C conversion on N_4_CD and N_4_TN PAM sites, respectively) to the earlier evolved E2-12-ABE8e variant. However, this broadened PAM compatibility was again accompanied by a C preference at PAM position 7 (61 versus 33% average A•T-to-G•C conversion on N_4_YNC and N_4_YND PAM sites, respectively) (Extended Data Fig. 2e), indicating that restricting enzyme concentration alone is insufficient to evolve higher activity variants with desired PAM preferences.

Thus, we added another layer of stringency control to increase the likelihood of evolving higher activity variants. We implemented a multiplexed-PAM selection requiring correction of a stop codon in two protospacers flanked by PAM sequences with alternating sequence identity at PAM positions 1–3 and 7 (NNNNNNN), thereby forcing evolving Nme2Cas9 variants to recognize multiple nucleotides at undesired PAM positions. We coupled this selection with split SAC-PACE to produce a third (high-stringency) scheme that we term dual-PAM split SAC-PACE (Fig. 2b, right panel). With these developments, we could now pursue high-stringency evolutions along both trajectories (N_4_CN and N_4_TN PAM sequences).

### High-stringency evolution of Nme2Cas9 toward N_4_CN PAM sequences

The outcomes of ePACE1 and ePACE2 revealed that improved activity on N_4_TN PAMs was accompanied by broadened activity on N_4_CN PAMs. We hypothesized that the mutational diversity from these evolutions might provide useful starting points for the evolution of N_4_CN PAM compatibility. We thus pursued this trajectory with both wild-type Nme2Cas9 and pooled ePACE1 and ePACE2 (E1 + E2) phage, subjecting these starting points to high-stringency evolutions in parallel via dual-PAM split SAC-PACE (Fig. 2b).

**Extended Data Fig. 3:**
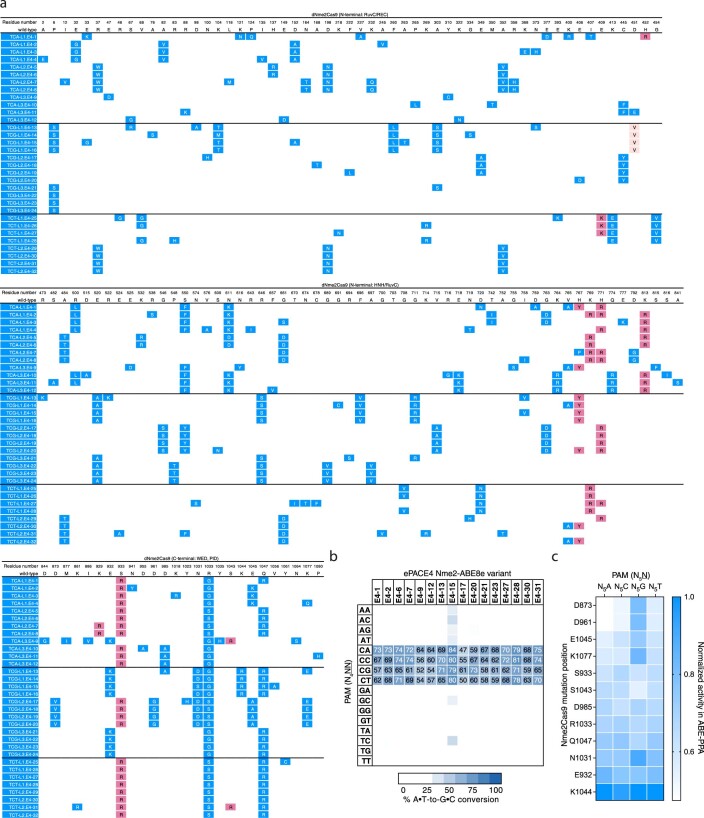
Mutation table and representative activity of ePACE4 evolved Nme2Cas9 variants. **(a)** Genotypes of individually sequenced plaques following ePACE4, with positions varying from wild-type displayed. Clones evolved on different PAMs are delineated by a bold line. Mutations that had previously appeared in ePACE1 and ePACE2 are shown in light pink and magenta, respectively, while novel mutations are shown in blue. **(b)** Heat map showing ABE-PPA activity of representative clones from ePACE4 on the 16 combinations of PAM positions 5 and 6 (N_4_NN) Values are raw % A•T-to-G•C conversion observed for one replicate of each editor and are listed in each cell for the N_4_CN PAMs, with values above 70% A•T-to-G•C conversion colored white. **(c)** ABE-PPA activity in (b) pooled and segregated by mutation position. Each column depicts the impact of a given position, when mutated, on ABE-PPA activity at each of the four PAM groups (N5A, N5C, N5G, N5T) (see Supplementary Note 4). Values are normalized against the highest activity within each set of PAMs. Only positions that were observed to be mutated more than once in (a) were included in this analysis.

SP containing either wild-type or E1 + E2 phage propagated insufficiently for PACE on N_4_CN-containing APs requiring dual edits. As such, we started evolution with PANCE, a non-continuous version of PACE in which phage are discretely passaged following an incubation period (typically overnight)^19^. Using PANCE (N1), we evolved either wild-type gp41-8C-dNme2Cas9 or pooled E1 + E2 endpoint phage on the set of six N_3_WCD (where W is A or T) PAMs (Fig. 2b and Supplementary Fig. 14). Following 20 passages in PANCE, only some of the lagoons targeting N_3_TCD PAMs appeared to consistently propagate. Phage from these lagoons were then seeded into ePACE (ePACE4) (Fig. 2b and Supplementary Fig. 15). Few mutations from E1 + E2 were retained in ePACE4, both within and outside the PID, suggesting evolution of a distinct mode of PAM recognition among ePACE4 clones (Extended Data Fig. 3a).

Sixteen ePACE4 clones assayed using ABE-PPA exhibited strong and general ABE activity, averaging 66% editing across all N_4_CN PAMs (Extended Data Fig. 3b and Supplementary Table 1). The E4-15 variant in particular, which we denote as eNme2-C (Nme2Cas9 P6S, E33G, K104T, D152A, F260L, A263T, A303S, D451V, E520A, R646S, F696V, G711R, I758V, H767Y, E932K, N1031S, R1033G, K1044R, Q1047R, V1056A), achieved ≥80% A•T-to-G•C editing at all N_4_CN PAM sites as an ABE8e, corresponding to a 4.8-fold average improvement in activity on N_4_CD PAM sites over Nme2-ABE8e, and a 1.3-fold average improvement in activity even on N_4_CC PAM sites natively recognized by wild-type Nme2Cas9 (Fig. 2c,d). Notably, activity improvements of ePACE4 variants on specific N_4_CN PAMs appeared to be largely agnostic of the specific PAM offered during evolution, with most variants preferring N_4_CA > N_4_CC > N_4_CT > N_4_CG (Extended Data Fig. 3b,c and Supplementary Note 4). ePACE4 variants (for example, eNme2-C, Fig. 2c) no longer exhibited the preference for a C at PAM position 7 exhibited in earlier evolved variants. Collectively, these findings establish that by requiring multiple PAM engagements, the dual-PAM split SAC-PACE selection can successfully generate high-activity Cas9 variants with broadened PAM scope.

**Extended Data Fig. 4:**
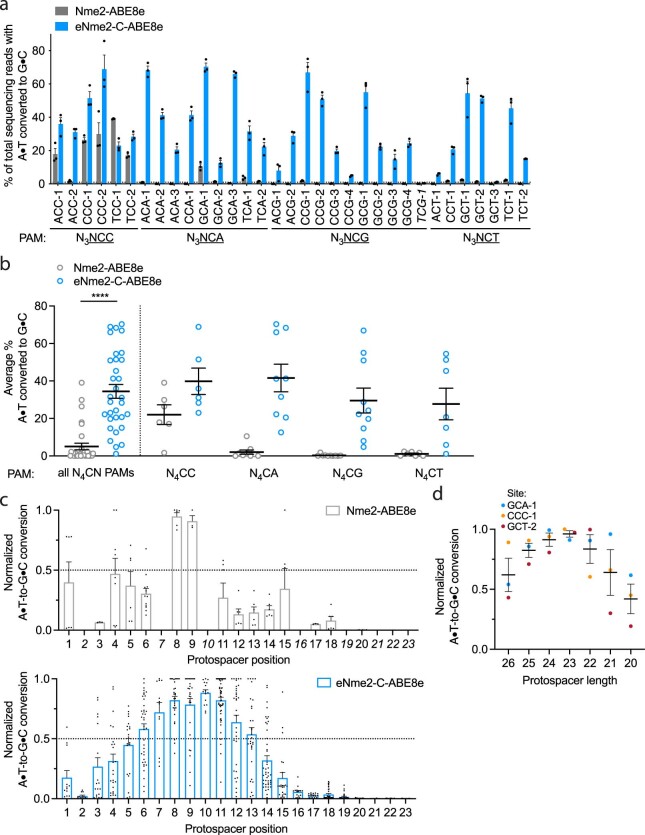
N4CN activity, editing window, and preferred spacer length of eNme2- C-ABE8e. **(a)** Adenine base editing activity of eNme2-C-ABE8e at 33 N_3_NCN PAM-containing sites in HEK293T cells. Mean±SEM is shown and reflects the average activity and standard error of *n* = 3 replicates at the maximally edited position within each genomic site. The site that exhibited <1% base editing activity (line shown) that was excluded in subsequent analyses is italicized. **(b)** Pooled adenine base editing activity of eNme2-C- ABE8e from (a). Left: all 32 sites with base editing >1% for eNme2-C-ABE8e; right: sites pooled by PAM position 6 identity. Each point represents the average editing of *n* = 3 independent biological replicates measured at a given genomic site. Mean±SEM is shown and reflects the average activity and standard error of the pooled genomic site averages. **(c)** Editing window of Nme2-ABE8e (top) or eNme2- C-ABE8e (bottom) reflective of pooled adenine base editing activity at all 23 protospacer positions (PAM counted as positions 24-29). Each point represents the % A•T-to-G•C conversion observed for an adenine that was present in one of the 32 protospacers, normalized to the highest editing observed within that protospacer. Italicized positions were not present in any protospacers evaluated. Mean±SEM is shown and reflects the average normalized activity and standard error at all observed adenines at that position. **(d)** Adenine base editing activity of eNme2-C-ABE8e as a function of protospacer length (between 26-20 nt) at three different genomic sites in HEK293T cells. Each point represents the average of *n* = 3 independent biological replicates observed for a given protospacer length at one genomic site, normalized to the protospacer length with the highest base editing activity for that site. Mean±SEM is shown and reflects the average normalized activity and standard error of the pooled averages at the observed sites. ****, p } 0.0001 by unpaired, two-tailed Student’s t-test.

Encouraged by the PAM profile of ePACE4 variants, we next tested whether the activity observed in bacterial cells successfully translated to mammalian cells. In HEK293T cells, we observed robust ABE activity for eNme2-C-ABE8e across all eight endogenous human genomic N_4_CN sites previously tested. Notably, eNme2-C-ABE8e showed 2.0-fold higher average editing efficiency on N_4_CC PAM sites and 15-fold higher editing efficiency on N_4_CD PAM sites than Nme2-ABE8e, and 2.3- to 3.3-fold improved editing at all sites compared to earlier evolved variants eNme2E1-2-ABE8e and eNme2E2-12-ABE8e, respectively (Fig. 2e). To further test the N_4_CN PAM generality of eNme2-C-ABE8e, we evaluated activity at an additional 25 genomic sites flanked by N_4_CN PAMs (for a total of 33 endogenous genomic sites tested) and observed an average of 34% A•T-to-G•C conversion at the tested sites exhibiting base editing above 1% (32 of 33 sites), a 1.8- and 30-fold average improvement at N_4_CC and N_4_CD PAM sites, respectively, over Nme2-ABE8e (Extended Data Fig. 4a,b). The editing window of eNme2-C-ABE8e is approximately between protospacer positions 9 and 16 (counting the PAM as positions 24–29) and is similar to the editing window of Nme2-ABE8e (Extended Data Fig. 4c). Like Nme2Cas9, eNme2-C retains a protospacer preference centered around 23 base pairs in length (Extended Data Fig. 4d). Together, the ABE-PPA data and this mammalian cell data suggest that eNme2-C-ABE8e is a robust adenine base editor that provides general access to N_4_CN PAMs.

### High-stringency evolution of Nme2Cas9 toward N_4_TN PAM sequences

**Extended Data Fig. 5:**
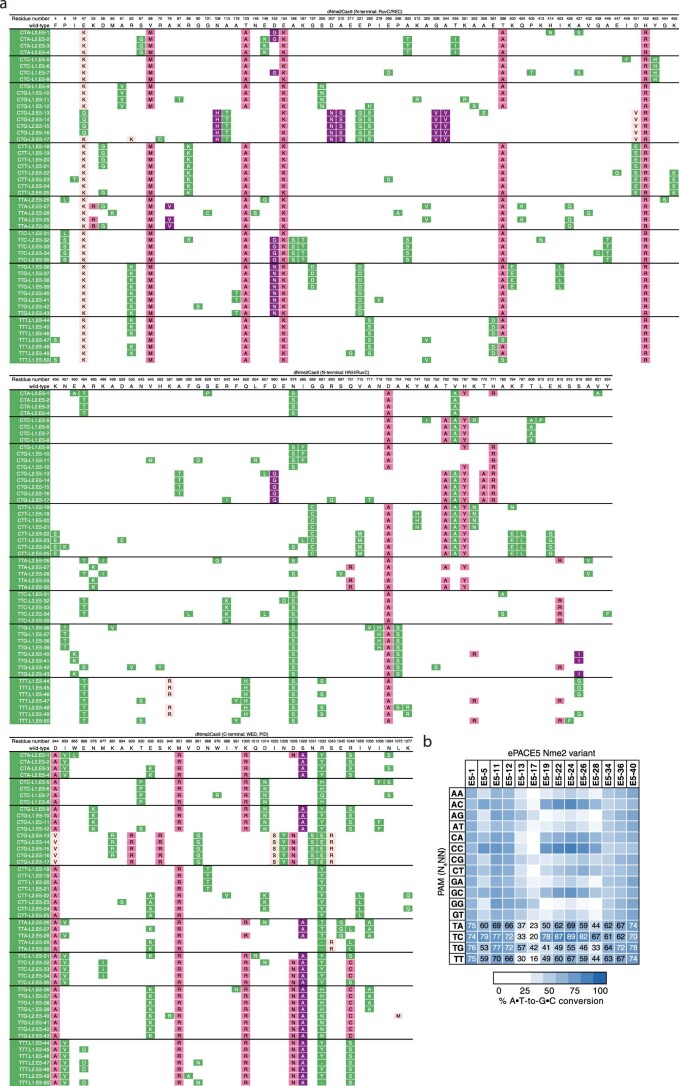
Mutation table and representative activity of ePACE5-evolved Nme2Cas9 variants. **(a)** Genotypes of individually sequenced plaques following ePACE5, with positions varying from wild-type displayed. Clones evolved on different PAMs are delineated by a bold line. Mutations that had previously appeared in ePACE1, ePACE2, or ePACE3 are shown in light pink, magenta, or purple, respectively, while novel mutations are shown in green. Positions that were unable to be called due to low sequencing quality are denoted by a “-“. **(b)** Heat map showing ABE-PPA activity of representative clones from ePACE5 on the 16 combinations of PAM positions 5 and 6 (N_4_NN) Values are raw % A•T-to-G•C conversion observed for one replicate of each editor and are listed in each cell for the N_4_TN PAMs, with values above 70% A•T-to-G•C conversion colored white.

Following the success of the N_4_CN trajectory using a high-stringency selection, we revisited the N_4_TN trajectory using a similar approach. Starting with PANCE (N2), we attempted to evolve three different pools of MP6-diversified phage on each of the eight N_3_YTN PAMs (Fig. 2b and Supplementary Fig. 16). Across eight PANCE passages, only lagoons seeded with ePACE3 endpoint phage propagated. These phage pools were subsequently seeded into ePACE (ePACE5). Under continuous evolution, these phage pools struggled to propagate, with phage washing out of many lagoons and only persisting with low titers (around 10^5^ pfu per ml) at low flow rates (<1.5 volumes per hour) among surviving lagoons (Supplementary Fig. 17). Phage clones were sequenced from each lagoon at a timepoint during which titers exceeded 10^5^ pfu per ml. Most sequenced clones retained many of the strongly converged mutations from ePACE3, particularly in the non-PID region. However, in the PID, we observed intra-lagoon convergence at residue 1033 (which mediates the wild-type interaction with the PAM position 6 cytosine and previously converged to lysine in ePACE3) and residue 1049 (positioned proximal to the PAM) for lagoons evolved on the same PAM, but divergence across PAMs (R1033Y/E/N/H/T; R1049S/L/C), suggesting PAM-specific interactions at positions 4 or 6 made possible by the higher stringency selection (Extended Data Fig. 5a).

Using ABE-PPA, we observed that ePACE5 variants exhibited broad PAM compatibility (Extended Data Fig. 5b and Supplementary Table 1), in contrast to ePACE4 variants that exhibited strong N_4_CN-specific activity. While N_4_TN activity was the most enriched, substantial adenine base editing activity was observed at all other PAMs, which could increase downstream Cas-dependent off-target editing. Two clones, E5-1, which we denote eNme2-T.1 (Nme2Cas9 E47K, V68M, T123A, D152G, E154K, T396A, H413N, A427S, H452R, E460A, A484T, S629P, N674S, D720A, V765A, H767Y, H771R, V821A, D844A, I859V, W865L, M951R, K1005R, D1028N, S1029A, R1033Y, R1049S, N1064S) and E5-40, which we denote eNme2-T.2 (Nme2Cas9 E47K, R63K, V68M, A116T, T123A, D152N, E154K, E221D, T396A, H452R, E460K, N674S, D720A, A724S, K769R, S816I, D844A, E932K, K940R, M951R, K1005R, D1028N, S1029A, R1033N, R1049C, L1075M), showed >70% average A•T-to-G•C editing across all N_4_TN PAMs as ABE8e variants (Fig. 2f and Extended Data Fig. 5b). As with the ePACE4 variants, many ePACE5 variants no longer exhibited a preference at PAM position 7 (for example, eNme2-T.1, eNme2-T.2, Fig. 2c), further highlighting the benefit provided by the multiplexed-PAM selection scheme.

**Extended Data Fig. 6:**
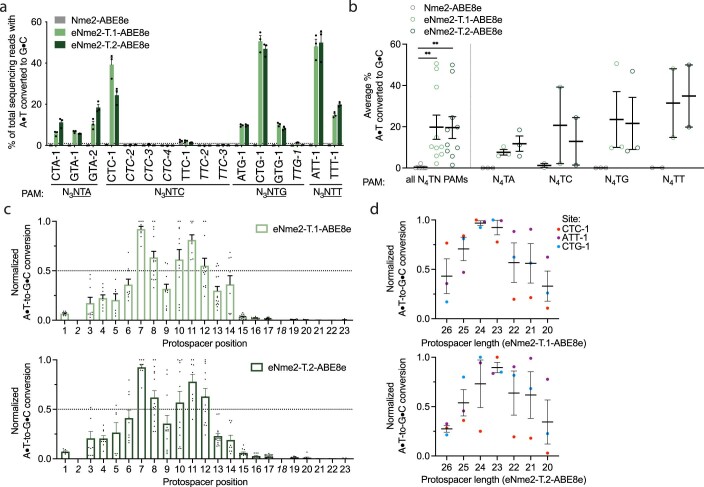
N4TN activity, editing window, and preferred spacer length of eNme2- T.1-ABE8e and eNme2- T.2-ABE8e. **(a)** Adenine base editing activity of eNme2-T.1-ABE8e and eNme2-T.2-ABE8e at 16 N_3_NTN PAM-containing sites in HEK293T cells. Mean±SEM is shown and reflects the average activity and standard error of *n* = 3 independent biological replicates at the maximally edited position within each genomic site. The six sites that exhibited <1% base editing activity for either variant (line shown) that were excluded in subsequent analyses are italicized. **(b)** Pooled adenine base editing activity of eNme2-T.1-ABE8e and eNme2-T.2-ABE8e from (a). Left: all 10 sites; right: sites pooled by PAM position 6 identity. Each point represents the average of *n* = 3 independent biological replicates measured at the maximally edited position within each given genomic site. Mean±SEM is shown and reflects the average activity and standard error of the pooled genomic site averages. **(c)** Editing window of eNme2-T.1-ABE8e (top) or eNme2-T.2-ABE8e (bottom) reflective of pooled adenine base editing activity at all 23 protospacer positions (PAM counted as positions 24–29) of the 10 sites shown in (a). Each point represents the % A•T-to-G•C conversion observed for an adenine that was present in one of the 10 protospacers, normalized to the highest editing observed within that protospacer. The italicized positions (2 and 18) were not present in any protospacers evaluated. Mean±SEM is shown and reflects the average normalized activity and standard error at all observed adenines at that position. **(d)** Adenine base editing activity of eNme2-T.1- ABE8e (top) or eNme2-T.2-ABE8e (bottom) as a function of protospacer length at three different genomic sites in HEK293T cells. Each point represents the average of *n* = 3 independent biological replicates observed for a given protospacer length at one genomic site, normalized to the protospacer length with the highest base editing activity for that site. Mean±SEM is shown and reflects the average normalized activity and standard error of the pooled averages at the observed sites. **, p } 0.01 by individual unpaired, two- tailed Student’s t-tests comparing Nme2-ABE8e to either eNme2-T.1-ABE8e (p = 0.0039) or eNme2-T.2-ABE8e (p = 0.0018).

We tested the eNme2-T.1 and eNme2-T.2 variants in HEK293T cells at the eight endogenous human genomic N_4_TN sites previously tested. At these eight sites, eNme2-T.1-ABE8e and eNme2-T.2-ABE8e averaged 23 and 22% A•T-to-G•C editing, respectively, representing a 278- and 264-fold improvement in activity over wild-type Nme2-ABE8e (Fig. 2g and Extended Data Fig. 6a,b). After including eight additional genomic N_4_TN sites, eNme2-T.1-ABE8e and eNme2-T.2-ABE8e exhibited base editing efficiencies above 1% at 69 or 63% of the 16 total sites, respectively. Within the sites showing >1% base editing, efficiencies ranged from 1.4 to 51% for eNme2-T.1-ABE8e and from 1.4 to 50% for eNme2-T.2-ABE8e. Both variants appeared to have a slightly 5′ shifted base editing window compared to eNme2-C-ABE8e, between positions 7 and 12 of the protospacer (counting the PAM as positions 24–29), but showed similar protospacer length preferences of 23 base pairs (Extended Data Fig. 6c,d).

While the N_4_TN activity of eNme2-T.1 and eNme2-T.2 were promising, ABE-PPE data (Fig. 2c) suggested that these two variants may also have activity on other PAM sites. To further characterize the PAM compatibility of these variants in mammalian cells, we evaluated eNme2-T.1-ABE8e and eNme2-T.2 at 22 genomic sites flanked by N_4_VN PAMs (where V is A, C or G). Consistent with their evolutionary histories and with ABE-PPE showing strongest enrichment for N_4_TN PAMs, activity on N_4_VN PAM sites was generally lower than on N_4_TN PAM sites and varied considerably from site to site (Supplementary Fig. 18). These mammalian cell editing data suggest that while eNme2-T.1-ABE8e and eNme2-T.1-ABE8e are capable of accessing N_4_TN PAMs and some other PAMs, editing efficiencies especially for the latter remain site-dependent. Together, these evolved variants from both trajectories (eNme2-C, eNme2-T.1 and eNme2-T.2) offer access to a large suite of pyrimidine-rich PAMs largely inaccessible to SpCas9-derived variants.

### Comparison of eNme2 and SpRY base editors and nucleases

**Fig. 3:**
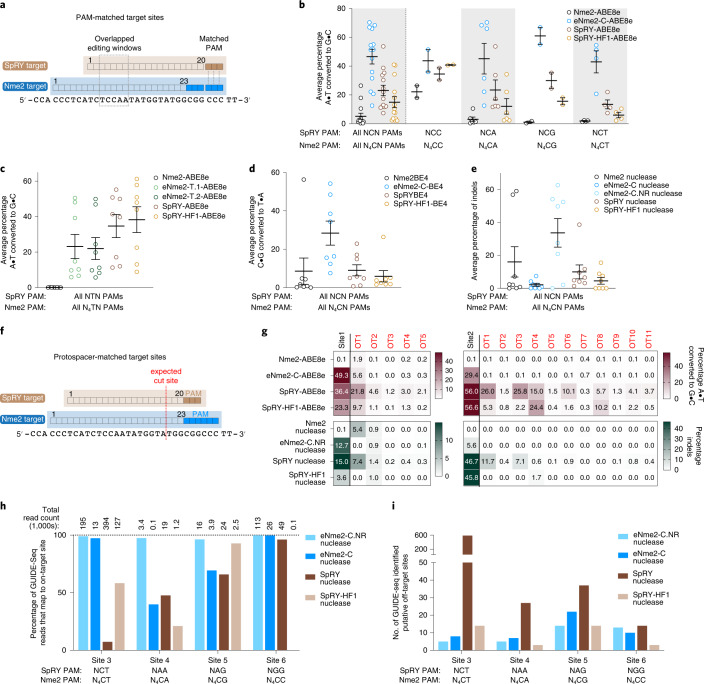
Characterization of evolved Nme2Cas9 variants in mammalian cells. **a**, Overview of PAM-matched sites used to compare eNme2Cas9 variants to SpRY and SpRY-HF1. **b**, Summary dot plots showing the activity of eNme2-C-ABE8e compared to SpRY-ABE8e and SpRY-HF1-ABE8e at 14 PAM-matched NCN/N_4_CN sites in HEK293T cells. Left-most data represent a summary of all 14 sites, and subsequent columns represent a subdivision into specific PAMs. **c**, Summary dot plots showing the activity of eNme2-T.1-ABE8e and eNme2-T.2-ABE8e compared to SpRY-ABE8e and SpRY-HF1-ABE8e at eight PAM-matched NTN/N_4_TN sites in HEK293T cells. **d**, Summary dot plots showing the activity of eNme2-C-BE4 compared to SpRY-BE4 and SpRY-HF1-BE4 at eight PAM-matched NCN/N_4_CN sites in HEK293T cells. **e**, Summary dot plots showing the activity of eNme2-C nuclease and eNme2-C.NR nuclease compared to SpRY nuclease and SpRY-HF1 nuclease at eight PAM-matched NCN/N_4_CN sites in HEK293T cells. **f**, Overview of protospacer-matched sites used to compare the DNA specificity of eNme2Cas9 variants against SpRY and SpRY-HF1. **g**, Heat maps showing off-target adenine base editing activity (brown) or off-target indel formation (dark green) at computationally determined off-targets for two sites in HEK293T cells for eNme2-C-ABE8e and eNme2-C.NR nuclease compared to SpRY and SpRY-HF1 adenine base editor and nuclease variants. The left-most column represents on-target activity. Values represent the average of *n* = 3 independent biological replicates. **h**, Percentage of on-target GUIDE-seq reads identified at four protospacer-matched sites for eNme2-C nuclease, eNme2-C.NR nuclease, SpRY nuclease and SpRY-HF1 nuclease. Total reads for the given nuclease are listed above each bar. **i**, Total putative off-target sites identified by GUIDE-seq for eNme2-C nuclease, eNme2-C.NR nuclease, SpRY nuclease and SpRY-HF1 nuclease at four protospacer-matched sites. For **b**–**e**, each point represents the average editing of *n* = 3 independent biological replicates measured at the maximally edited position within each given genomic site. Mean ± s.e.m. is shown and reflects the average activity and standard error of the pooled genomic site averages.

**Extended Data Fig. 7:**
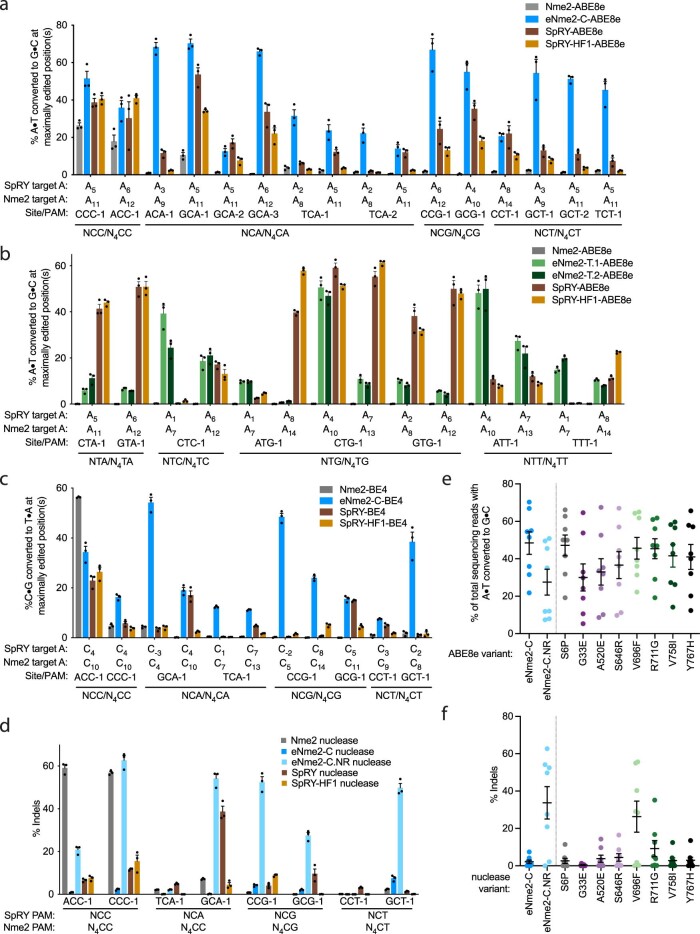
eNme2 variants compared to SpRY and SpRY-HF1 in HEK293T cells. **(a)** Adenine base editing activity of eNme2-C-ABE8e compared to SpRY-ABE8e and SpRY-HF1-ABE8e at 14 NCN/N4CN PAM-matched sites in HEK293T cells (pooled data in Fig. 3b). **(b)** Adenine base editing activity of eNme2-T.1-ABE8e and eNme2-T.2-ABE8e compared to SpRY-ABE8e and SpRY-HF1-ABE8e at eight NTN/N4TN PAM-matched sites in HEK293T cells (pooled data in Fig. 3c). **(c)** Cytosine base editing activity of eNme2-C-BE4 compared to SpRY-BE4 and SpRY-HF1-BE4 at eight NCN/N4CN PAM-matched sites in HEK293T cells (pooled data in Fig. 3d). **(d)** Nuclease activity of eNme2-C nuclease and eNme2-C.NR nuclease compared to SpRY nuclease and SpRY-HF1 nuclease at eight NCN/N4CN PAM-matched sites in HEK293T cells (pooled data in Fig. 3e). For (a-d), Mean±SEM is shown and reflects the average activity and standard error of *n* = 3 independent biological replicates measured at the maximally edited position (if applicable) within each given genomic site. **(e)** Pooled adenine base editing activity of eNme2-C-ABE8e compared to eNme2-C.NR-ABE8e or adenine base editors generated from reversion mutations at each of the eight RuvC/HNH domain mutations in eNme2-C at eight genomic sites in HEK293T cells. **(f)** Pooled nuclease activity of eNme2-C nuclease compared to eNme2-C.NR nuclease or nuclease-active variants generated from reversion mutations at each of the eight RuvC/HNH domain mutations in eNme2-C at eight genomic sites in HEK293T cells. For (e) and (f), each point represents the average of *n* = 3 independent biological replicates measured at the maximally edited position within each given genomic site in HEK293T cells. Mean±SEM is shown and reflects the average activity and standard error of the pooled genomic site averages.

Next, we compared the editing performance of evolved eNme2 variants with that of alternative Cas variants. No natural Cas variants capable of targeting single pyrimidine PAMs have been reported^3^. Among engineered Cas variants, only SpRY has shown activity on some NCN and NTN PAMs^7^. We selected PAM-matched genomic sites to directly compare the base editing activities of SpRY and eNme2 variants (Fig. 3a). At 14 matched C-containing PAM sites in HEK293T cells, eNme2-C-ABE8e showed a marked improvement in adenine base editing over SpRY, averaging 47 versus 23% A•T-to-G•C editing. This difference is more pronounced (47 versus 15% A•T-to-G•C editing) when compared to the ABE8e version of high-fidelity SpRY, SpRY-HF1-ABE8e (Fig. 3b and Extended Data Fig. 7a). By contrast, at eight matched T-containing PAM sites in HEK293T cells, eNme2-T.1-ABE8e and eNme2-T.2-ABE8e are less active than either SpRY-ABE8e or SpRY-HF1-ABE8e (23 and 22% for eNme2-T.1-ABE8e and eNme2-T.2-ABE8e versus 35 and 38% for SpRY-ABE8e or SpRY-HF1-ABE8e, respectively) (Fig. 3c and Extended Data Fig. 7b). These data indicate that eNme2-C offers a best-in-class option for modifying C-containing PAM sites, while eNme2-T.1 and eNme2-T.2 provide new options for targeting some T-containing PAMs together with the existing SpRY variants.

We then tested whether the improvements to Nme2Cas9 were generalizable to other Cas9-dependent editing modalities. At six PAM-matched target sites in HEK293T cells, eNme2-C-BE4 exhibited an average of 28% C•G-to-T•A editing, a 3.2- and 4.8-fold improvement over SpRY-BE4 and SpRY-HF1-BE4, respectively (Fig. 3d and Extended Data Fig. 7c). Although less efficient than eNme2-C-ABE8e, eNme2-C-BE4 is capable of C•G-to-T•A editing at levels comparable to (within twofold of) those reported for SpCas9 or SpCas9-derived CBE variants at their canonical purine-containing PAMs^7,9,11,43,44^.

When the RuvC-inactivating mutation D16A (ref. ^17^) was reverted, eNme2-C nuclease was inefficient at generating indels in mammalian cell culture, averaging only 2.1% indels at eight N_4_CN PAM sites (Fig. 3e and Extended Data Fig. 7d). We hypothesized that this was due to the large number of mutations in the RuvC and HNH domains of eNme2-C, some of which could be nuclease-inactivating. Indeed, when we reverted all mutations in the nuclease and associated linker domains, the resulting variant, eNme2-C.NR (eNme2-C S6P, G33E, A520E, S646R, V696F, R711G, V758I, Y767H) had restored nuclease activity while retaining new N_4_CN PAM activity (average 34% indels across the same eight sites). However, reversion of these mutations had a negative impact on ABE activity, with eNme2-C.NR-ABE8e exhibiting 1.8-fold reduced A•T-to-G•C conversion compared to eNme2-C-ABE8e (Extended Data Fig. 7e). These results indicate that some or all the mutations in the RuvC/HNH domains are important for robust base editing of the eNme2-C variant, but the same mutations, if present, are detrimental to the subsequent activation or catalytic activity of eNme2-C.NR nuclease (Extended Data Fig. 7e,f and Supplementary Note 5).

Having established two distinct subvariants of eNme2-C for either base editing or DNA cleavage, we next compared eNme2-C.NR nuclease to SpRY and SpRY-HF1 nucleases. Both SpRY and SpRY-HF1 nucleases were relatively inefficient at the NCN PAM-matched sites tested, being markedly outperformed by eNme2-C.NR nuclease (3.4- and 7.3-fold more efficient editing by eNme2-C.NR nuclease, respectively) (Fig. 3e and Extended Data Fig. 7d). Given this data, we speculate that perhaps some mutations in SpRY, such as with eNme2-C, may asymmetrically affect base editing versus nuclease activities (for instance sufficient R-loop formation for base editing but slow conformational shift for nuclease activation^45,46^). This hypothesis would also potentially explain why the activity observed for SpRY-ABE8e appears to be much more generalizable at NYN PAMs than what would be expected given the limited NYN PAM scope initially described for SpRY nuclease^7^. Together, these data highlight eNme2-C base editors and eNme2-C.NR nucleases as highly effective variants for genome editing, offering promising alternatives to SpRY and SpRY-HF1 in applications requiring access to C-containing PAMs.

### Off-target analysis reveals high genome-wide specificity of eNme2-C variants

PAM-broadened Cas variants have been shown to increase off-target activity due to the increased number of sequences recognized as a PAM^7,9,11^. While this off-target activity can be compensated for by introducing high-fidelity mutations that increase protospacer-target binding fidelity^7,47^, these mutations can sometimes result in a reduction in overall Cas activity (Fig. 3b,d,e comparing SpRY to SpRY-HF1 variants). Nme2Cas9 has been shown to be highly accurate, exhibiting very few if any off-targets compared to SpCas9 at protospacer-matched sites^17^. This higher specificity is potentially due to the longer protospacer requirement of Nme2Cas9 (22–23 (ref. ^17^) versus 20 nt), which naturally increases the total possible sequence space and decreases the occurrence of perfectly or near-perfectly matched off-target sites (Supplementary Fig. 19). Thus, we speculated that the C-PAM-specific eNme2-C may also be more specific than PAM-broadened SpCas9 variants.

To evaluate off-target activity, we first selected two protospacer-matched sites (sites 1 and 2) with validated nuclease and ABE activities for eNme2-C/eNme2-C.NR and SpRY variants (Fig. 3f). Using CHOPCHOPv3 (ref. ^48^), we used in silico prediction to identify the set of potential off-target sites with ≤2 mismatches and no more than one PAM-proximal (within 10 bp of the PAM) mismatch to at least one of the two protospacers (23 nt for Nme2Cas9, 20 nt for SpRY). We then evaluated off-target nuclease and ABE8e activities at all identified off-target sites (seven for site 1, 12 for site 2) using targeted amplicon sequencing (Supplementary Table 2).

For the site 1 protospacer, five of the seven predicted sites sequenced well, and eNme2-C-ABE8e showed off-target base editing >1% at one of these five sequenced off-target sites, while eNme2-C.NR did not generate any off-target indels >1% (Fig. 3g). In contrast, SpRY-ABE8e and SpRY-HF1-ABE8e exhibited off-target base editing >1% at all five or four of five sites, respectively, despite having lower on-target efficiency than eNme2-C-ABE8e. As nucleases, SpRY and SpRY-HF1 showed higher fidelity, with only two of five or one of five off-target site(s) exhibiting indels >1%, respectively. Similar trends were observed for the site 2 protospacer. No off-target base editing or indel formation >1% was observed at any of the 12 sequenced off-target sites for eNme2-C-ABE8e or eNme2-C.NR, whereas off-target base editing and indel formation >1% was observed at many sites for SpRY and SpRY-HF1. These data indicate that eNme2-C-ABE8e and eNme2-C.NR retain the high natural specificity of Nme2Cas9 and offer greater specificity than their SpRY and SpRY-HF1 counterparts, particularly for precision applications such as base editing.

**Extended Data Fig. 8:**
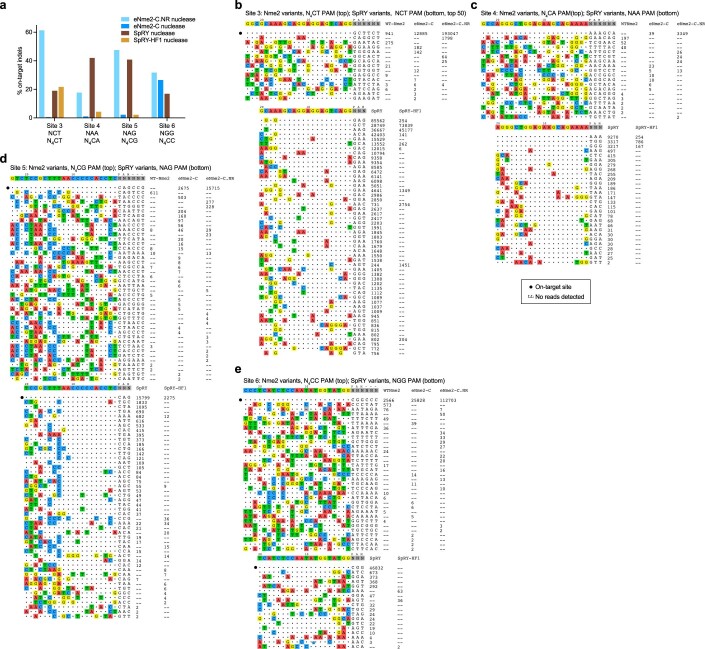
GUIDE-Seq identified off-targets of Nme2 variants compared to SpRY and SpRY-HF1. **(a)** On-target indel formation of wild-type Nme2 nuclease, eNme2-C nuclease, and eNme2-C.NR nuclease compared to SpRY nuclease and SpRY-HF1 nuclease at each of the four protospacer-matched sites that were subsequently evaluated in GUIDE-Seq. Each bar represents the observed indel formation of one replicate in U2OS cells. **(b-e)** GUIDE-Seq identified off-targets and associated read counts for Nme2 variants (top) or SpRY variants (bottom) at Site 3 (b), Site 4 (c), Site 5 (d), and Site 6 (e). The on-target protospacer is marked by a black dot for each site. Off-target thresholds were set at 8 mismatches with and NNN PAM for SpRY variants or 11 mismatches with an NNNNNN PAM for Nme2 variants).

To perform a more unbiased, genome-wide survey of potential off-targets, we used GUIDE-seq^49^ to evaluate double-strand breaks generated by eNme2-C.NR compared to SpRY variants at four protospacer-matched sites. Targeted sequencing of the on-target sites in treated U2OS cells showed robust indel formation at all four sites for both SpRY nuclease and eNme2-C.NR (30 and 40% indels for SpRY nuclease and eNme2-C.NR nuclease, respectively). Despite three of the four sites containing NRN-PAMs, SpRY-HF1 nuclease only generated >10% indels at the fourth site containing an NCN PAM. We also included the nuclease-active version of eNme2-C, although as expected indel formation was inefficient (<10%) at all but one site (Extended Data Fig. 8a). Across all four sites, eNme2-C.NR exhibited high specificity, averaging 52-to-1 on-to-off-target reads, compared to SpRY that averaged a 1.2-to-1 on-to-off-target ratio (Fig. 3h, Extended Data Fig. 8b–e and Supplementary Table 3). These specificity values corresponded to a range of 7 to 22 putative off-target sites for eNme2-C.NR versus 14 to 591 putative off-target sites for SpRY. At the site on which it was active, eNme2-C similarly exhibited minimal off-target activity. In contrast, while SpRY-HF1 exhibited higher specificity than SpRY at the site on which it was active (site 3), it still induced substantial off-target editing compared to eNme2-C.NR (Fig. 3i). We sequenced the top GUIDE-seq-nominated loci for eNme2-C, eNme2-C.NR, SpRY and SpRY-HF1 (Supplementary Table 3). In agreement with the GUIDE-seq data, both SpRY and SpRY-HF1 exhibited off-target nuclease and adenine base editing at more sites than either eNme2-C.NR nuclease or eNme2-C-ABE8e (Supplementary Fig. 20).

Similarly, we performed sequencing at in silico-nominated off-target sites for protospacer-matched sites comparing eNme2-T.1-ABE8e and eNme2-T.2-ABE8e to SpRY-ABE8e and SpRY-HF1-ABE8e (Supplementary Table 4). As with eNme-C, both eNme2-T variants exhibited off-target base editing at fewer sites than either SpRY or SpRY-HF1 (Supplementary Fig. 21). Together, these results indicate that evolved Nme2Cas9 variants may offer improved specificity compared to SpRY variants.

### eNme2-C is active in multiple mammalian cell types and enables access to both existing and new target single-nucleotide polymorphisms (SNPs)

**Fig. 4:**
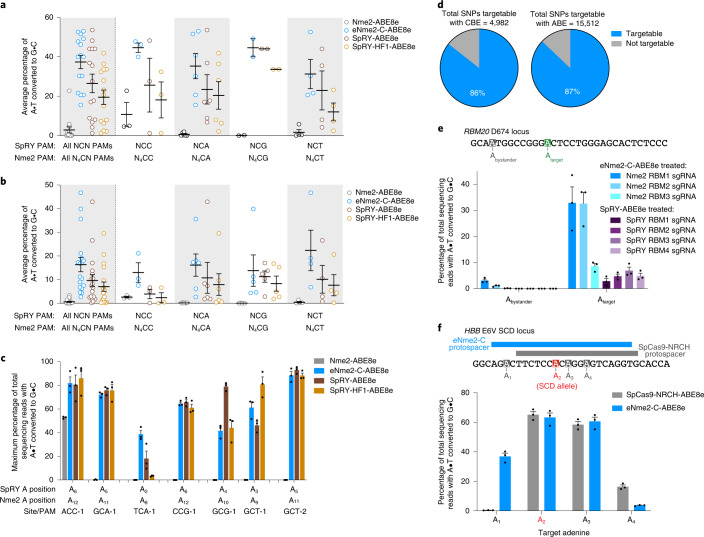
Generalizability of eNme2-C-ABE8e across different cell types and targets. **a**, Summary dot plots showing the activity of eNme2-C-ABE8e compared to SpRY-ABE8e and SpRY-HF1-ABE8e at 15 PAM-matched NCN/N_4_CN sites in HUH7 cells. Left-most data represent a summary of all 15 sites, and subsequent columns represent a subdivision into specific PAMs. **b**, Summary dot plots showing the activity of eNme2-C-ABE8e compared to SpRY-ABE8e and SpRY-HF1-ABE8e at 18 PAM-matched NCN/N_4_CN sites in U2OS cells. Left-most data represent a summary of all 18 sites, and subsequent columns represent a subdivision into specific PAMs. For **a** and **b**, each point represents the average editing of *n* = 3 independent biological replicates measured at the maximally edited position within each given genomic site. Mean ± s.e.m. is shown and reflects the average activity and standard error of the pooled genomic site averages. **c**, eNme2-C-ABE8e compared to SpRY-ABE8e and SpRY-HF1-ABE8e at eight PAM-matched NCN/N_4_CN sites in HDFa cells. Bars represent mean ± s.e.m. of *n* = 3 independent biological replicates, with individual values shown as dots. **d**, ClinVar identified SNPs that can be targeted with an eNme2-C-ABE8e (top) or eNme2-C-BE4 (bottom). **e**, Installation of a disease-relevant D674G mutation in the *RBM20* gene. Tiled guides were used to install the mutation either with eNme2-C-ABE8e or SpRY-ABE8e (see Supplementary Table 5 for sgRNA sequences). **f**, Conversion of the sickle-cell disease-causing *HBB* E6V mutation to the benign E6A (Makassar hemoglobin) allele using either SpCas-NRCH-ABE8e or eNme2-C-ABE8e. Bars represent mean ± s.e.m. *of n* = 3 independent biological replicates, with individual values shown as dots.

**Extended Data Fig. 9:**
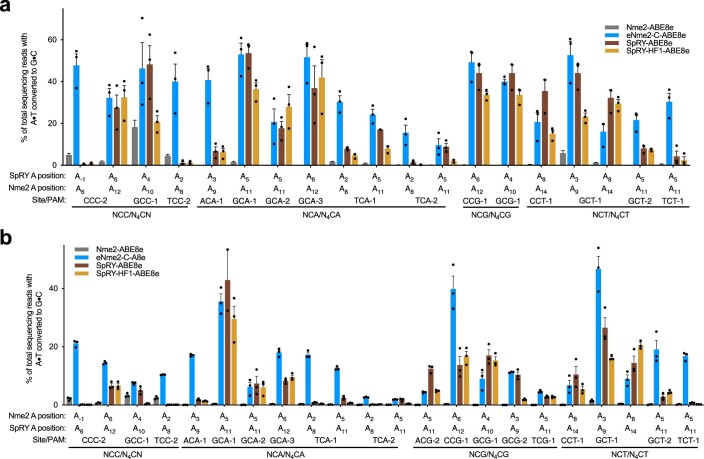
eNme2-C-ABE8e activity in other human cell types. **(a)** Adenine base editing activity of eNme2-C-ABE8e compared to SpRY-ABE8e and SpRY-HF1-ABE8e at 15 NCN/N4CN PAM-matched sites in HUH7 cells (pooled data in Fig. 4a). **(b)** Adenine base editing activity of eNme2-C-ABE8e compared to SpRY-ABE8e and SpRY-HF1-ABE8e at 18 NCN/N4CN PAM-matched sites in U2OS cells (pooled data in Fig. 4b). For (a) and (b), mean ± SEM is shown and reflects the average activity and standard error of *n* = 3 independent biological replicates measured at the maximally edited position within each genomic site.

Having validated the high efficacy and specificity of eNme2-C at target sites containing N_4_CN PAMs, we next demonstrated its generalizability in multiple cell types. In an immortalized hepatocyte cell line, HUH7, eNme2-C-ABE8e retains its broad base editing activity across sites containing N_4_CN PAMs, accessing all 15 sites tested with an average of 37% A•T-to-G•C base editing (Fig. 4a and Extended Data Fig. 9a). Similarly, at 18 sites in U2OS cells, adenine base editing activity was seen at all sites, albeit at lower average efficiency (averaging 16% A•T-to-G•C editing) (Fig. 4b and Extended Data Fig. 9b). In both cell types, eNme2-C-ABE8e outperforms SpRY-ABE8e and SpRY-HF1-ABE8e, although the extent varies. Finally, we nucleofected primary human dermal fibroblasts with eNme2-C-ABE8e messenger RNA, achieving 64% A•T-to-G•C base editing across seven endogenous sites (Fig. 4c). We observed that eNme2-C-ABE8e, SpRY-ABE8e and SpRY-HF1-ABE8e perform equally well in this cell line with nucleofection, potentially due to the high efficacy of mRNA nucleofection (Supplementary Fig. 22)^4,9^. Together, these data demonstrate that eNme2-C is a broadly applicable Cas protein enabling precision genome editing in multiple biologically relevant cell types.

Because of its N_4_CN PAM activity, eNme2-C is in theory perfectly complementary to single-G recognizing SpCas9 variants SpCas9-NG (ref. ^11^) and SpG^7^, which are estimated to enable potential cleavage around every 2.2 bp in the human coding sequence^11^. As a cytosine or adenine base editor, eNme2-C enables access to 86 and 87% of pathogenic transition SNPs, respectively, recognized in the ClinVar database (Fig. 4d)^50,51^. Although SpRY base editors should access similar PAMs due to their near-PAMless nature, we hypothesized that differences in editing windows and specific PAM compatibilities would enable eNme2-C base editors to not only serve as higher-fidelity alternatives to SpRY base editors, but also facilitate access to new targets.

*RBM20* is a gene encoding a *trans*-activating splicing factor, and mutations in the gene have been observed in 2–3% of familial dilated cardiomyopathy cases^52^. While many mutations have been identified in the coding sequence of *RBM20*, the individual effect of these mutations have not been well characterized, potentially due to the difficulty of installing some of these mutations in isolation. We used eNme2-C-ABE8e to install the D674G mutation, an A•T-to-G•C transition in which the target base is upstream of a stretch of pyrimidine bases inaccessible to most characterized Cas variants. All three eNme2-C-ABE8e guides tested enabled editing of the target adenine, with the optimal guide reaching 33% A•T-to-G•C base editing. By contrast, none of the four SpRY guides placing the target adenine in the optimal editing window of SpRY (positions 4–7)^4^ were able to achieve >10% A•T-to-G•C conversion (Fig. 4e and Supplementary Table 5). These data demonstrate that eNme2-C enables the study and potential correction of previously inaccessible pathogenic SNPs.

Finally, we examined whether eNme2-C-ABE8e could edit previously targeted therapeutically relevant loci with reduced off-target editing frequencies. Adenine base editing of the sickle-cell allele (*HBB*^*S*^) results in the benign hemoglobin Makassar allele and can rescue sickle-cell disease in animals^5,53^. While a previously evolved SpCas9 variant, SpCas9-NRCH, can efficiently install the Makassar allele in both cell culture and mouse models^5,9^, off-target base editing was observed at several Cas-dependent off-target sites.

We tested whether the highly specific nature of eNme2-C may yield a more favorable off-target editing profile at this locus. In a HEK293T cell line containing the SCD E6V mutation^54^, an eNme2-C-ABE8e sgRNA resulted in comparable editing efficiency of the target adenine compared to optimized SpCas9-NRCH-ABE8e and sgRNA (63% versus 65% A•T-to-G•C conversion, respectively) (Fig. 4f and Supplementary Table 5). Due to the slightly shifted editing window of eNme2-C-ABE8e, we observed higher editing at an upstream bystander adenine and lower editing at a downstream bystander adenine. With respect to off-target activity, we observed much higher specificity for eNme2-C-ABE8e compared to SpCas9-NRCH-ABE8e when targeting this site. Using the same in silico criteria described above, we selected nine predicted off-target sites for eNme2-C and 11 predicted off-target sites for SpCas9-NRCH (Supplementary Table 6). Across the nine predicted off-target sites for eNme2-C-ABE8e, off-target base editing was observed at only two of the nine predicted sites and neither exceeded 10% A•T-to-G•C conversion. In contrast, off-target editing with SpCas9-NRCH-ABE8e was observed at five of 11 predicted sites and averaged 33% A•T-to-G•C conversion across those five sites (Supplementary Fig. 23). Together, these data further support that eNme2-C not only expands the targeting scope of base editors but may also offer a more site-specific alternative to existing Cas9 variants.

## Discussion

By integrating a functional Cas enzyme selection (SAC-PACE) with high-throughput phage-assisted evolution platforms (PANCE and ePACE) and a high-throughput PAM-profiling method (BE-PPA) to guide our evolutionary campaign, we demonstrated evolution of a non-*S. pyogenes* Cas protein to acquire single-nucleotide PAM recognition. We developed two highly efficient, highly specific Nme2Cas9 variants capable of targeting N_4_CN PAM sequences across different gene editing modalities and two variants capable of adenine base editing at many N_4_TN PAM sequences, affording unparalleled access to pyrimidine-PAM sequences. Together, these variants complement the suite of commonly used SpCas variants and will enable the study and potential correction of previously inaccessible or poorly accessible loci, while retaining the compact size and high genome-wide specificity of Nme2Cas9 that could be beneficial to downstream clinical applications.

In contrast to previous Cas9 evolutions that selected for new PAM binding^8,9^, SAC-PACE requires both new PAM binding and subsequent activation steps necessary for base editing, increasing the likelihood of evolving desired editing properties. In addition to developing this new selection, we found that improvements analogous to those made to evolve high-activity SpCas9 variants could be easily incorporated into SAC-PACE, including limiting the concentration of active base editor through a split-intein system and requiring multiple editing events through the inclusion of additional base editing sites. Notably, the evolution campaign that resulted in eNme2-C generated substantially improved activity on N_4_CC PAMs, the PAMs recognized by the wild-type protein, along with numerous mutations outside the PID that appeared to contribute to this improved activity. This outcome supports the hypothesis that a functional selection enables improved evolution outcomes, in particular for Cas variants with lower starting activity^13^. These selections should be broadly adaptable to the evolution of any Cas ortholog toward new PAMs, and the sequence-agnostic nature of the target site can be applied to evolving new editing windows or disease-specific contexts.

Our development of ePACE facilitated parallel, automated and fully continuous evolution of Nme2Cas9 on multiple PAMs, overcoming many of the design, operation and infrastructural challenges of traditional PACE and adding to a growing set of automated directed evolution systems^31,32^. Notably, precise fluidic control was achieved using customizable, millifluidic IPP devices that can be readily and inexpensively manufactured in the laboratory to automate the fluidic handling needs of PACE, further reducing the need for intervention and enhancing scalability. ePACE can be further customized by modifying the millifluidics and eVOLVER smart sleeves to accommodate fewer chemostats feeding additional lagoons, thereby increasing the potential throughput of ePACE on a single eVOLVER base unit. This would be especially useful for PACE selections in which the same accessory plasmid can be used while the SP or media conditions are varied across lagoons. Additionally, given the highly reconfigurable nature of eVOLVER, it would be relatively simple to modify the smart sleeves to allow for smaller volumes (around 1 ml) for PACE experiments that rely on expensive media additives to save on costs. Taken together, we believe these technical developments to systematize PACE in a low-cost format coupled with eVOLVER’s flexibility for enabling new experimental dimensions will lower the barrier to entry for labs interested in applying PACE.

We modulated selection stringencies during ePACE experiments based on discrete quantitative PCR (qPCR) phage titer estimations. However, an exciting future prospect for ePACE is to develop and run ‘algorithmic selection routines’ that autonomously adjust selective pressures for individual PACE cultures based on real-time monitoring and feedback from the evolving population. Indeed, it is possible to estimate phage titers in PACE through coupling a luminescence readout to *gIII* transcription^55^. Additional incorporation of automated feedback based on luminescence in ePACE would further improve the ability to traverse evolutionary landscapes by lowering the lag time between titer readouts and stringency modulation, minimizing the need for researcher interaction and decision-making during experiments.

While we provided ePACE lagoons with the opportunity to evolve activity on specific PAM variants (for example, four separate lagoons for each N_4_CN PAM), variants emerged that were broadly active on the PAM position 5 base that was targeted (C or T). This outcome is expected for selection schemes that select for new activity but do not counter-select against undesired activities. Nevertheless, predicting which target PAM would yield eNme2-C, eNme2-T.1 or eNme2-T.2 a priori likely would have been difficult, as starting activity of wild-type Nme2Cas9 on any N_4_CN or N_4_TN is comparably low. This challenge highlights the strength of the ePACE platform, which enabled us to explore all trajectories in parallel, greatly enhancing the rate at which we were able to discover high-activity variants (five ePACE versus 20 to 40 traditional PACE experiments). Subsequent incorporation of a counter-selection^55^ against undesired PAMs in an ePACE-enabled parallel manner may result in highly PAM- or protospacer-specific Cas variants that further advance tailor-made genome modifying technologies.

## Methods

### General methods

Antibiotics (Gold Biotechnology) were used at the following working concentrations: carbenicillin 50 µg ml^−1^, chloramphenicol 25 μg ml^−1^, kanamycin 50 μg ml^−1^, tetracycline 10 μg ml^−1^ and streptomycin 50 μg ml^−1^. Nuclease-free water (Qiagen) was used for PCR reactions and cloning. All PCR reactions were carried out using Phusion U Hot Start polymerase (Thermo Fisher Scientific) unless otherwise noted. All plasmids and SP described in this study were cloned by USER assembly unless otherwise noted. Primers and gene fragments used for cloning were ordered from Integrated DNA Technologies (IDT) or Eton Biosciences, as necessary. For cloning purposes, Mach1 (Thermo Fisher Scientific) cells were used, and subsequent plasmid purification was done with plasmid preparation kits (Qiagen or Promega). Illustra TempliPhi DNA Amplification Kits (Cytiva) were used to amplify cloned plasmids before Sanger sequencing. For all phage related experiments (phage cloning, phage propagation, PACE and PANCE experiments) were done in parent *E. coli* strain S2060. Lists of plasmids, SP, protospacer sequences and primers used in this study are provided in Supplementary Tables 5 and 7–9.

### Overnight phage propagation assay

Chemicompetent S2060 cells were transformed with the AP(s) and complementary plasmid(s) of interest as previously described. Single colonies were subsequently picked and grown overnight in Davis Rich Media (DRM) with maintenance antibiotics at 37 °C with shaking, then back-diluted 200–1,000-fold into fresh DRM the next day and grown. On reaching optical density (OD_600_) 0.4–0.6, host cells were transferred into 500-µl aliquots and infected with 10 µl of desired SP (final titer 1 × 10^5^ pfu per mlf). Cells were then incubated for another 16–20 h at 37 °C with shaking, then centrifuged at 3,600*g* for 10 min. The supernatant containing phage was stored until use.

### Plaque assay

S2060 cells transformed with pJC175e (S2208, ref. ^19^) were used for plaque assays unless otherwise stated. To prepare a cell stock, an overnight culture of S2208s was diluted 50-fold into fresh 2xYT media with carbenicillin (50 µg ml^−1^) and grown at 37 °C to an OD_600_ of around 0.6–0.8. SP were serially diluted (four dilutions: 1:10 first dilution from concentrated phage stocks, then 1:100 remaining three dilutions) in DRM. Next, 10 µl of each dilution was added to 150 µl of cells, followed by addition of 850 µl of liquid (55 °C) top agar (2xYT media + 0.4% agar) supplemented with 2% Bluo-gal (1:50, final concentration 0.04%, Gold Biotechnology). These mixtures were then pipetted onto one quadrant of a quartered Petri dish containing 2 ml of solidified bottom agar (2xYT media + 1.5% agar, no antibiotics). Plates were allowed to briefly solidify before being incubated at 37 °C overnight without inversion.

### qPCR estimation of phage titer

When noted, phage titers were estimated by qPCR rather than plaque assay. SP pools (50 µl) were first heated at 80 °C for 30 min to destroy polyphage. Polyphage genomes were then degraded by adding 5 µl of heated SP to 45 µl of 1× DNase I buffer containing 1 µl of DNase I (New England Biolabs) and incubated at 37 °C for 20 min followed by 95 °C for 20 min. Next, 1.5 µl of each prepared phage DNA stock was then added to a 25 µl of qPCR reaction, prepared as follows: 10.5 µl of H_2_O, 12.5 µl 2× Q5 Mastermix (New England Biolabs), 0.25 µl of Sybr Green (Thermo Fisher Scientific), 0.125 µl of each primer (qPCR forward, 5′-CACCGTTCATCTGTCCTCTTT and qPCR reverse, 5’-CGACCTGCTCCATGTTACTTAG, Supplementary Table 7). qPCR was then run with the following cycling conditions: 98 °C for 2 min, 45 cycles of 98 °C for 10 s, 60 °C for 20 s and 72 °C for 15 s. Titers were calculated using a titration curve of an SP standard of known titer (by plaque assay). A limit of detection was set based on when primers amplified (without SP) or at the lowest titer before loss of linearity for the SP standard.

### PANCE

Chemically competent S2060s were transformed with the AP(s) and complementary plasmid(s) of interest along with a mutagenesis plasmid (MP6, ref. ^39^), and plated on 2xYT agar containing maintenance antibiotics and 100 mM glucose. Three colonies were subsequently picked into DRM with maintenance antibiotics and grown at 37 °C with shaking to an OD_600_ of around 0.4–0.6. Host cells were transferred into a 96-well plate in 500-µl aliquots, 10 mM arabinose was added to induce mutagenesis and SP dilutions were added according to the dilution schedules described in Supplementary Figs. 14 and 16 for N1 and N2, respectively. Cells were grown for 12–16 h at 37 °C with shaking, and subsequent SP were isolated in the supernatant following centrifugation at 3,600*g* for 10 min. To increase and diversify phage titers when necessary, SP were passaged in S2208s containing MP6 by infecting for 6–8 h. All SP titers were estimated by qPCR as described above.

### ePACE

#### General ePACE methods

eVOLVER and PACE were run as previously described^19,20^ with the following modifications. Chemostats were inoculated to OD_600_ of 0.05 and run at 30 ml total volume at 1 volume per hour until OD stabilization. The volume of lagoons was set to 10 ml via continuous pumping of waste with a high flow rate (45 ml min^−1^) peristaltic pump (SQ2349291, FynchBio) from a four-inch hypodermic needle (Air-Tite N224) (Supplementary Fig. 1). Cells were pumped in through using a slow flow rate (1 ml min^−1^) peristaltic pump (SQ2112453, FynchBio) and arabinose was pumped in using an IPP device. On infection with phage of interest (Supplementary Table 7), cell flow rates were set to the stated rates and arabinose flow rate was set to 0.04 volume per hour. Sampling and decisions on flow rate modifications were done as previously described^19^ (Supplementary Figs. 5, 7, 12, 15 and 17). Phage titer was quantified via the qPCR method described above.

#### Millifluidic fabrication

All IPP and pressure regulator millifluidic devices were constructed as previously described^20^. Briefly, fluidic designs were drawn out in EAGLE (Autodesk) and patterned onto 1/4- and 1/8-inch acrylic using a 40 W CO_2_ laser cutter (Epilog Mini 24). These layers were then plasma treated (Harrick Plasma, 30 W Expanded Plasma Cleaner) and bonded together using an optically clear laminating adhesive sheet (3M, 8146-3) with a silicone membrane (0.01 inch, Rogers Corporation, BISCO HT-6240) between them that enables valve actuation.

#### IPP calibrations

To calibrate IPP devices, bottles containing 1 l of water were attached to the input and pressurized to 1.5 psi. IPPs were controlled via three-way solenoid valves (S10MM-31-12-3, Pneumadyne) connected to the custom eVOLVER pressure regulator supplying 8 psi (Supplementary Fig. 3). Pumps were run at four different actuation frequencies long enough for at least 100 μl of water to flow, and then measured via pipette. A function of the form *y* = *kx*^a^ is then fit to the resulting data and used to set flow rates in subsequent experiments.

### Base editing-dependent PAM profiling

#### Cloning of BE-PPA libraries

Cloning of the library plasmids (pTPH342 for CBE-PPA, pTPH424 for ABE-PPA, Supplementary Table 7) was done via one-piece USER assembly of purified PCR product amplified using a primer pool containing all desired PAM sequences (IDT). Purified PCR product was aliquoted into two 0.2 pmol USER reactions (around 500 ng of a 4.2 kb fragment each), purified following USER digestion with PB buffer (Qiagen) and subsequent PE buffer washes (4×, Qiagen), and then eluted into 15 µl of H_2_O. The entire amount was then transformed into electrocompetent 10B cells (New England Biolabs), enough to yield at minimum 14× coverage^56^ of the expected library size. Electroporation was done in 25-µl aliquots using bacterial program X_13 in the 96-well Shuttle Device component of a 4D-Nucleofector system (Lonza). Transformed cells were immediately transferred to 1.5 ml (per 100 µl cells) of prewarmed SOC media. A serial dilution of the transformed cells (eight dilutions, fivefold each, starting with undiluted cells) was immediately taken and plated on maintenance antibiotics, which was used to calculate effective library size. The remaining cells are allowed to recover at 37 °C with shaking for 1 h before plating on 2xYT agar containing maintenance antibiotic. The following day, colonies were scraped and DNA was isolated using a Plasmid Plus Midi Kit (Qiagen).

#### Base editing-dependent PAM-profiling assay

Chemicompetent 10-beta cells (New England Biolabs) were transformed with the base editor variants of interest. Three colonies of each base editor variant are seeded into 10 ml of fresh DRM with maintenance antibiotic and grown at 37 °C with shaking to an OD_600_ around 0.4–0.6. On reaching the desired cell density, cells were spun down at 5,000*g* for 10 min, washed three times with ice-cold 10% (v/v) glycerol, then resuspended in a final volume of 100 µl of 10% glycerol. Next, 1 µg of library plasmid (pTPH342 or pTPH424) was added to these 100 µl aliquots, then transformed in 25 µl aliquots using bacterial program X_5 in the 96-well Shuttle Device component of a 4D-Nucleofector system. Transformed cells were immediately transferred to 1.5 ml (per 100 µl of cells) of prewarmed SOC media. A serial dilution of the transformed cells (eight dilutions, fivefold each, starting with undiluted cells) was immediately taken and plated on maintenance antibiotics, which was used to calculate effective library size. The remaining cells were allowed to recover at 37 °C with shaking for 15 min, then diluted into 40 ml of prewarmed DRM containing maintenance antibiotics and 10 mM arabinose. Induced cells were then grown at 37 °C with shaking for 22 h (ABE-PPA), or for 32 h with a 1:40 back-dilution at 16 h (CBE-PPA) before being collected by centrifugation at 3,600*g* for 10 min. DNA was isolated from collected cells using a Plasmid Plus Midi Kit (Qiagen).

#### High-throughput DNA sequencing

Library samples were prepared for high-throughput amplicon sequencing in two PCR steps. The first PCR (PCR1) was performed using forward primer BE-PPA-Fw and reverse primer BE-PPA-Rv (Supplementary Table 8) at a 150 µl scale and 1 µg of template DNA. Cycling conditions were as follows: 98 °C for 2 min, then 14 cycles of 98 °C for 15 s, 60 °C for 15 s, 72 °C for 20 s and a final extension at 72 °C for 2 min. A run of 14 cycles for PCR1 was observed to be within the linear amplification range for the libraries used in this study but may change for alternative library constructions. Following PCR1, PCR reactions were purified using the QIAquick PCR Purification Kit (Qiagen) and eluted in 16 µl of nuclease-free H_2_O. The second PCR (PCR2) was performed using forward and reverse Illumina barcoding primers at a 75 µl scale and half (8 µl) of the PCR1 purified product. Cycling conditions were as follows: 98 °C for 2 min, then eight cycles of 98 °C for 15 s, 60 °C for 15 s, 72 °C for 20 s and a final extension at 72 °C for 2 min. A run of eight cycles for PCR2 was observed to be within the linear amplification range for the libraries used in this study but may change for alternative library constructions. PCR2 products were pooled, purified by electrophoresis with a 1% agarose gel using a QIAquick Gel Extraction Kit (Qiagen) and eluted in nuclease-free H_2_O. DNA concentration was quantified with the KAPA Library Quantification Kit-Illumina (KAPA Biosystems) and sequenced on an Illumina MiSeq instrument (paired-end read R1 210 cycles, R2 0 cycles) according to the manufacturer’s protocols.

#### Analysis of BE-PPA HTS data

Sequencing reads were demultiplexed using the MiSeq Reporter (Illumina). Demultiplexed files were subsequently analyzed for base editing activity using a custom workflow combining the SeqKit^57^ and CRISPResso2 (ref. ^58^) packages. See Supplementary Note 6 for additional details. Post-CRISPResso2 analyzed nucleotide frequencies are listed in Supplementary Table 1.

#### Cell culture

HEK293T cells (ATCC CRL-3216), SCD allele containing HEK293T cells^54^ and HUH7 cells were cultured in Dulbecco’s modified Eagle’s medium plus GlutaMax (DMEM) (Thermo Fisher Scientific) supplemented with 10% (v/v) fetal bovine serum (FBS, Thermo Fisher Scientific). U2OS cells were cultured in McCoy’s 5A Medium (Thermo Fisher Scientific) supplemented with 10% (v/v) FBS. Normal adult human primary dermal fibroblasts (HDFa, ATCC PCS-201-012) were cultured in DMEM plus GlutaMax supplemented with 20% (v/v) FBS. All cell types were cultured at 37 °C with 5% CO_2_. Cell lines were authenticated by their suppliers and tested negative for mycoplasma.

#### HEK293T, HUH7 and U2OS cell line transfection protocols and genomic DNA isolation

HEK293T cells were seeded at a density of 2 × 10^4^ cells per well on 96-well plates (Corning) 16–20 h before transfection. Transfection conditions were as follows for HEK293T cells: 0.5 µl of Lipofectamine 2000 (Thermo Fisher Scientific), 250 ng of Cas effector plasmid (nuclease/base editor) and 83 ng of guide RNA plasmid were combined and diluted with Opti-MEM reduced serum media (Thermo Fisher Scientific) to a total volume of 10 µl and transfected according to the manufacturer’s protocol. Cells were transfected at approximately 60–80% confluency. HUH7 cells and U2OS cells were seeded at a density of 2.5 × 10^4^ cells per well on 96-well plates 16–20 h before transfection. Transfection conditions were as follows: 0.33 µl of Lipofectamine 2000, 112.5 ng of Cas effector plasmid and 37.5 ng of guide RNA plasmid were combined and diluted with Opti-MEM media to a total volume of 10 µl and transfected according to the manufacturer’s protocol. Cells were transfected at approximately 80–100% confluency. Following transfection, all cell types were cultured for 3 days, after which the media was removed, the cells washed with 1× PBS solution and genomic DNA harvested via cell lysis with 30 µl of lysis buffer added per well (10 mM Tris-HCL, pH 8.0, 0.05% SDS, 20 µg ml^−1^ Proteinase K (New England Biolabs)). The cell lysis mixture was allowed to incubate for 1–2 h at 37 °C before being transferred to 96-well PCR plates and enzyme inactivated for 30 min at 80 °C. The resulting genomic DNA mixture was stored at −20 °C until further use.

#### Base editor mRNA in vitro transcription

All base editor mRNA was generated from PCR product amplified from a template plasmid containing an expression vector for the base editor of interest cloned as described previously^59^. PCR product was amplified using forward primer IVT-F and reverse primer IVT-R (Supplementary Table 8), purified using the QIAquick PCR Purification Kit (Qiagen) and eluted in 15 µl of nuclease-free H_2_O. In vitro transcription was done using the HiScribe T7 High-Yield RNA Synthesis Kit (New England Biolabs) according to the manufacturer’s protocols but with full substitution of *N*1-methyl-pseudouridine (TriLink Biotechnologies) for uridine and cotranscriptional capping with CleanCap AG (TriLink Biotechnologies). mRNA isolation was performed using lithium chloride precipitation. Purified mRNA was stored at −20 °C until further use.

#### Human primary fibroblast nucleofection and genomic DNA extraction

One day before nucleofection, 80–90% confluent HDFa cells were passaged at a 1:2 dilution ratio into fresh media. Nucleofection was performed by pooling 1 × 10^5^ HDFa cells per condition and spun down at 300*g* for 10 min, washed with 1× PBS, spun again, then resuspended in P2 primary cell solution (10 µl per condition, Lonza). DNA mixtures were prepared by combining 50 pmol of chemically synthesized guide RNA^4^ (IDT or Synthego, Supplementary Table 9) with 1 µg of in vitro transcribed base editor mRNA and P2 primary cell solution into a total volume of 12 µl. For dose titration experiments, the amount of guide RNA was kept fixed, while the total amount of base editor mRNA was varied (125, 250 or 500 ng). Each 10 µl aliquot of HDFa cells is combined with DNA mixture to a total volume of 22 µl, and nucleofected with program DS-150 on 96-well Shuttle Device component of a 4D-Nucleofector system. Following nucleofection, cells were allowed to rest for 10 min before addition of 100 µl of prewarmed media per well. Next, 80 µl of each condition was subsequently taken and plated on a 48-well poly-d-lysine plate (Corning). Cells were cultured for 5 days postnucleofection, with media replacement after the first day. Following removal of media and a wash with 1× PBS buffer, genomic DNA was isolated by addition of 100 µl of lysis buffer following the same protocol as described for other cell lines. Genomic DNA was stored at −20 °C until further use.

#### High-throughput sequencing of genomic DNA

High-throughput sequencing of genomic DNA from all cell lines was performed as previously described^4^. Primers for PCR amplification of target genomic sites are listed in Supplementary Table 8, and the sequence identity of the target amplicons are listed in Supplementary Table 5. DNA concentrations were quantified with a Qubit double-stranded DNA High Sensitivity Assay Kit (Thermo Fisher Scientific) or with a NanoDrop One Spectrophotometer (Thermo Fisher Scientific) before sequencing on an Illumina MiSeq instrument (paired-end read R1 250–280 cycles, R2 0 cycles) according to the manufacturer’s protocols.

#### High-throughput sequencing data analysis

Individual sequencing runs were demultiplexed using the MiSeq Reporter (Illumina). Subsequent demultiplexed sequencing reads were analyzed using CRISPResso2 (ref. ^58^) as described previously^4^. All editing values are representative of *n* = 3 independent biological replicates, with mean ± s.e.m. shown.

#### In silico prediction of off-target sites

Off-target site prediction in silico was performed using CHOPCHOPv3 (ref. ^48^) and the ‘Paste Target’ functionality with the following parameters: the sites 1 and 2 20-nt SpRY protospacers and corresponding 3-nt PAMs were used as search queries; the Cas9 PAM was set to custom ‘NNN’ and mismatches within the protospacer set to 2; self-complementarity parameters were removed; all other parameters were left as default. Resulting off-targets were then screened manually, and sites with more than one mismatch within the PAM-proximal region (≤10 bp from the PAM) were removed.

### GUIDE-seq

#### U2OS nucleofection for GUIDE-seq

One day before nucleofection, 80–90% confluent U2OS cells were passaged at a 1:2 dilution ratio into fresh media. Nucleofection was performed by pooling 3 × 10^5^ U2OS cells per condition and spun down at 300*g* for 10 min, washed with 1× PBS, spun again, then resuspended in SE solution (10 µl per condition, Lonza). DNA mixtures were prepared by combining 750 ng of Cas9 plasmid, 250 ng of guide RNA plasmid, 5 pmol of the GUIDE-seq dsODN^49^ and SE solution into a total volume of 12 µ. Each 10 µl aliquot of U2OS cells is combined with DNA mixture to a total volume of 22 µl, and nucleofected with program DN-100 on the 96-well Shuttle Device component of a 4D-Nucleofector system. Following nucleofection, cells were allowed to rest for 10 min before addition of 100 µl of prewarmed media per well. Each condition was then split into two 50 µl aliquots and plated on 24-well plates (Corning). Cells were cultured for 5 days postnucleofection, with media replacement after the first day. Following removal of media and a wash with 1× PBS buffer, genomic DNA was isolated using the DNAdvance Genomic DNA Isolation Kit (Agencourt), following the manufacturer’s protocols. Genomic DNA was stored at −20 °C until further use.

#### Genomic DNA preparation and high-throughput sequencing for GUIDE-seq

Genomic DNA was prepared for GUIDE-seq as previously described^49^, with the following modifications. Genomic DNA shearing, end repair, dA-tailing and adapter ligation were done in a one-pot mixture using the NEBNext Ultra II FS DNA Library Prep Kit for Illumina (New England Biolabs), following the manufacturer’s protocol for input DNA >100 ng (without size selection) and a desired fragment size distribution between 300 and 700 bp. During the adapter ligation step, the manufacturer-suggested NEBNext Adapter for Illumina was replaced with the custom GUIDE-seq Y-adapter^49^. DNA purification was done with AMPure XP beads (Beckman Coulter). The subsequent PCR1, PCR2, library quantification, library normalization and high-throughput sequencing (paired-end Nextera sequencing: R1 150, I1 8, I2 8, R2 150) steps were done using the primers and protocols from the previously described protocol^49^.

#### GUIDE-seq analysis

Sequencing reads were demultiplexed using the MiSeq Reporter (Illumina), then processed individually using the GUIDE-seq analysis software (https://github.com/tsailabSJ/guideseq). SpRY variants were analyzed using a mismatch threshold of eight and an NNN PAM. Nme2Cas9 variants were analyzed using a mismatch threshold of 11 and an NNNNNN PAM. Background reads and associated genomic loci from the dsODN-only treated sample are listed in Supplementary Table 3. Visualization plots in Extended Data Fig. 8 were generated using a custom version of the original script, which has been uploaded to the Khalil Lab GitHub repository (https://github.com/khalillab/guideseq).

### Reporting summary

Further information on research design is available in the Nature Research Reporting Summary linked to this article.

## Online content

Any methods, additional references, Nature Research reporting summaries, source data, extended data, supplementary information, acknowledgements, peer review information; details of author contributions and competing interests; and statements of data and code availability are available at 10.1038/s41587-022-01410-2.

## Supplementary information

Supplementary Information Supplementary Figs. 1–23, Tables 1–9 (or placeholders) and Notes 1–8.

Reporting Summary

Supplementary Tables 1, 3, 5 Compiled BE-PPA library data used to characterize the PAM profile of Cas9 variants; compiled GUIDE-seq analysis; list of target sites.

## Data Availability

High-throughput DNA sequencing FASTQ files are available from the NCBI SRA under BioProject PRJNA853525 (ref. ^60^). Other data, including mammalian cell data analysis (PRISM files) or phage titer analysis from selection development and ePACE evolutions 1–5, are available from the corresponding authors upon request. Plasmids encoding select SAC-PACE components and evolved Nme2Cas9 genome editing agents have been deposited at Addgene for distribution.

## Extended data

are available for this paper at 10.1038/s41587-022-01410-2.

## Code availability

Data analysis python code and eVOLVER experiment code is provided at (https://github.com/khalillab/ePACE-Nme2Cas9-analysis)^61^. Modified GUIDE-seq analysis code has uploaded to the Khalil Lab GitHub repository (https://github.com/khalillab/guideseq)^62^.
