# Supplementary material for: High-throughput continuous evolution of compact Cas9 variants targeting single-nucleotide-pyrimidine PAMs
Source: Nat Biotechnol. 2022 Sep 8;41(1):96–107. doi: 10.1038/s41587-022-01410-2 (PMC9849140; doi:10.1038/s41587-022-01410-2)
Supplement: Supplementary file 1 — Supplementary Figs. 1–23, Tables 1–9 (or placeholders) and Notes 1–8. [file 41587_2022_1410_MOESM1_ESM.pdf]

nature portfolio

https://doi.org/10.1038/s41587-022-01410-2

Supplementary information

High-throughput continuous evolution of compact Cas9 variants targeting single-nucleotide-pyrimidine PAMs

In the format provided by the authors and unedited

Supplementary Information

High-throughput continuous evolution of compact Cas9 variants targeting single-nucleotide-pyrimidine PAMs

Tony P. Huang[1,2,3,8], Zachary J. Heins[4,5,8], Shannon M. Miller[1,2,3], Brandon G. Wong[4,5], Pallavi A. Balivada[4,5], Tina Wang[1,2,3,7], Ahmad S. Khalil[4,5,6*] and David R. Liu[1,2,3,*]

[1]Merkin Institute of Transformative Technologies in Healthcare, The Broad Institute of Harvard and MIT, Cambridge, MA, USA
[2]Department of Chemistry and Chemical Biology, Harvard University, Cambridge, MA, USA
[3]Howard Hughes Medical Institute, Harvard University, Cambridge, MA, USA
[4]Biological Design Center, Boston University, Boston, MA, USA
[5]Department of Biomedical Engineering, Boston University, Boston, MA, USA
[6]Wyss Institute for Biologically Inspired Engineering, Harvard University, Boston, MA, USA
[7]Department of Chemistry, University of Wisconsin-Madison, Madison, WI, USA
[8]These authors contributed equally

*Corresponding authors

Supplementary Figures

1. High level description of ePACE components.
2. Integrated Peristaltic Pump (IPP) characterization.
3. eVOLVER pressure regulator characterization.
4. ePACE validation on two-hybrid Maltose Binding Protein (MBP).
5. Flow rate schedule and titers for ePACE1.
6. Mutation table and representative activity of ePACE1 evolved Nme2Cas9 variants.
7. Flow rate schedule and titers for ePACE2.
8. Identification of ePACE2 selection cheating.
9. Source gels for Supplementary Figure 8
10. Mutation table and representative activity of ePACE2 evolved Nme2Cas9 variants.
11. Validation of the split SAC-PACE selection with different TadABE8e variants.
12. Flow rate schedule and titers for ePACE3.
13. Mutation table and representative activity of ePACE3 evolved Nme2Cas9 variants.
14. PANCE dilution schedule and titers for N1.
15. Flow rate schedule and titers for ePACE4.
16. PANCE dilution schedule and titers for N2.
17. Flow rate schedule and titers for ePACE5.
18. eNme2-T.1-ABE8e and eNme2-T.2-ABE8e activity at N$_4$VN PAM sites.
19. *In silico* prediction of off-target sites with ≤ 3 mismatches for a 20-nt or 23-nt protospacer.
20. High-throughput sequencing validation of GUIDE-seq-identified off-target activity.
21. Off-target adenine base editing at *in silico*-predicted off-target sites for SpRY-ABE8e, SpRY-HF1-ABE8e, eNme2-T.1-ABE8e and eNme2-T.2-ABE8e.
22. Dose-dependent adenine base editing activity in primary human dermal fibroblasts.
23. Off-target adenine base editing at *in silico*-predicted off-target sites for SpCas9-NRCH and eNme2-C sgRNAs targeting the *HBB* sickle-cell disease mutation.

Supplementary Tables

1. BE-PPA library editing data.
2. CHOPCHOPv3-identified off-target sites of protospacer-matched sgRNAs comparing eNme2-C to SpRY and SpRY-HF1.
3. GUIDE-Seq identified off-target sites.
4. CHOPCHOPv3-identified off-target sites of protospacer-matched sgRNAs comparing eNme2-T.1 and eNme2-T.2 to SpRY and SpRY-HF1.
5. List of target sites.
6. CHOPCHOPv3-identified off-target sites of sgRNAs targeting the *HBB* sickle-cell disease mutation.
7. Plasmids and selection phage (SP) used in this work.
8. Primers used in this work.
9. Chemically-synthesized guide RNAs used for HDFa cells.

Supplementary Notes

1. ePACE pressure regulation.
2. ePACE2 recombination and cheating.
3. Validation of the split base editor SAC-PACE selection.
4. PAM-specific activity of ePACE4 evolved variants observed in ABE-PPA.
5. Reversion analysis of eNme2-C RuvC/HNH domain mutations.
6. Analysis and limitations of BE-PPA for evaluating Nme2Cas9 PAM compatibility.
7. Design error for the N_4CN trajectory dual PAM split SAC-PACE APs.
8. Evolved Nme2Cas9 amino acid sequences.

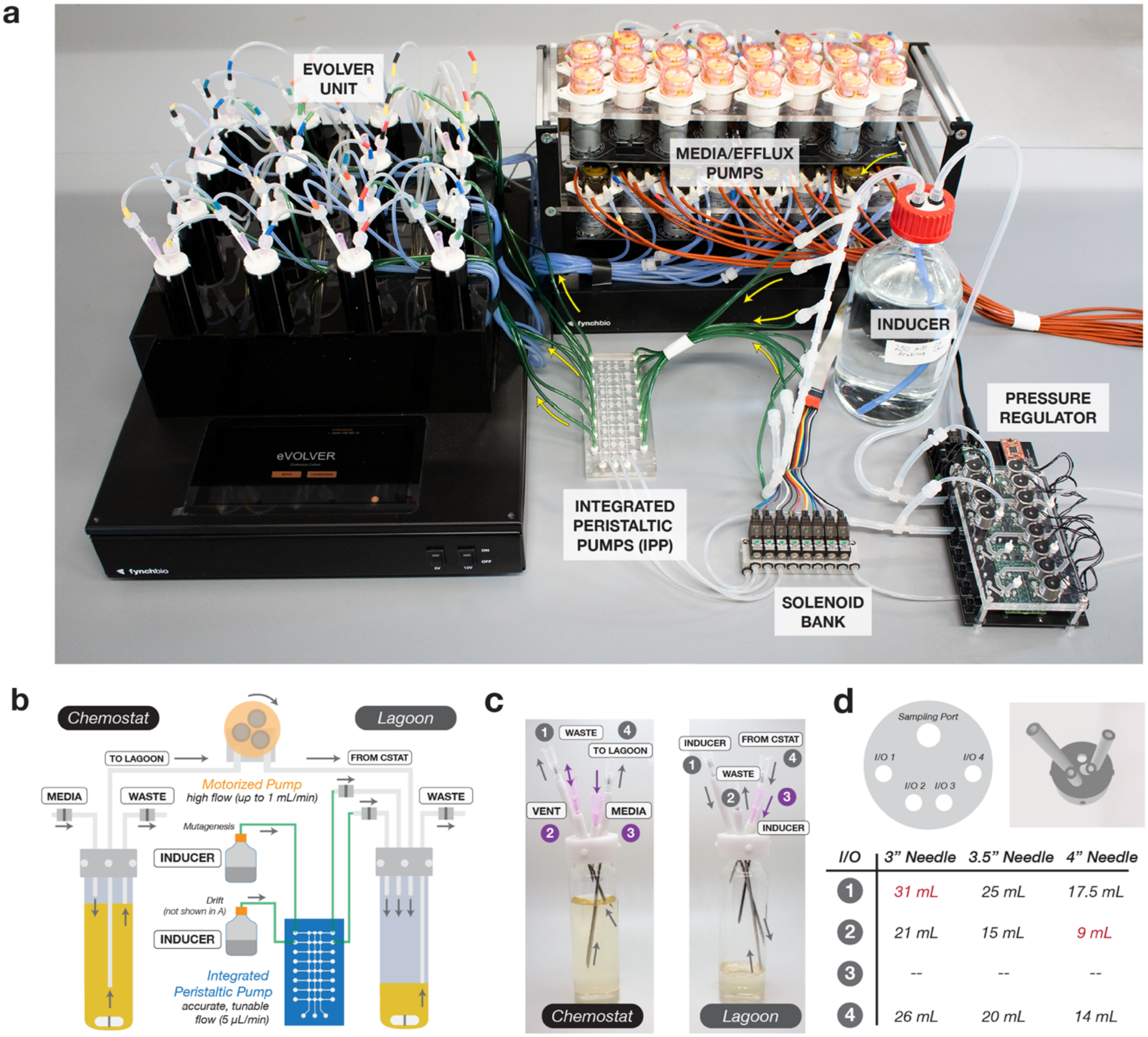

Supplementary Figure 1. High level description of ePACE components. (a) Photograph of ePACE, consisting of an eVOLVER continuous culture unit with custom vial caps, fluidics unit with a set of slow (~1 ml/m, pink pump heads) and fast (~1 ml/s, black pump heads) pump arrays for vial-to-vial/media pumping and waste pumping respectively, IPP device for chemical inducer pumping (~0.5 ul/s), and a multi-channel pressure regulator for powering IPP devices and pressurizing inducer bottles. **(b)** Diagram of fluidics for a single ePACE chemostat/lagoon pair. **(c)** Photograph of custom vials and caps designed for ePACE, labeled for a typical setup. Caps are designed to be used with hypodermic needles, but can also be used with other types of tubing if desired. **(d)** Diagram of volume levels for each input/output (I/O) port on the caps with different length needles. In ePACE, the efflux needle is set to 31 ml and 9 ml for the chemostat and lagoon, respectively (highlighted in red).

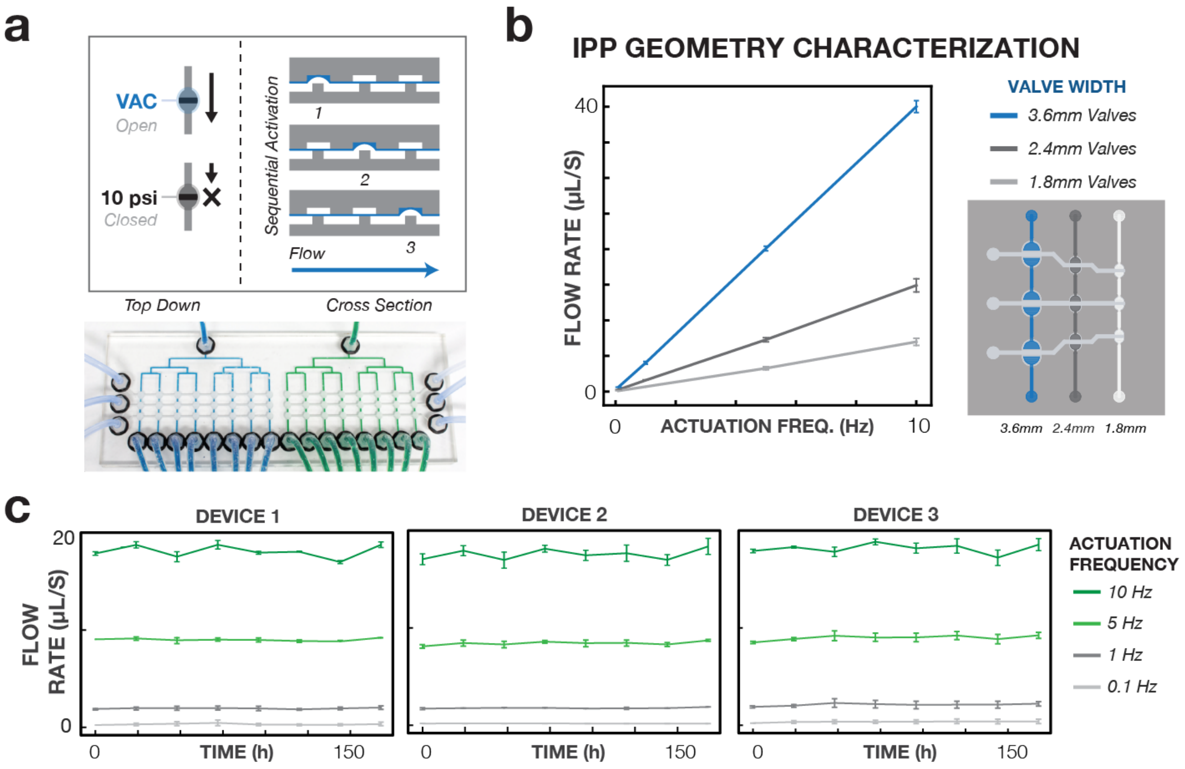

Supplementary Figure 2. Integrated Peristaltic Pump (IPP) characterization.
(a) Diagram of IPP functionality. Three valves in series are sequentially opened and closed to induce a peristaltic effect on the flow line. A single set of control lines can be used to pump many channels in parallel. **(b)** Valve geometry effects on achievable flow rates. Error bars represent the standard deviation over three measurements on a single channel of a single device. **(c)** Three IPP devices, each with three parallel channels with linked control lines, were run continuously for 168 hours at 10 Hz. Every 24 hours, the devices were briefly stopped and flow rate measurements were taken across the device performance range at 10 Hz, 5 Hz, 1 Hz, and 0.1 Hz. Devices were then restarted at 10 Hz immediately after measurements were taken. For **b** and **c**, data are presented as mean±SD of *n*=3 independent technical replicates.

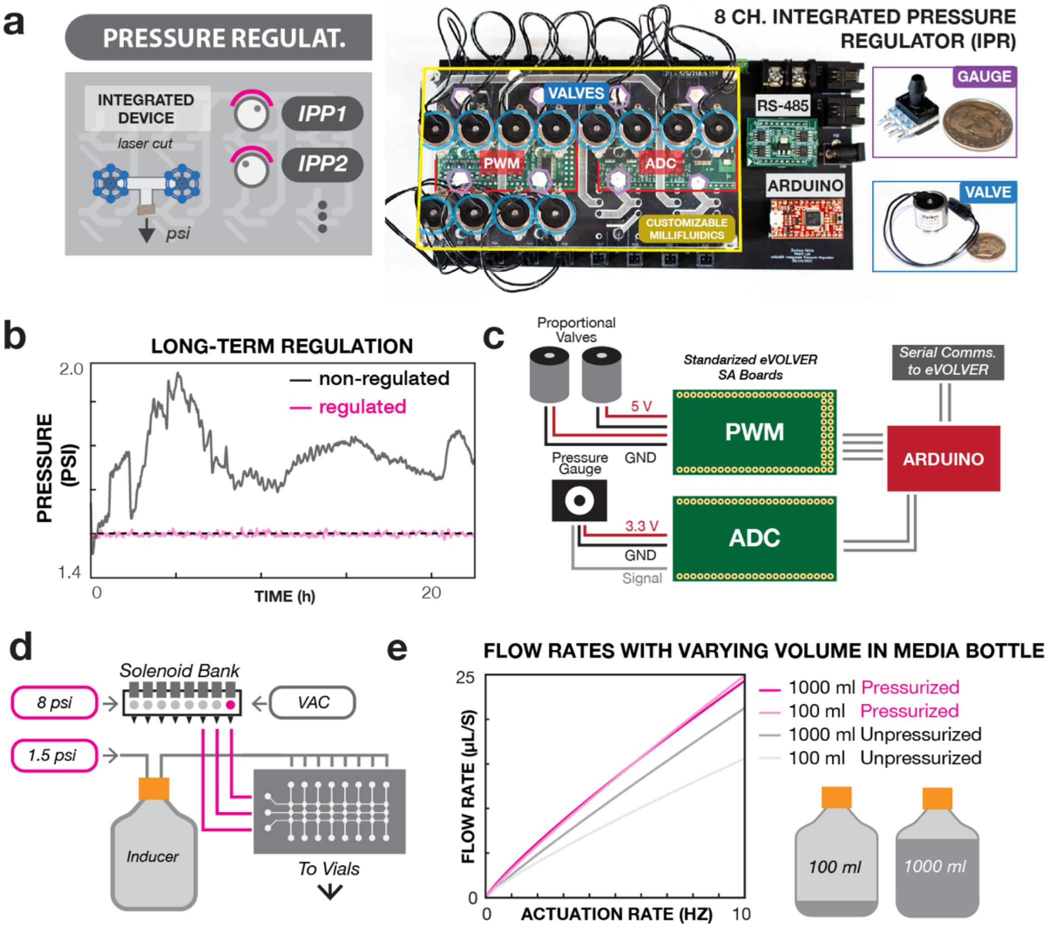

Supplementary Figure 3. eVOLVER pressure regulator characterization. (a) Diagram and photo of an 8-channel PID controlled pressure regulator. **(b)** Comparison of pressures over 24 hours of PID controlled pressure to a manually set valve, both initial set at 1.5 psi. **(c)** Simplified electrical schematic of eVOLVER pressure regulator. Each proportional valve is controlled via pulse-width modulation (PWM) using a standard eVOLVER PWM board. A single PWM board can control 16 valves simultaneously, enabling control of eight individual pressure lines. Electrical pressure gauge readouts are connected to a standard eVOLVER analog-to-digital (ADC) converter. Both PWM and ADC boards are connected to a SAMD21 Arduino microcontroller which controls valve open/closeness and reads data from the gauges. The microcontroller receives commands from and sends data to the eVOLVER via serial communication protocol. **(d)** Schematic of pressure regulation for ePACE. The IPP devices are powered by 8 psi provided by the pressure regulator and standard lab bench vacuum. Inducer bottles receive 1.5 psi. **(e)** Comparison of flow rates between media bottles with varying volumes of media while pressurized and un-pressurized.

a

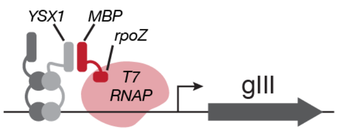

b

WT MBP

residue number

	52	63	85	182	233
MBP	A	A	F	G	S
V6.1	V	A	F	G	S
V6.2	A	A	F	G	S
V6.3	A	A	L	G	S
V6.4	A	S	F	G	S
V6.5	A	A	F	G	P
V6.6	A	A	F	D	S

MBP G32D+I33S

residue number

	32	63	66	67	99	211	260
G32D/ I33S	D	A	R	F	Y	S	G
V6.1	D	T	L	F	Y	S	G
V6.2	D	A	R	F	Y	S	V
V6.3	D	A	R	F	Y	S	V
V6.4	G	T	R	F	Y	I	G
V6.5	G	T	R	F	Y	I	G
V6.6	D	A	R	F	Y	S	V
V6.7	G	T	R	F	Y	I	G
V6.8	D	A	R	L	H	S	G

Supplementary Figure 4. ePACE validation on two-hybrid Maltose Binding Protein (MBP) selection. **(a)** Diagram of two-hybrid MBP selection. Upon proper folding of MBP, a T7 RNA Polymerase is recruited to transcribe gIII. **(b)** Mutation tables of negative control WT MBP and structurally defective MBP after 120 hours of ePACE. MBP G32+I33S shows converging mutations at residues clustered around the monobody-MBP interaction interface (D32G, A63T, R66L), previously observed in PACE[1].

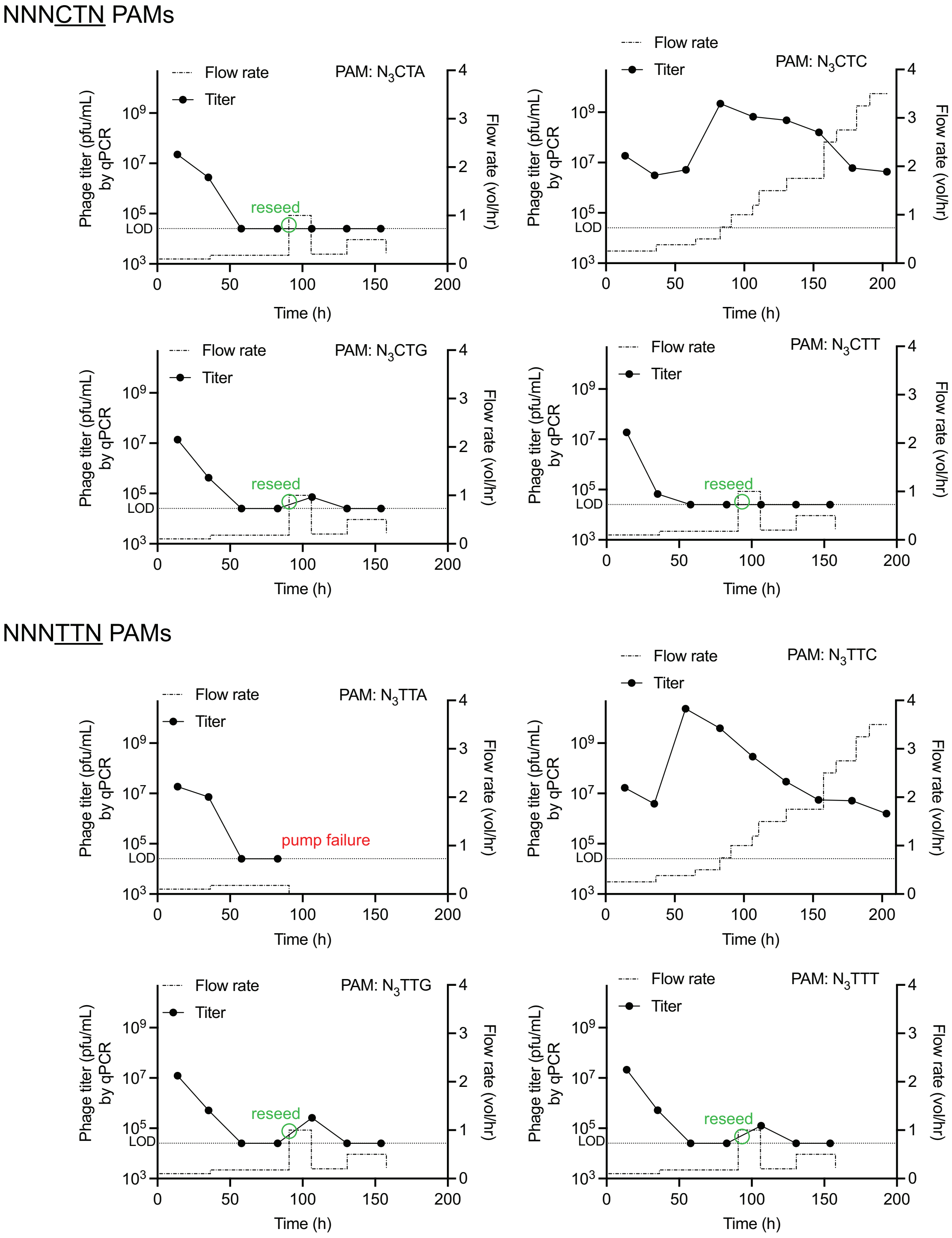

Supplementary Figure 5. Flow rate schedule and titers for ePACE1. SP containing wild-type, full-length Nme2ABE8e were first diversified in *E.coli* host cells containing pJC175e[2] and MP6[2], isolated, then seeded into ePACE1 (eight chemostats, one lagoon each targeting each of the eight N_3<u>YTN</u> PAMs). Flow rate stringency for each PAM is shown in the plots, as are resulting titers (measured by qPCR). If lagoons were reseeded with starting phage, the timepoint is highlighted in a green circle. The N_3<u>TTA</u> lagoon failed prematurely due to a pump failure in the ePACE setup. LOD=limit of detection of qPCR titering, as set by the titer corresponding to the C_q for which the qPCR primers alone had been observed to amplify.

a

	TadA8e	dNme2Cas9 (N-terminal: REC/HNH/RuvC)																				
Residue number	26	47	60	67	105	212	296	335	336	337	441	451	462	560	581	616	647	657	768	777	805	808	811
wild-type	R	E	M	S	S	L	P	L	R	Y	Y	D	I	E	K	N	S	F	Q	E	K	T	A
CTC.E1-1	G					P						V					N						
CTC.E1-2	G										C				R							I	
CTC.E1-3	G										C				R					G			
CTC.E1-4	G										C				R								
TTC.E1-5					G			P	K				V						R				
TTC.E1-6	G			R			L			C						S							T
TTC.E1-7		K	V									E	V	G				V		G	R		
TTC.E1-8	G			R												S							

	dNme2Cas9 (C-terminal: WED, PID)									
Residue number	844	904	908	1025	1033	1035	1043	1045	1051	1075
wild-type	D	K	D	I	R	Y	S	E	S	L
CTC.E1-1	V			S	K		R			
CTC.E1-2	G			S	K		R			M
CTC.E1-3	G			S	K		R			M
CTC.E1-4	G			S	K		R			M
TTC.E1-5	V					C		A	P	
TTC.E1-6	V	N				H			A	
TTC.E1-7	V		G			H				
TTC.E1-8	V	N				H				

b

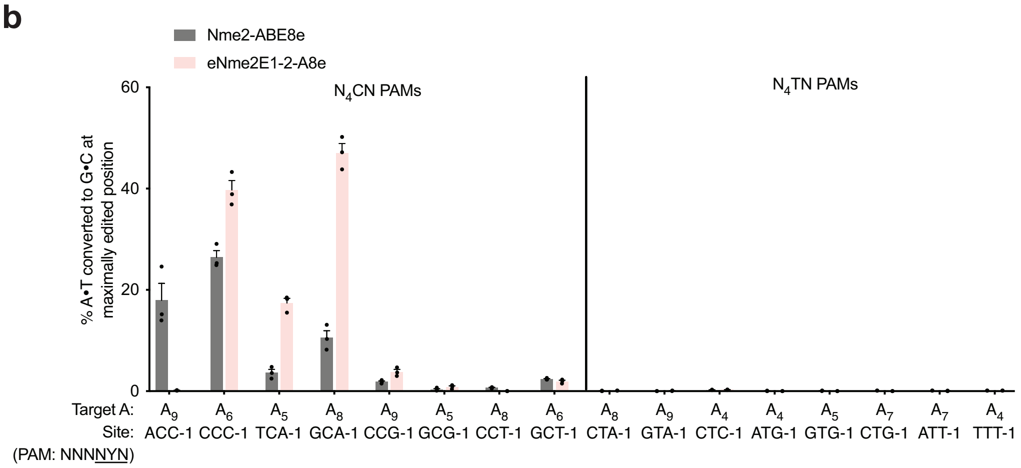

Supplementary Figure 6. Mutation table and representative activity of ePACE1 evolved Nme2Cas9 variants. (a) Genotypes of individually sequenced plaques following ePACE1, with positions varying from wild-type displayed. Clones evolved on different PAMs are delineated by a bold line. **(b)** Adenine base editing activity of a representative ePACE1 clone (E1-2-ABE8e) at eight N_3NCN PAM-containing sites and eight N_4TN PAM-containing sites in HEK293T cells. Mean±SEM are shown and are representative of *n*=3 independent biological replicates.

NNNCTN PAMs

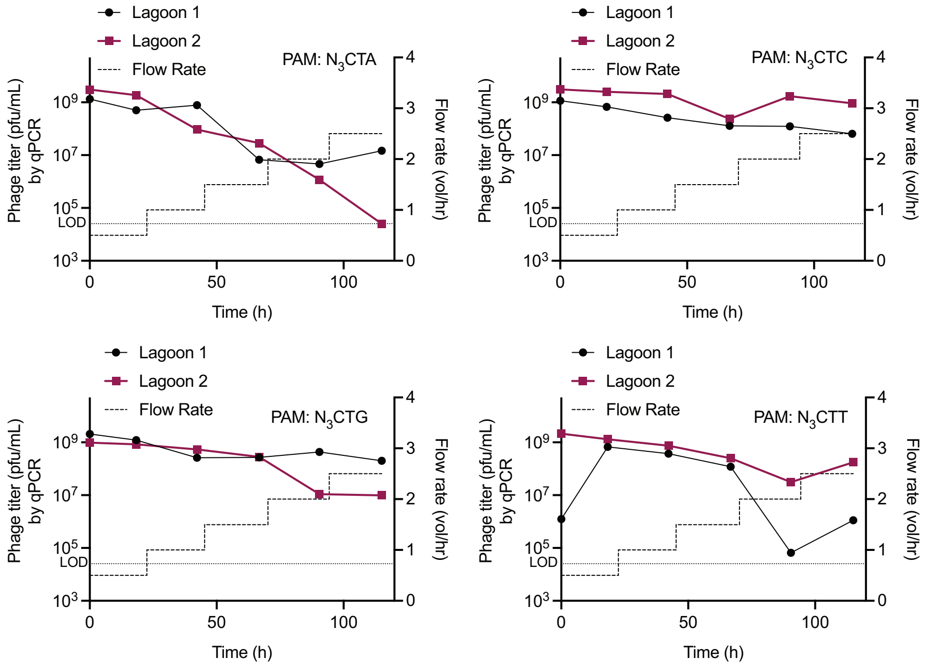

NNNTTN PAMs

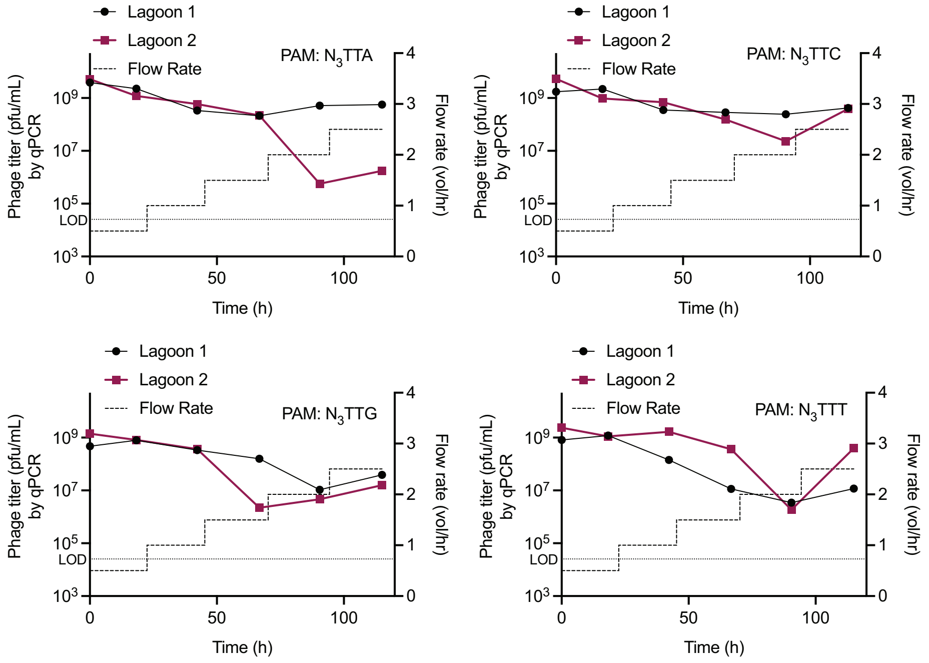

Supplementary Figure 7. Flow rate schedule and titers for ePACE2. SP previously isolated from ePACE1 lagoons evolved on N_3TTC and N_3CTC PAMs were pooled and reseeded into ePACE2 (eight chemostats, two lagoons each targeting each of the eight N_3YTN PAMs). Flow rate stringency for each PAM is shown in the plots, as are resulting titers (measured by qPCR). LOD=limit of detection of qPCR titering, as set by the titer corresponding to the C_q for which the qPCR primers alone had been observed to amplify.

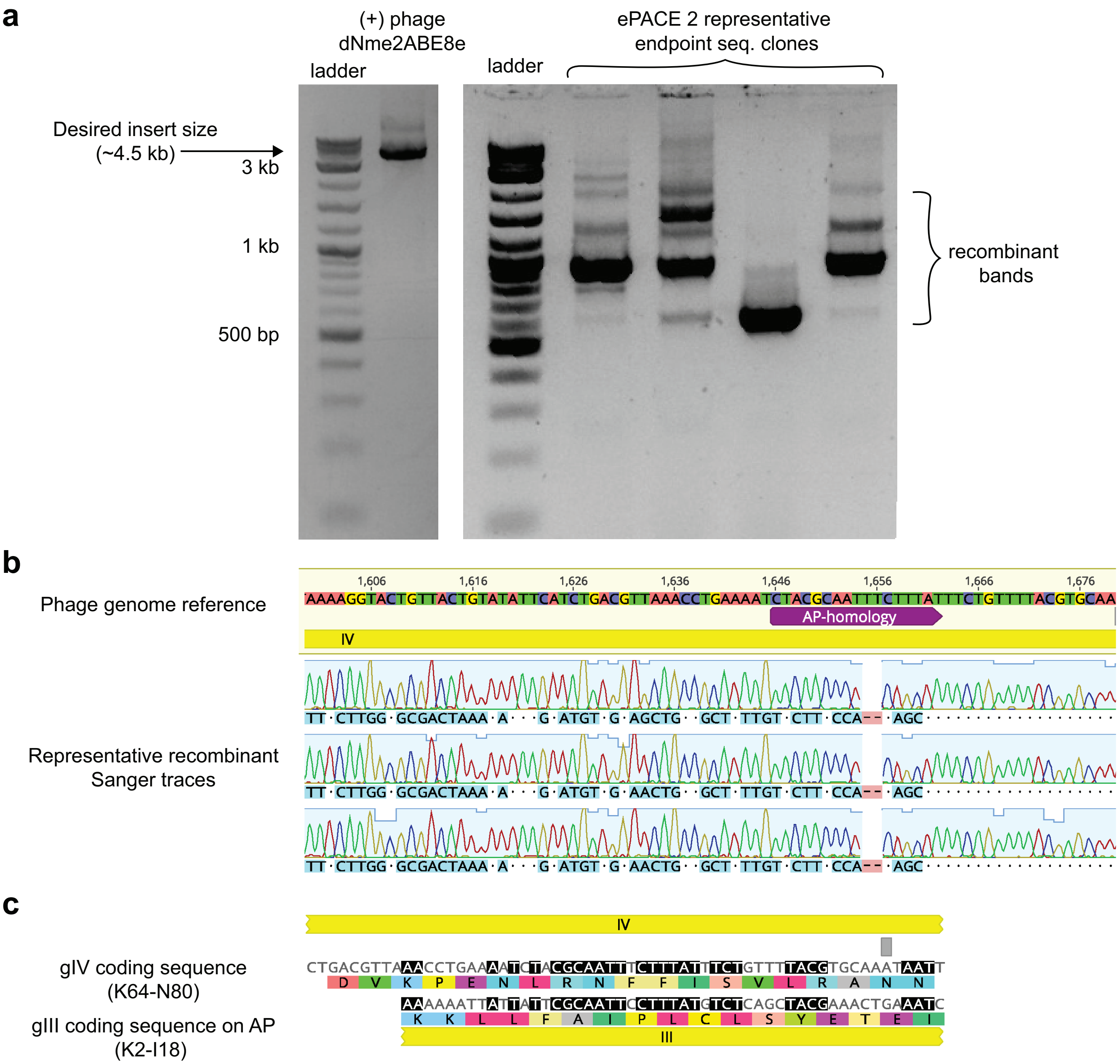

Supplementary Figure 8. Identification of ePACE2 selection cheating. (a) Representative agarose gel of PCR products amplifying the target insert from individual ePACE2 late timepoint SP plaques. The expected insert size for Nme2ABE8e is ~4.5 kb (left, starting SP), whereas multiple recombinant bands appeared for ePACE2 evolved SP (right). The gels were not repeated (*n*=1). **(b)** Sanger sequencing partially mapping the recombinant bands from (a) onto the gVI coding sequence, the unaligned sequence to the left maps to the gIII-containing AP sequence. **(c)** Nucleotide sequence homology between the coding sequence of gIV (where recombination was seen) and the gIII coding sequence present on the AP, aligned nucleotides highlighted in black.

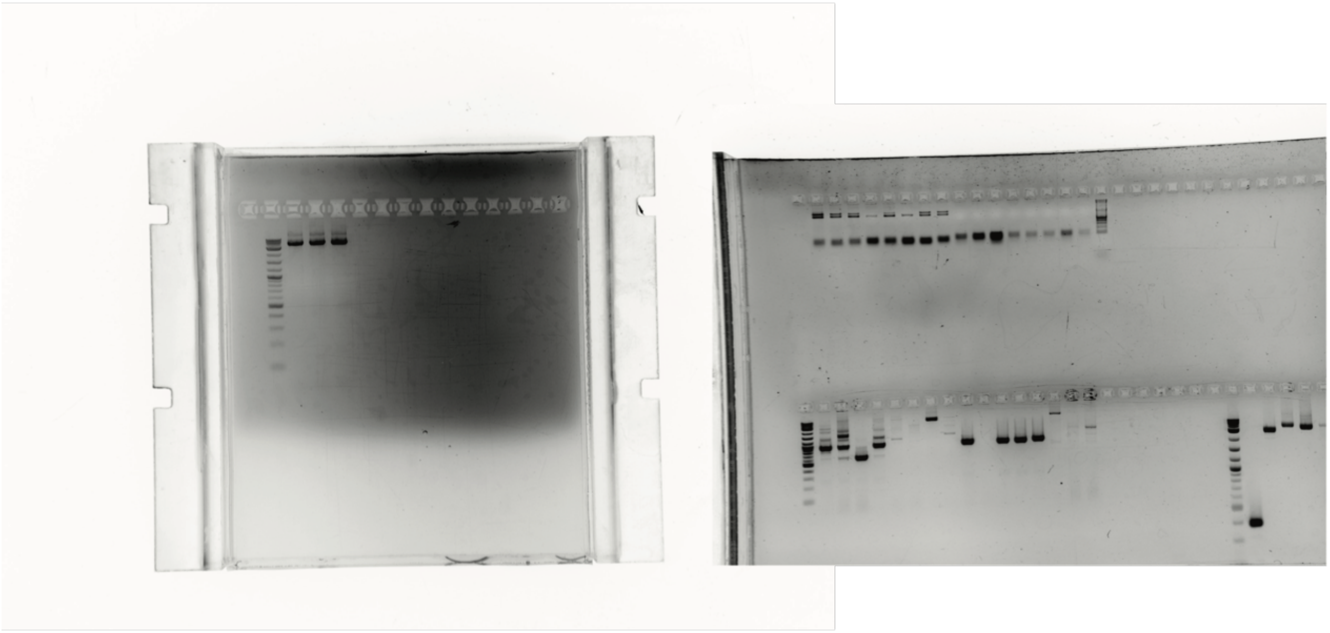

Supplementary Figure 9. Source gels for Supplementary Figure 8. Uncropped gels that were used to generate Supplementary Figure 8a. The two gels were taken at separate times, but contain the same ladder for reference. The right gel wase cropped to remove lanes from unrelated experiments.

a

	TadA8e								dNme2Cas9 (N-terminal: REC/HNH/RuvC)																
Residue number	26	49	53	71	102	124	156	163	2	47	53	68	94	119	123	154	186	323	340	361	396	409	424	431	441
wild-type	R	A	H	M	R	Y	V	A	A	E	K	V	A	D	T	E	E	L	D	E	T	E	S	I	Y
CTC-L1.E2-1	G						A		S				E										N		C
CTC-L1.E2-2	G																								C
CTC-L1.E2-3	G																								C
CTC-L1.E2-4	G																								C
CTC-L2.E2-5	G							T			R														C
CTC-L2.E2-6	G																								C
CTC-L2.E2-7	G																								C
CTC-L2.E2-8	G																								C
CTG-L2.E2-9	G																							F	C
CTG-L2.E2-10	G																D							F	C
CTG-L2.E2-11																	D							F	C
CTT-L2.E2-12	G				H					K		M			A	K				A	A				
CTT-L2.E2-13	G		P	I		H				K		M		G	A	K					A				
CTT-L2.E2-14	G					H				K		M			A	K					A				
CTT-L2.E2-15	G									K		M			A	K					A				
TTG-L2.E2-16		V								K		M				K		R			A				
TTG-L2.E2-17	G									K		M				K									
TTT-L1.E2-18	G									K						K			A						
TTT-L1.E2-19	G									K						K			A			K			
TTT-L1.E2-20	G									K						K						K			

	dNme2Cas9 (N-terminal: REC/HNH/RuvC)																							
Residue number	451	452	462	508	520	532	536	545	581	624	633	634	659	701	719	720	762	767	768	769	770	771	792	813
wild-type	D	H	I	E	E	K	K	V	K	N	Q	E	E	Q	N	D	T	H	Q	K	T	H	G	K
CTC-L1.E2-1			V																R	R				R
CTC-L1.E2-2			V																	R				R
CTC-L1.E2-3			V																	R				R
CTC-L1.E2-4			V			T														R		R		R
CTC-L2.E2-5			V										D							R		R		R
CTC-L2.E2-6			V																	R		R		R
CTC-L2.E2-7			V																					R
CTC-L2.E2-8			V																	R		R		R
CTG-L2.E2-9									R								A				A			
CTG-L2.E2-10									R						S		A	R			A		V	R
CTG-L2.E2-11				D					R			K					A	R			A		V	R
CTT-L2.E2-12		R														A		Y	R			L		R
CTT-L2.E2-13		R					R			D						A								
CTT-L2.E2-14		R														A								
CTT-L2.E2-15		R														A			R					
TTG-L2.E2-16		R						M						R		A								
TTG-L2.E2-17	V										R					A								
TTT-L1.E2-18	N	R			K																			
TTT-L1.E2-19	N	R			K																			
TTT-L1.E2-20	N	R			K																			

	dNme2Cas9 (C-terminal: WED, PID)																			
Residue number	844	858	869	911	929	932	933	951	968	981	986	991	1005	1025	1028	1033	1043	1045	1049	1075
wild-type	D	K	I	D	K	E	S	M	Y	N	I	Y	K	I	D	R	S	E	R	L
CTC-L1.E2-1	V		T											S		K	R			M
CTC-L1.E2-2	V													S		K	R			M
CTC-L1.E2-3	V													S		K	R			
CTC-L1.E2-4	V	R							H					S		K	R			
CTC-L2.E2-5	V													S		K	R			
CTC-L2.E2-6	V													S		K	R			
CTC-L2.E2-7	V													S		K	R			
CTC-L2.E2-8	V													S		K	R			
CTG-L2.E2-9	V													S	N	K	R		C	
CTG-L2.E2-10	V			A	R		R							S	N	K	R		C	
CTG-L2.E2-11	V			A	R		R							S	N	K	R		C	
CTT-L2.E2-12	A							R								K				
CTT-L2.E2-13	A					G		R				H				K		A		
CTT-L2.E2-14	A							R								K				
CTT-L2.E2-15	A							R								K				M
TTG-L2.E2-16	A							R		S						K				
TTG-L2.E2-17	A							R								K	R			
TTT-L1.E2-18	A							R			T		R			K	R			
TTT-L1.E2-19	A							R					R			K	R			
TTT-L1.E2-20	A							R					R			K	R	D		

b

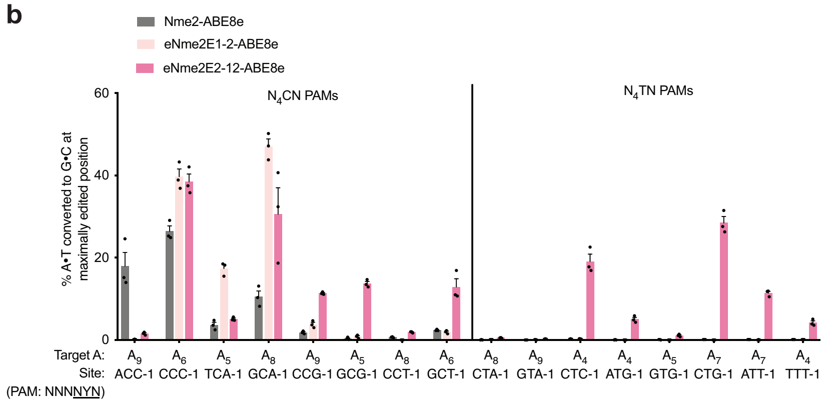

Supplementary Figure 10. Mutation table and representative activity of ePACE2 evolved Nme2Cas9 variants. (a) Genotypes of individually sequenced plaques following

ePACE2, with positions varying from wild-type displayed. Clones evolved on different PAMs are delineated by a bold line. Mutations that had previously appeared in ePACE1 are shown in light pink, while novel mutations are shown in magenta. **(b)** Adenine base editing activity of a representative ePACE2 clone (E2-12-ABE8e) at eight N_4CN PAM-containing sites and eight N_4TN PAM-containing sites in HEK293T cells. Mean±SEM are shown and are representative of *n*=3 independent biological replicates.

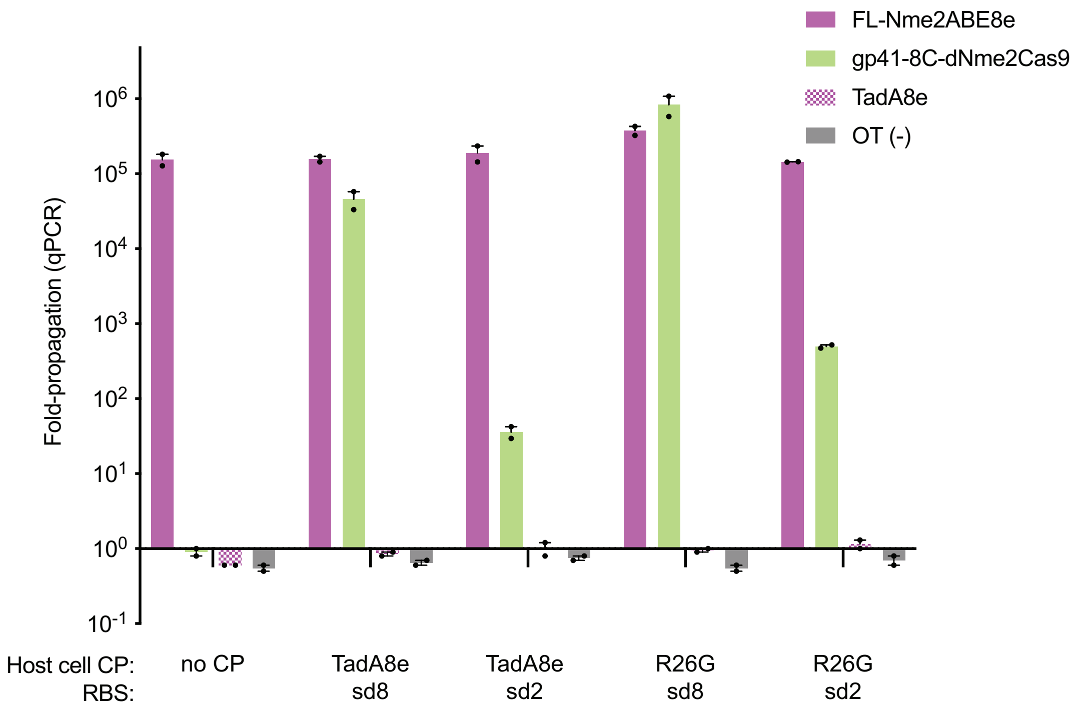

Supplementary Figure 11. Validation of the split-SAC-PACE selection with different TadABE8e variants. Overnight propagation assay to test the activity of the split-SAC-PACE selection with different TadA8e variants. Each TadA8e variant was fused to the N-terminal half of an intein (gp41-8N) and placed on a complementary plasmid (CP) in host cells. FL-Nme2ABE8e phage contained full-length, active Nme2ABE8e, and OT phage did not contain Nme2Cas9, intein, or TadA8e. Mean±SEM are shown and are representative of n=2 independent biological replicates. Fold-propagation is calculated as the ratio of phage titer after overnight propagation over inoculating titer.

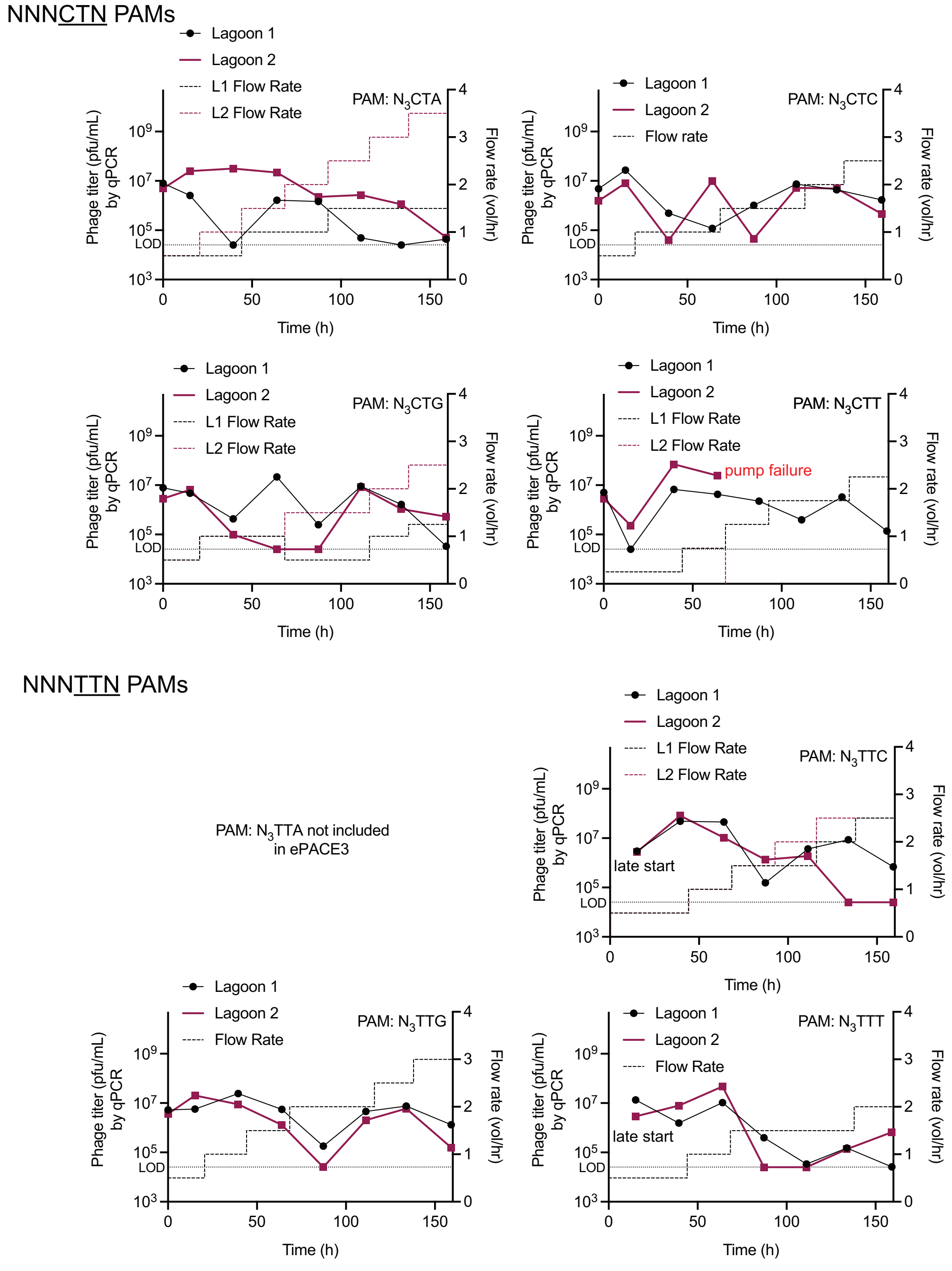

Supplementary Figure 12. Flow rate schedule and titers for ePACE3. SP from ePACE1 sequenced SP from ePACE2 were pooled and recloned into the split-SAC-PACE phage architecture (SP404, **Supplementary Table 7**), then seeded into ePACE3 (seven chemostats, two lagoons each targeting each of the eight N_3YTN PAMs; N_3TTA was excluded due to a cloning error). Flow rate stringency for each PAM is shown in the plots, as are resulting titers (measured by qPCR). The N_3TTC and N_3TTT lagoons were started late due to slow initial host cell growth. LOD=limit of detection of qPCR titering, as set by the titer corresponding to the C_q for which the qPCR primers alone had been observed to amplify.

a

dNme2Cas9 (N-terminal: REC/HNH/RuvC)

Residue number	10	47	68	76	108	123	149	152	154	158	207	210	338	344	361	396	401	452	470	546	555	598	660	674	691	720	750	762	767	770	816
wild-type	N	E	V	A	N	T	E	D	E	L	D	A	G	A	E	T	D	H	D	G	K	D	D	N	G	D	M	T	H	T	S
CTA-L1.E3-1		K	M			A			K							A		R	N	S		Y				A					
CTA-L1.E3-2		K	M			A			K							A		R	N	S		Y				A					
CTA-L1.E3-3		K	M			A			K							A		R	N	S		Y				A					
CTA-L2.E3-4		K	M			A			K							A		R							D	A					
CTA-L2.E3-5		K	M			A			R							A		R								A					
CTA-L2.E3-6		K				A			K							A		R								A					
CTC-L1.E3-7		K	M			A			K							A		R								A		A	Y		
CTC-L1.E3-8		K	M			A			K							A		R								A		A	Y		
CTC-L1.E3-9		K	M			A	A		K							A		R								A		A	Y		
CTC-L1.E3-10		K	M			A	A		K							A		R								A		A	Y		
CTC-L2.E3-11		K				A			K							A		R													
CTG-L1.E3-12		K			H				K		N	S	V	V				R					G					A	Y	A	
CTG-L1.E3-13		K			H				K		N	S	V	V				R					G					A	Y		
CTG-L1.E3-14		K			H				K		N	S	V	V				R					G					A	Y		
CTG-L1.E3-15		K			H				K		N	S	V	V				R					G					A	Y	A	
CTG-L2.E3-16		K	M			A			K							A		R								A		A	Y		
CTG-L2.E3-17		K	M			A			K							A		R								A		A	Y		
CTT-L1.E3-18		K	M			A			K			S				A	N	C				Y				A	T				
CTT-L2.E3-19		K		V		A		G	K							A		R						S		A					
CTT-L2.E3-20		K		V		A		G	K							A		R						S		A					
CTT-L2.E3-21		K	M			A			K							A		R								A					
CTT-L2.E3-22		K	M			A		N	K							A		R						S		A					
TTC-L2.E3-23		K	M			A			K							A		R								A		A	Y		
TTC-L2.E3-24		K	M			A			K							A		R								A		A	Y		
TTC-L2.E3-25		K	M			A	A		K							A		R								A		A	Y		
TTC-L2.E3-26		K	M			A	A		K							A		R								A		A	Y		
TTG-L1.E3-27		K				A			K							A		R								A					
TTG-L1.E3-28		K	M			A			K							A		R								A		A	Y		
TTG-L1.E3-29		K	M			A			K							A		R								A		A	Y		
TTG-L1.E3-30		K	M			A			K							A		R								A		A	Y		
TTG-L2.E3-31		K	M			A			K	M						A		R								A		A	Y		
TTG-L2.E3-32		K	M			A			K							A		R								A		A	Y		
TTG-L2.E3-33		K	M			A			K	M						A		R								A		A	Y		
TTT-L2.E3-34		K				A			K						K	A		R								A					
TTT-L2.E3-35		K				A			K						K	A		R								A					
TTT-L2.E3-36	S	K	M		H	A			K							A		R			R					A					I
TTT-L2.E3-37	S	K	M		H	A			K							A		R			R					A					I

dNme2Cas9 (C-terminal: WED, PID)

Residue number	844	894	911	929	933	951	1005	1025	1028	1029	1033	1043	1077
wild-type	D	A	D	K	S	M	K	I	D	S	R	S	K
CTA-L1.E3-1	A					R					K		
CTA-L1.E3-2	A					R					K		
CTA-L1.E3-3	A					R					K		
CTA-L2.E3-4	A					R					K		N
CTA-L2.E3-5	A					R					K		
CTA-L2.E3-6	A					R					K		
CTC-L1.E3-7	A					R					K		
CTC-L1.E3-8	A					R					K		
CTC-L1.E3-9	A					R					K		
CTC-L1.E3-10	A					R					K		
CTC-L2.E3-11	A					R					K		
CTG-L1.E3-12	A			R	R			S	N		K	R	
CTG-L1.E3-13	A				R			N	N		K	R	
CTG-L1.E3-14	A			R	R			S	N		K	R	
CTG-L1.E3-15	A			R	R			S	N		K	R	
CTG-L2.E3-16	A					R					K		
CTG-L2.E3-17	A					R					K		
CTT-L1.E3-18	A	V				R					K		
CTT-L2.E3-19	A					R	R			A	K		
CTT-L2.E3-20	A					R	R			A	K		
CTT-L2.E3-21	A					R					K		
CTT-L2.E3-22	A					R				A	K		
TTC-L2.E3-23	A					R					K		
TTC-L2.E3-24	A					R	R				K		
TTC-L2.E3-25	A					R					K		
TTC-L2.E3-26	A					R					K		
TTG-L1.E3-27	A					R					K		
TTG-L1.E3-28	A					R					K		
TTG-L1.E3-29	A					R					K		
TTG-L1.E3-30	A					R					K		
TTG-L2.E3-31	A					R					K	R	
TTG-L2.E3-32	A					R					K	R	
TTG-L2.E3-33	A					R					K	R	
TTT-L2.E3-34	A					R					K		
TTT-L2.E3-35	A					R					K		
TTT-L2.E3-36	A		A			R					K		
TTT-L2.E3-37	A		A			R					K		

b

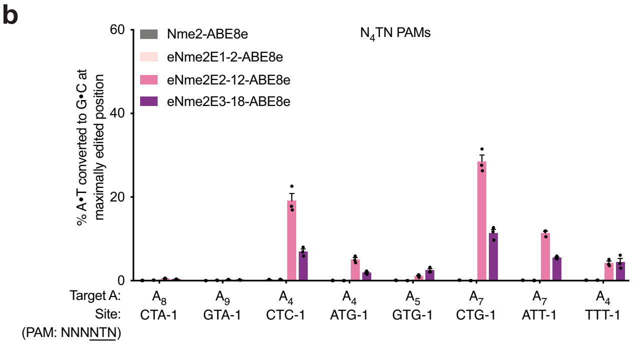

Supplementary Figure 13. Mutation table and representative activity of ePACE3 evolved Nme2Cas9 variants. (a) Genotypes of individually sequenced plaques following ePACE3, with positions varying from wild-type displayed. Clones evolved on different PAMs are delineated by a bold line. Mutations that had previously appeared in ePACE1 and ePACE2 are shown in light pink and magenta, respectively, while novel mutations are shown in purple. **(b)** Adenine base editing activity of a representative ePACE3 clone (E3-18-ABE8e) at eight N_4TN PAM-containing sites in HEK293T cells. Mean±SEM are shown and are representative of *n*=3 independent biological replicates.

a

Passage /PAM	Replicate 1 dilution table						Replicate 2 dilution table						Notes
	ACA	ACG	ACT	TCA	TCG	TCT	ACA	ACG	ACT	TCA	TCG	TCT	
1	20	20		20	20		20	20		20	20		
2	20	20		20	20		20	20		20	20		
3	13	13		13	13		13	13		13	13		Pa2 (1:20) + MP6-diversified Pa1 (1:40)
4	20	20		20	20		20	20		20	20		
5	20	20		20	20		20	20		20	20		
6	20	20		20	20		20	20		20	20		Host strain: 2208+MP6
7	20	20		20	20		20	20		20	20		
8	20	20		20	20		20	20		20	20		Host strain: 2208+MP6
9	20	20		20	20		20	20		20	20		
10	20	20		20	20		20	20		20	20		Host strain: 2208+MP6
11	20	20	**20**	20	20	**20**	20	20	**20**	20	20	**20**	ACT/TCT PAMs added, 1:20 pooled PAMs
12	20	20	20	20	20	20	20	20	20	20	20	20	
13	20	20	20	20	20	20	20	20	20	20	20	20	
14	20	20	20	20	20	20	20	20	20	20	20	20	
15	20	20	20	100	100	100	20	20	20	100	100	100	
16	20	20	20	100	100	100	20	20	20	100	100	100	
17	20	20	20	600	600	600	20	20	20	600	600	600	
18	20	20	20	600	600	600	20	20	20	600	600	600	
19	20	20	20	6000	6000	6000	20	20	20	6000	6000	6000	ACN PAMs - Host strain 2208+MP6
20	20	20	20	6000	6000	6000	20	20	20	6000	6000	6000	ACN PAMs - spiked 1:600 TCN phage

Passage /PAM	Replicate 3 dilution table						Replicate 4 dilution table						Notes
	ACA	ACG	ACT	TCA	TCG	TCT	ACA	ACG	ACT	TCA	TCG	TCT	
1	20	20		20	20		20	20		20	20		
2	20	20		20	20		20	20		20	20		
3	13	13		13	13		13	13		13	13		Pa2 (1:20) + MP6-diversified Pa1 (1:40)
4	20	20		20	20		20	20		20	20		
5	20	20		20	20		20	20		20	20		
6	20	20		20	20		20	20		20	20		Host strain: 2208+MP6
7	20	20		20	20		20	20		20	20		
8	20	20		20	20		20	20		20	20		Host strain: 2208+MP6
9	20	20		20	20		20	20		20	20		
10	20	20		20	20		20	20		20	20		Host strain: 2208+MP6
11	20	20	**20**	20	20	**20**	20	20	**20**	20	20	**20**	ACT/TCT PAMs added, 1:20 pooled PAMs
12	20	20	20	20	20	20	20	20	20	20	20	20	Pa11 (1:40) + Pa6 (1:40)
13	20	20	20	20	20	20	20	20	20	20	20	20	
14	20	20	20	20	20	20	20	20	20	20	20	20	
15	20	20	20	20	20	20	20	20	20	20	20	20	Host strain: 2208+MP6, Pa13 & Pa14
16	20	20	20	20	20	20	20	20	20	20	20	20	
17	20	20	20	20	20	20	20	20	20	20	20	20	
18	20	20	20	20	20	20	20	20	20	20	20	20	
19	20	20	20	20	20	20	20	20	20	20	20	20	ACN PAMs - Host strain 2208+MP6
20	20	20	20	20	20	20	20	20	20	20	20	20	ACN PAMs - spiked 1:600 TCN phage

4	5	6	7	8	9	10	11

LOD

Log_{10} titer (pfu/mL) by qPCR

b

PANCE conditions	
AP	proC-split-Npu-gIII<br>(Target PAMs CTTNCNT, AGGNCNG)
CP	psp-sd8-TadA8e R26G
MP	MP6
SP	Replicate 1 & 2: wild-type split-dNme2Cas9<br>Replicate 3 & 4: ePACE1 & ePACE2 pool,<br>recloned as split Cas9

Supplementary Figure 14. PANCE dilution schedule and titers for N1. SP containing wild-type split-dNme2Cas9 or pooled ePACE1/ePACE2 split-Nme2Cas9 were first diversified in *E.coli* host cells containing pJC175e[2] and MP6[2], isolated, then seeded into PANCE1 (N1, 6

chemostats of each of six N_3WTD PAMs, where W=A or T and D=A,G, or T; 4 replicates). **(a)** Passage stringency schedule and resulting titers (measured by qPCR) for replicates 1 and 2 (top) or replicates 3 and 4 (bottom). Passages were done after 16-24 hr for all passages. For some passages, some conditions were passaged uniquely to others or in a different host cell line, and these changes are listed in the Notes column. Grey coloring represents titers that were not measured or the PAM had not yet been included. All N_3ACD PAMs were unable to support phage propagation, which retroactively was discovered to be attributable to an AP design error (see **Supplementary Note 6**). LOD=limit of detection of qPCR titering, as set by the titer corresponding to the C_q for which the qPCR primers alone had been observed to amplify. **(b)** PANCE conditions used for N1.

NNN<u>ACD</u> PAMs

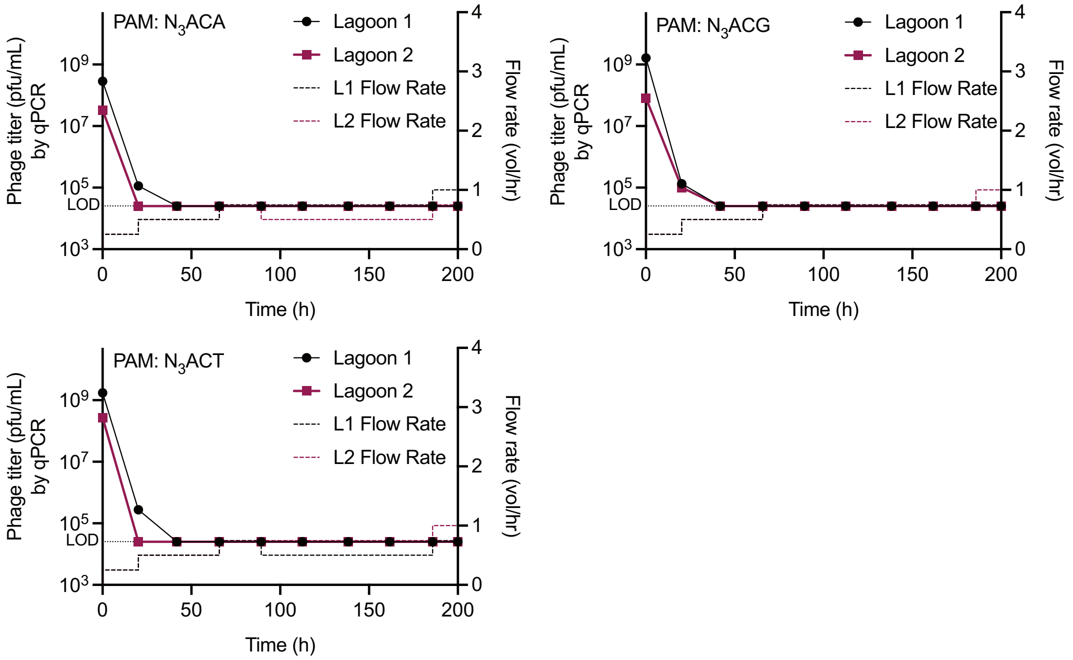

NNN<u>TCD</u> PAMs

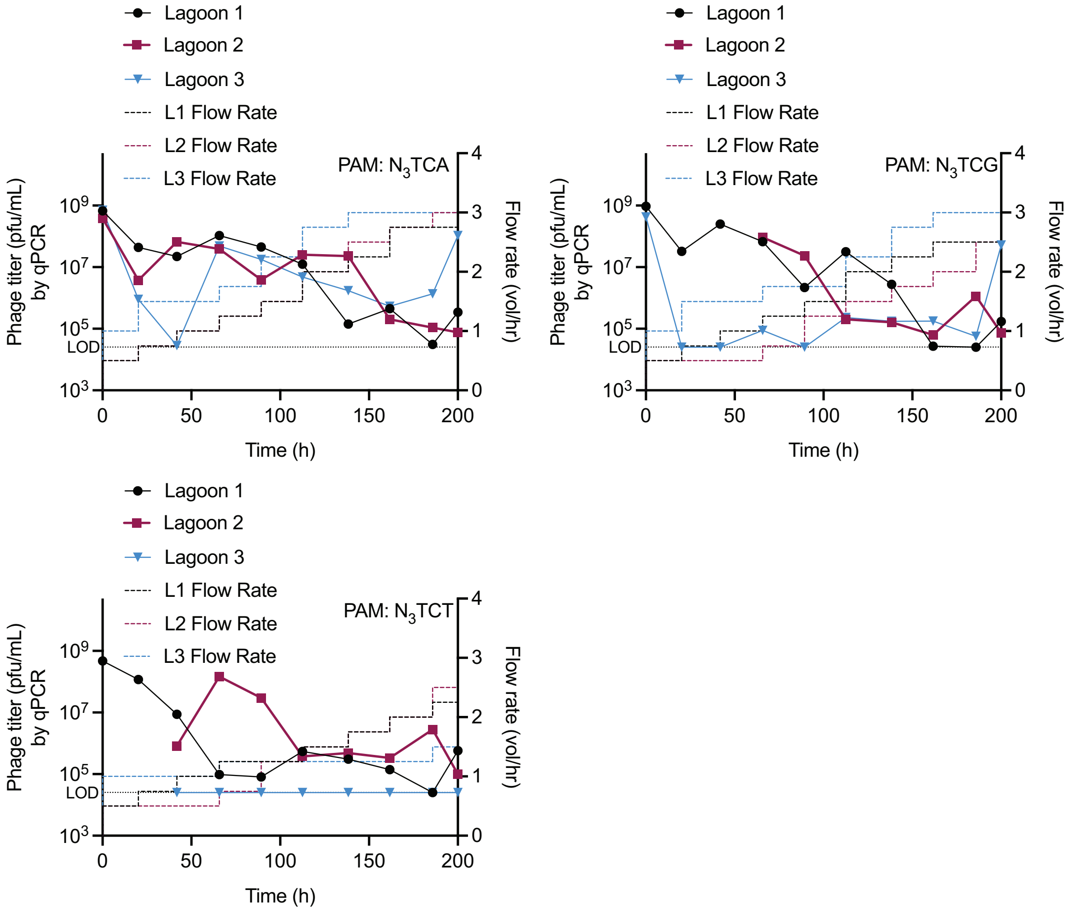

Supplementary Figure 15. Flow rate schedule and titers for ePACE4. SP from N1, passage 20, were combined and seeded into corresponding PAMs in ePACE4 (six chemostats, two lagoons each of the three N_3<u>ACD</u> PAMs, where D=A,G, or T, three lagoons

each of the three N_3<u>TCD</u> PAMs). N1 replicates 1 and 2 were pooled into "Lagoon 1" lagoons, N1 replicates 3 and 4 were pooled into "Lagoon 2" lagoons, and all N1 replicates were pooled into any "Lagoon 3" lagoons. The N_3<u>ACD</u> PAMs all washed out, which retroactively was discovered to be attributable to an AP design error (see **Supplementary Note 6**). Flow rate stringency for each PAM is shown in the plots, as are resulting titers (measured by qPCR). LOD=limit of detection of qPCR titering, as set by the titer corresponding to the C_q for which the qPCR primers alone had been observed to amplify.

a

Passage /PAM	Replicate 1 dilution table							
	CTA	CTC	CTG	CTT	TTA	TTC	TTG	TTT
1	20			20	20		20	20
2	20			20	20		20	20
3	20	**20**	**20**	20	20	**20**	20	20
4	20	20	20	20	20	20	20	20
5	20	20	20	20	20	20	20	20
6	20	20	20	20	20	20	20	20
7								

Passage /PAM	Replicate 2 dilution table							
	CTA	CTC	CTG	CTT	TTA	TTC	TTG	TTT
1	20			20	20		20	20
2	20			20	20		20	20
3	20	**20**	**20**	20	20	**20**	20	20
4	20	20	20	20	20	20	20	20
5	20	20	20	20	20	20	20	20
6	20	20	20	20	20	20	20	20
7								

Passage /PAM	Replicate 3 dilution table							
	CTA	CTC	CTG	CTT	TTA	TTC	TTG	TTT
1	20			20	20		20	20
2	20			20	20		20	20
3	20	**20**	**20**	20	20	**20**	20	20
4	100	100	100	100	100	100	100	100
5	100	100	100	100	100	100	100	100
6	500	500	500	500	500	500	500	500
7	500	500	500	500	500	500	500	500

4	5	6	7	8	9	10	11

LOD Log_{10} titer (pfu/mL) by qPCR

Passage /PAM	Notes
1	
2	
3	missing PAMs added, 1:20 starting phage
4	MP6-diversified Pa3 (1:20)
5	
6	
7	Replicates 1 & 2 stopped

b

**PANCE conditions**	
AP	proC-split-Npu-gIII<br>(Target PAMs CTT<u>NTN</u>T, AGG<u>NTN</u>G)
CP	psp-sd8-TadA8e R26G
MP	MP6
SP	Replicate 1: wild-type split-dNme2Cas9<br>Replicate 2: ePACE1 & ePACE2 pool, recloned as split Cas9<br>Replicate 3: ePACE3 pool, recloned as split Cas9

Supplementary Figure 16. PANCE dilution schedule and titers for N2. SP containing wild-type split-dNme2Cas9, pooled ePACE1/ePACE2 split-Nme2Cas9, or pooled ePACE3 split-Nme2Cas9 were first diversified in *E.coli* host cells containing pJC175e[2] and MP6[2], isolated, then seeded into PANCE2 (N2, eight chemostats of each of eight N_3<u>YTN</u> PAMs, where Y=C or T; three replicates). **(a)** Passage stringency schedule and resulting titers (measured by qPCR) for replicates 1-3. Passages were done after 16-24 hr for all passages. For some passages, some conditions were passaged uniquely to others or in a different host cell line, and these changes are listed in the Notes column. Grey coloring represents titers that were not measured or the PAM had not yet been included. LOD=limit of detection of qPCR titering, as set by the titer corresponding to the C_q for which the qPCR primers alone had been observed to amplify. **(b)** PANCE conditions used for N2.

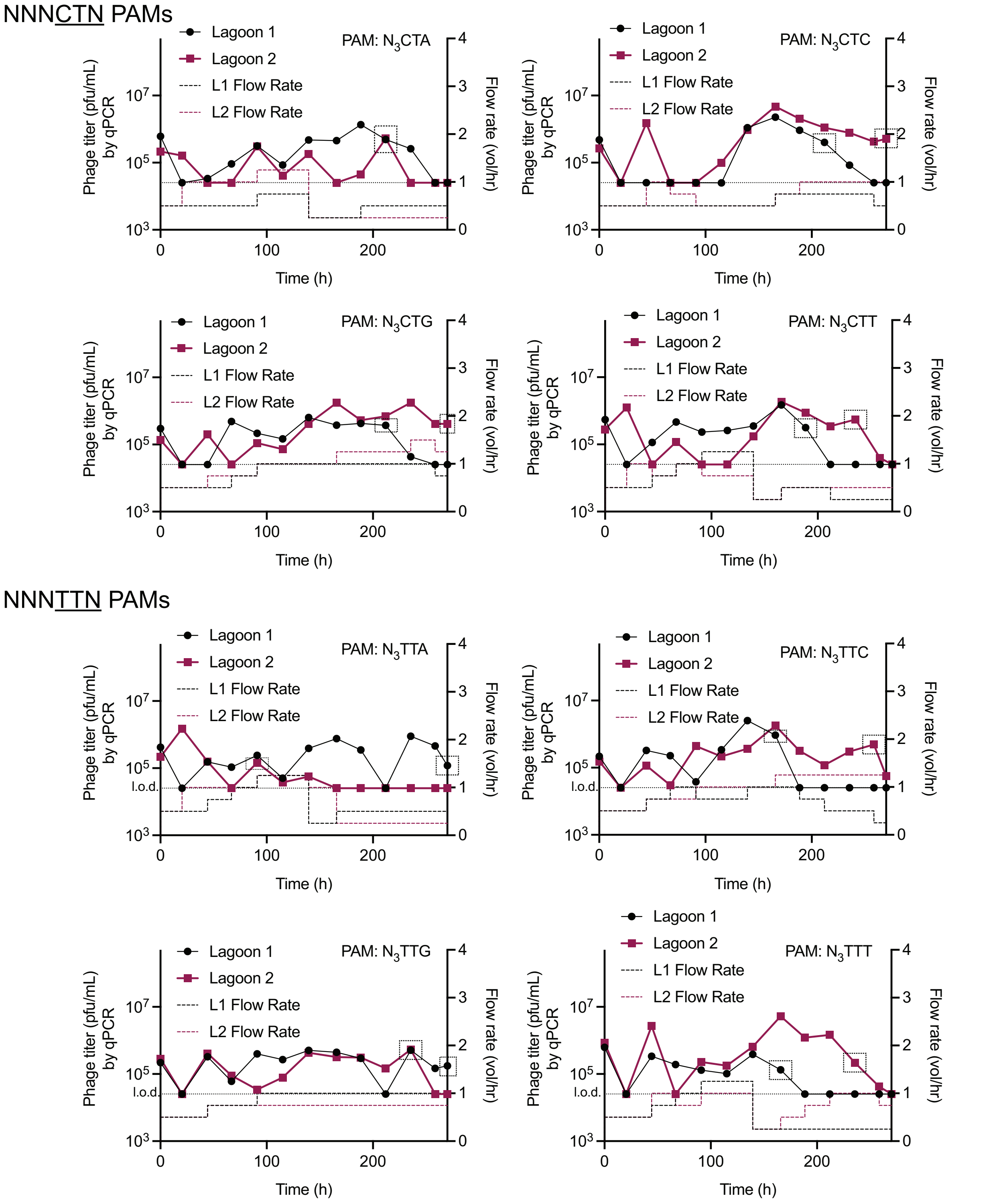

Supplementary Figure 17. Flow rate schedule and titers for ePACE5.
SP from N2 replicate 3, passage 7, were combined and seeded into corresponding PAMs in ePACE5 (eight chemostats, two lagoons each of the eight N_3YTN PAMs, where Y=C or T). Flow rate stringency for each PAM is shown in the plots, as are resulting titers (measured by qPCR). As most lagoons were unable to support consistent phage propagation, the timepoints used for isolating and sequencing phage (**Extended Data Figure 5**) are marked by a black box. LOD=limit of detection of qPCR titering, as set by the titer corresponding to the C_q for which the qPCR primers alone had been observed to amplify.

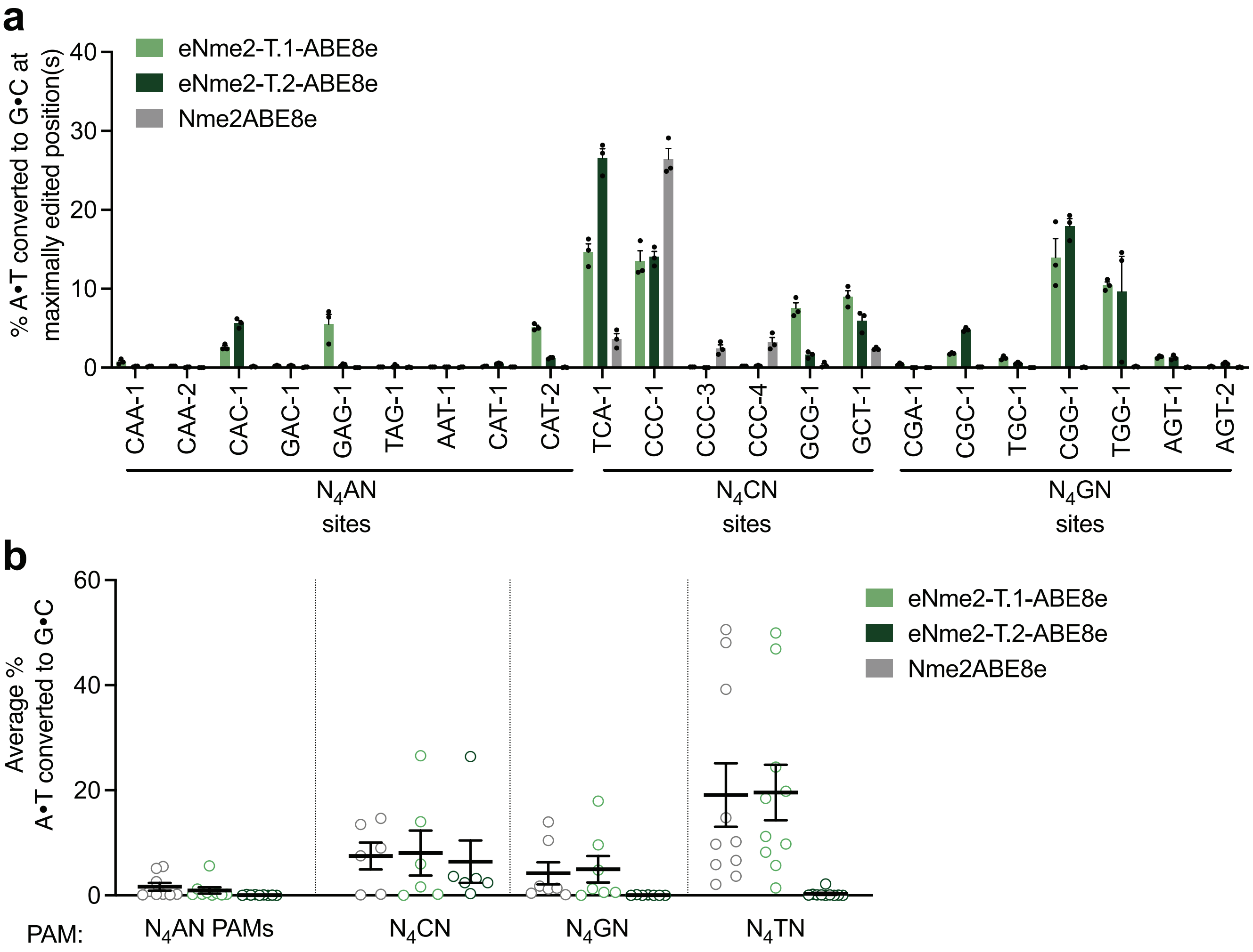

Supplementary Figure 18. eNme2-T.1-ABE8e and eNme2-T.2-ABE8e activity at N_4VN PAM sites. (a) Adenine base editing activity of eNme2-T.1-ABE8e and eNme2-T.2-ABE8e at 22 N_3<u>NVN</u> PAM sites in HEK293T cells. Mean±SEM is shown and reflects the average activity and standard error of *n*=3 replicates at the maximally edited position within each genomic site. **(b)** Adenine base editing activity in (a) pooled by PAM position 5 (N_4<u>N</u>N) identity, also including pooled N_4TN sites from **Extended Data Figure 6a**. Each point represents the average editing of *n*=3 independent biological replicates measured at the maximally edited position within each given genomic site. Mean±SEM is shown and reflects the average activity and standard error of the pooled genomic site averages.

a

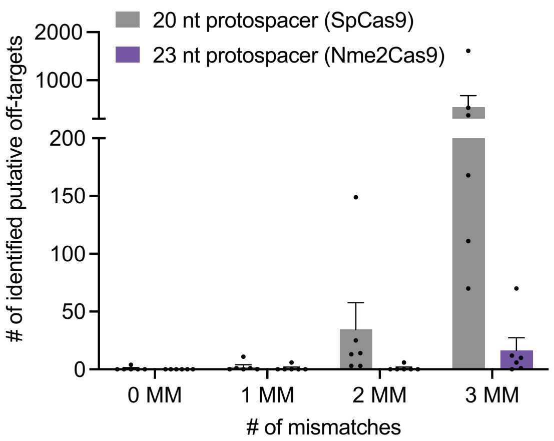

b

	20 nt protospacer (SpCas9)				23 nt protospacer (Nme2Cas9)			
**Site**	**0 MM***	**1 MM**	**2 MM**	**3 MM**	**0 MM***	**1 MM**	**2 MM**	**3 MM**
ACC-1	0	0	3	111	0	0	0	0
GCT-1	0	0	14	271	0	0	0	6
CCC-1	4	11	149	1612	0	6	6	70
CCG-1	0	1	13	168	0	0	0	12
GCA-1	0	1	25	426	0	0	0	10
GCG-1	0	0	3	70	0	0	0	1
**Average**	0.7	2.2	34.5	443.0	0.0	1.0	1.0	16.5

*0 MM tally excludes the on-target site

Supplementary Figure 19. ***In silico*** **prediction of off-target sites with ≤ 3 mismatches for a 20-nt or 23-nt protospacer. (a)** Count of genome-wide (GRCh38) sites with 0, 1, 2, or 3 mismatches to a 20-nt (SpCas9) or 23-nt (Nme2Cas9) protospacer identified with CHOPCHOPv3[3]. Mean±SEM representing identified off-targets at six randomly selected 20-nt or 23-nt protospacers are shown. **(b)** Table listing the number of identified sites with the corresponding number of mismatches to a 20-nt or 23-nt protospacer at six randomly selected genomic sites (see **Supplementary Table 5**).

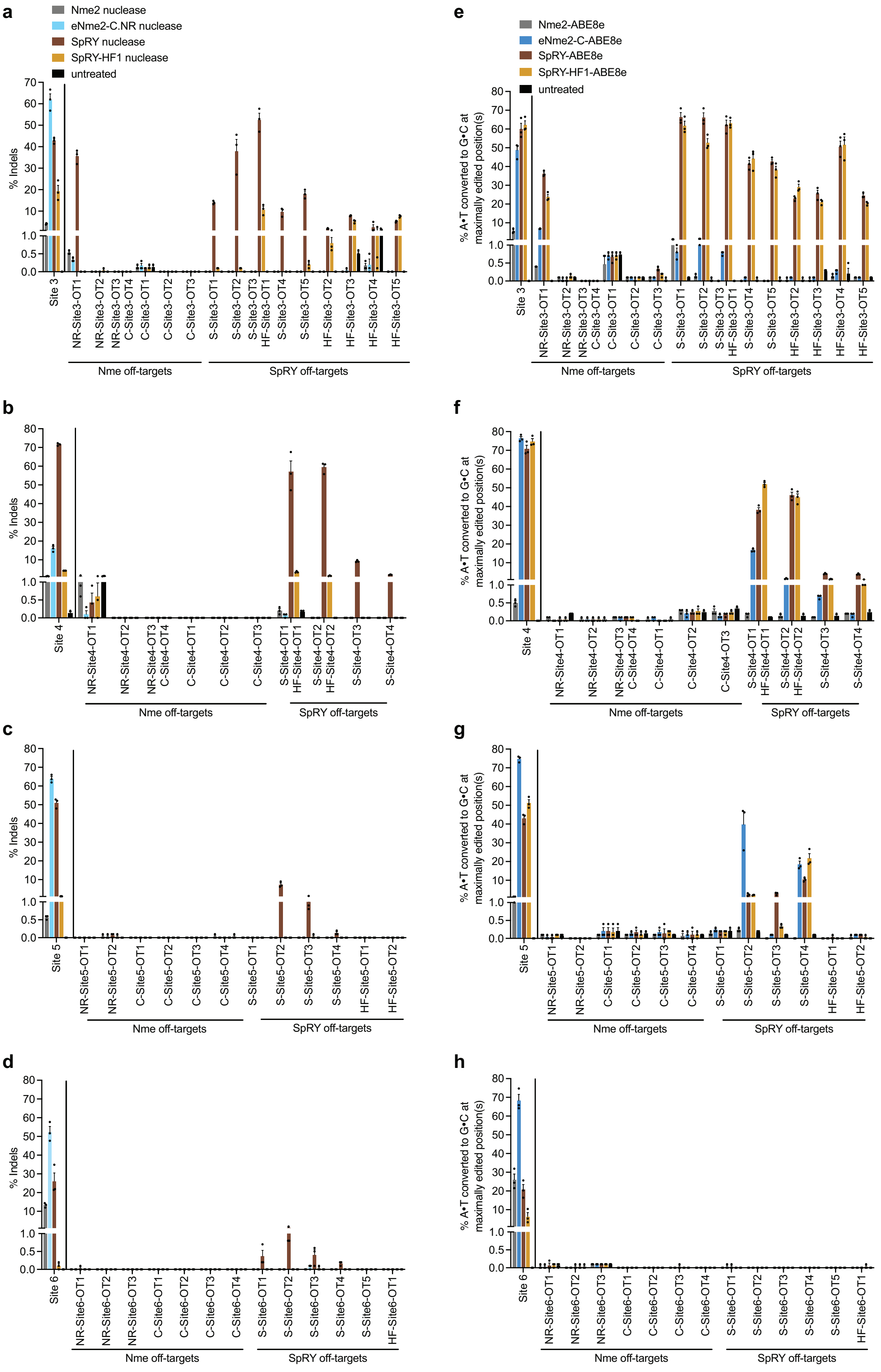

Supplementary Figure 20. High-throughput sequencing validation of GUIDE-seq-identified off-target activity. High-throughput sequencing in HEK293T cells at the top off-

target sites nominated by GUIDE-seq for eNme2-C, eNme2-C.NR, SpRY, or SpRY-HF1 nucleases (**Supplementary Table 3**). Off-target indel formation by Nme2Cas9, eNme2-C.NR, SpRY, or SpRY-HF1 nuclease at nominated off target sites for the sgRNAs targeting Site 3 **(a)**, Site 4 (**b**), Site 5 (**c**), or Site 6 (**d**). Off-target adenine base editing by Nme2-ABE8e, eNme2-C-ABE8e, SpRY-ABE8e, or SpRY-HF1-ABE8e at nominated off-target sites for the sgRNAs targeting Site 3 **(d)**, Site 4 (**e**), Site 5 (**f**), or Site 6 (**g**). Mean±SEM is shown and reflects the average activity and standard error of *n*=3 independent biological replicates measured at the maximally edited position within each given genomic site. On-target activity is shown at the left-most entry for each site.

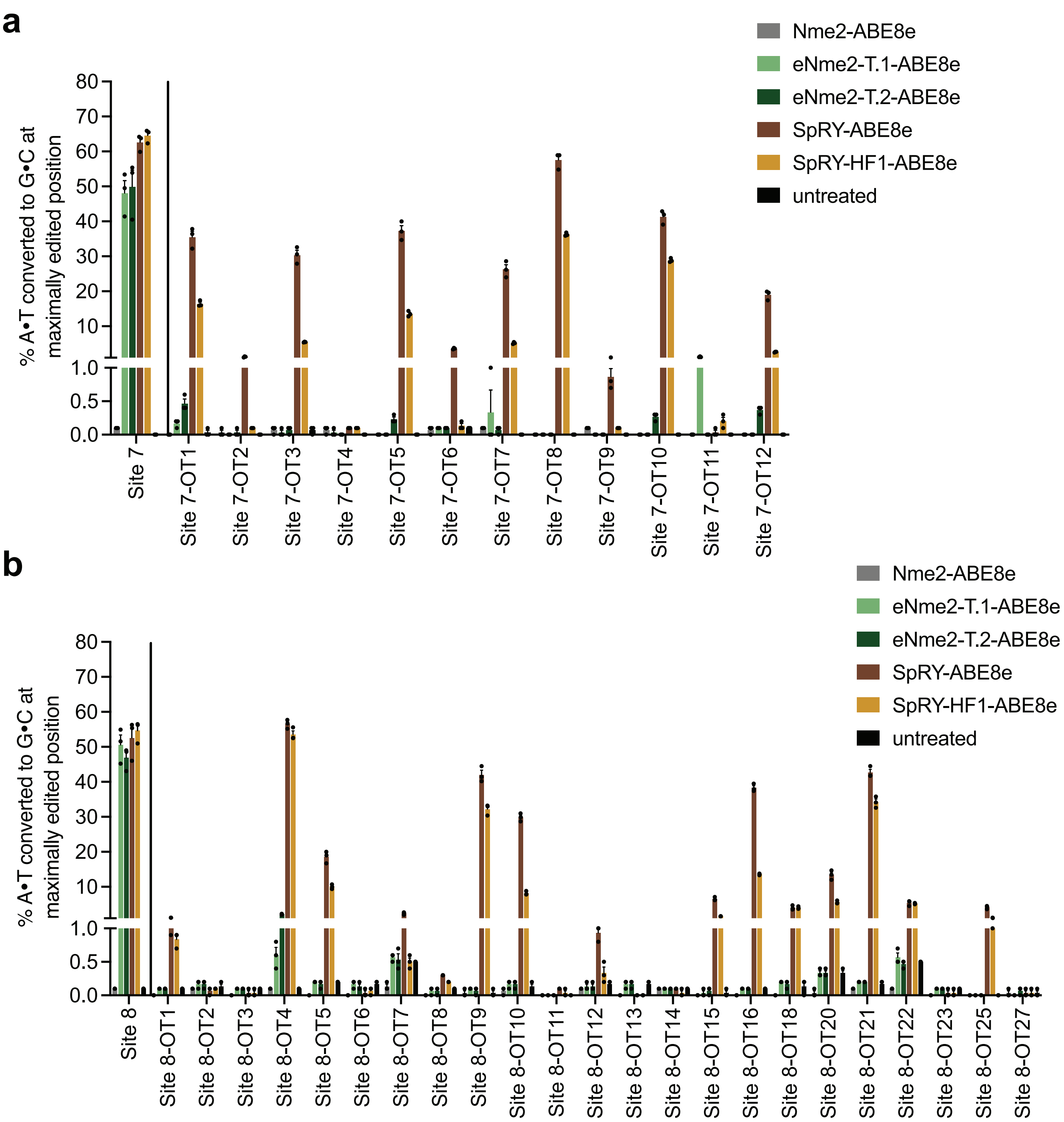

Supplementary Figure 21. Off-target adenine base editing at *in silico*-predicted off-target sites for SpRY-ABE8e, SpRY-HF1-ABE8e, eNme2-T.1-ABE8e and eNme2-T.2-ABE8e. (a) Off-target adenine base editing by SpRY-ABE8e, SpRY-HF1-ABE8e, eNme2-T.1-ABE8e and eNme2-T.2-ABE8e at (**a**) 12 computationally determined off-targets of a protospacer-matched sgRNA (Site 7) or (**b**) 23 computationally determined off-targets of a protospacer matched sgRNA (Site 8). Mean±SEM is shown and reflects the average activity and standard error of the of n=3 independent biological replicates measured at the maximally edited position within each given genomic site. On-target activity is shown at the left-most entry for each site.

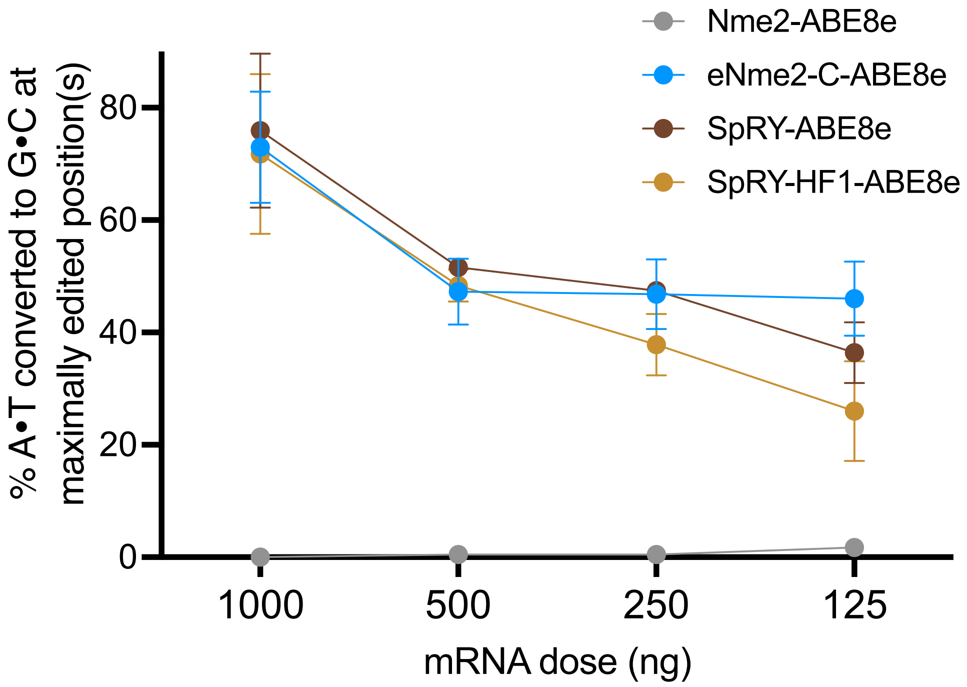

Supplementary Figure 22. **Dose-dependent adenine base editing activity in primary human dermal fibroblasts.** Dose titration of mRNA encoding Nme2-ABE8e, eNme2-C-ABE8e, SpRY-ABE8e, or SpRY-HF1-ABE8e electroporated into primary human dermal fibroblasts together with synthetic guide RNA targeting either the GCT-2 or CCG-1 site (**Supplementary Table 5**). Mean±SEM is shown and reflects the average activity of one biological replicate measured for each dose targeting the two different endogenous genomic sites.

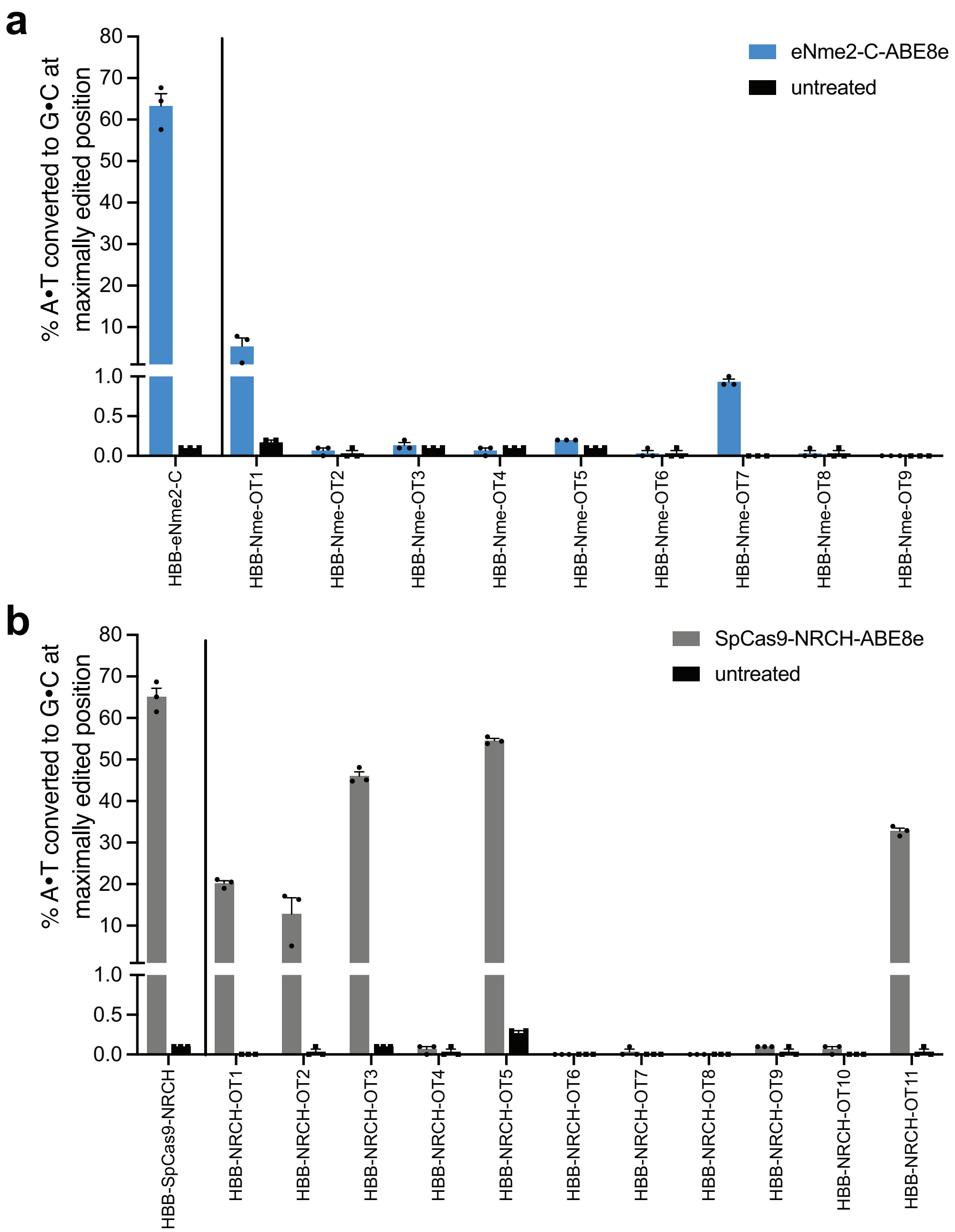

Supplementary Figure 23. Off-target adenine base editing at in silico-predicted off-target sites for SpCas9-NRCH and eNme2-C sgRNAs targeting the *HBB* sickle-cell disease mutation. **(a)** Off-target adenine base editing by eNme2-C-ABE8e at nine computationally nominated off-target sites for the sgRNA targeting the *HBB* sickle-cell disease mutation. **(b)** Off-target adenine base editing by SpCas9-NRCH-ABE8e at 11 computationally nominated off-target sites for the sgRNA targeting the *HBB* sickle-cell disease mutation. Mean±SEM is shown and reflects the average activity and standard error of the of *n*=3 independent biological replicates measured at the maximally edited position within each given genomic site. On-target activity is shown at the left-most entry for each site.

Supplementary Table 1. BE-PPA library editing data
(See the separately provided Excel file.)

Supplementary Table 2. CHOPCHOPv3 identified off-target sites of protospacer-matched sgRNAs comparing eNme2-C to SpRY and SpRY-HF1

**Target site or off-target site**	**Gene**	**Protospacer sequence** (5'-3'; top: Nme2Cas9, bottom: SpRY)	**PAM** (top: Nme2Cas9; bottom: SpRY)	**Mismatches** (PAM proximal^) (top: Nme2Cas9; bottom: SpRY)
Site 1	*PDCD1*	GCAGATCCCACAGGCGCCCTGGC<br>GATCCCACAGGCGCCCTGGC	CAGTCG<br>CAG	–
Site1-OT1	Intergenic	GCAttTCCCACAGGCGCCCTGGC<br>ttTCCCACAGGCGCCCTGGC	GATGCC<br>GAT	2 (0)<br>2 (0)
Site 1-OT2	*PLCH2*	tgAGgTCCCACAGGCcCCCTGGC<br>GgTCCCACAGGCcCCCTGGC	GCAGCC<br>GCA	4 (1)<br>2 (1)
Site 1-OT3	Intergenic	tggGcTCCCACAGGCcCCCTGGC<br>GcTCCCACAGGCcCCCTGGC	CTCCCC<br>CTC	5 (1)<br>2 (1)
Site 1-OT4	*TCF20*	agAGATaCCACAGGCaCCCTGGC<br>GATaCCACAGGCaCCCTGGC	ATGATA<br>ATG	4 (1)<br>2 (1)
Site 1-OT5	*CXXC5*	GCtGcTCCCAgAGGCGCCCTGGC<br>GcTCCCAgAGGCGCCCTGGC	TCTGCA<br>TCT	3 (0)<br>2 (0)
*Site 1-OT6**	Intergenic	agAGATCaCcCAGGCGCCCTGGC<br>GATCaCcCAGGCGCCCTGGC	TCATGC<br>TCA	4 (0)<br>2 (0)
*Site 1-OT7**	*SAPCD2P4*	tggGAcCCCACAtGCGCCCTGGC<br>GAcCCCACAtGCGCCCTGGC	CGGGAC<br>CGG	5 (0)<br>2 (0)
Site 2	*FANCF*	GCTGCAGAAGGGATTCCATGAGG<br>GCAGAAGGGATTCCATGAGG	TGCGCG<br>TGC	–
Site 2-OT1	*C11orf80*	tgTGaAGAAGGGtTTCCATGAGG<br>GaAGAAGGGtTTCCATGAGG	AGATAC<br>AGA	4 (0)<br>2 (0)
Site 2-OT2	*ANO3*	GaTtCtGAAGGGATTCCATGAGG<br>tCtGAAGGGATTCCATGAGG	TCTAAA<br>TCT	3 (0)<br>2 (0)
Site 2-OT3	*MEAK7*	caTGgAGAAGGGATcCCATGAGG<br>GgAGAAGGGATcCCATGAGG	ACAAGG<br>ACA	4 (1)<br>2 (1)
Site 2-OT4	Intergenic	GgTaCAGAAGGGcTTCCATGAGG<br>aCAGAAGGGcTTCCATGAGG	CTGGGT<br>CTG	3 (0)<br>2 (0)
Site 2-OT5	*SLC19A1*	GgaGCAGAAGGcATTtCATGAGG<br>GCAGAAGGcATTtCATGAGG	GGTCAA<br>GGT	4 (1)<br>2 (1)
Site 2-OT6	*LOC105374492*	cagGCAGAAaGGAaTCCATGAGG<br>GCAGAAaGGAaTCCATGAGG	ACTCCC<br>ACT	5 (1)<br>2 (1)
Site 2-OT7	*MAP9*	aagGCAGAtGGGATTCCtTGAGG<br>GCAGAtGGGATTCCtTGAGG	TCTGGA<br>TCT	5 (1)<br>2 (1)
Site 2-OT8	Intergenic	aagGCAGAAGGaATTCCATGAGt<br>GCAGAAGGaATTCCATGAGt	TAAAGT<br>TAA	5 (1)<br>2 (1)
Site 2-OT9	*RIPOR2*	ctTGCtGAAGGGATTCaATGAGG<br>GCtGAAGGGATTCaATGAGG	TGCTAC<br>TGC	4 (1)<br>2 (1)
Site 2-OT10	Intergenic	GagGCAGgAGGGATTCCcTGAGG<br>GCAGgAGGGATTCCcTGAGG	AAGTGA<br>AAG	4 (1)<br>2 (1)
Site 2-OT11	Intergenic	agaGCgGAAGGGATgCCATGAGG<br>GCgGAAGGGATgCCATGAGG	GATCCG<br>GAT	5(1)<br>2(1)
*Site 2-OT12**	Intergenic	aaaGgAGAAGGaATTCCATGAGG<br>GgAGAAGGaATTCCATGAGG	ATACAA<br>ATA	5(0)<br>2(0)

*site did not sequence well (mixed bases) with HTS primers that were tried, see **Supplementary Table 8**. These sites were excluded from further analysis.

^PAM proximal defined as positions within 10 bases of the PAM

Supplementary Table 3. GUIDE-Seq identified off-target sites
(See separately attached Excel file.)

Supplementary Table 4. CHOPCHOPv3 identified off-target sites of protospacer-matched sgRNAs comparing eNme2-T.1 and eNme2-T.2 to SpRY and SpRY-HF1

**Target site or off-target site**	**Gene**	**Protospacer sequence** (5'-3'; top: Nme2Cas9, bottom: SpRY)	**PAM** (top: Nme2Cas9; bottom: SpRY)	**Mismatches** (PAM proximal^) (top: Nme2Cas9; bottom: SpRY)
Site 7	*GRIN2B*	GTGGGCTGTAACAGGAGGGCCAG<br>GGCTGTAACAGGAGGGCCAG	GAGATT<br>GAG	–
Site 7-OT1	*SHANK2*	aaGGGCTGcAgCAGGAGGGCCAG<br>GGCTGcAgCAGGAGGGCCAG	GTGCTC<br>GTG	4 (0)<br>2 (0)
Site 7-OT2	*PRPF40B*	aaGGGCTGcAACAGGAGGGaCAG<br>GGCTGcAACAGGAGGGaCAG	GGTACC<br>GGT	4 (1)<br>2 (1)
Site 7-OT3	*ATP11A*	GgtGGCTGTgACAGcAGGGCCAG<br>GGCTGTgACAGcAGGGCCAG	GAGCAG<br>GAG	4 (1)<br>2 (1)
Site 7-OT4	*TMEM63C*	aaGGGCTGgAACtGGAGGGCCAG<br>GGCTGgAACtGGAGGGCCAG	AACCAG<br>AAC	4 (0)<br>2 (1)
Site 7-OT5	*AQP8*	catGGCTcTAtCAGGAGGGCCAG<br>GGCTcTAtCAGGAGGGCCAG	GTGACG<br>GTG	5 (0)<br>2 (0)
Site 7-OT6	Intergenic	GgaGGCTGTcACAGcAGGGCCAG<br>GGCTGTcACAGcAGGGCCAG	CAGCTC<br>CAG	4 (1)<br>2 (1)
Site 7-OT7	*UNC13D*	agtGGCTGgAACAGGAaGGCCAG<br>GGCTGgAACAGGAaGGCCAG	GAGGCA<br>GAG	5 (1)<br>2 (1)
Site 7-OT8	*ZNF566*	GgattCTGTAACAGGAGGGCCAG<br>ttCTGTAACAGGAGGGCCAG	AGCACT<br>AGC	4 (0)<br>2 (0)
Site 7-OT9	*SIX3*	cTcGGCTGaAACAGGAGGGCgAG<br>GGCTGaAACAGGAGGGCgAG	TACATG<br>TAC	4 (1)<br>2 (1)
Site 7-OT10	Intergenic	taGGaCTGaAACAGGAGGGCCAG<br>GaCTGaAACAGGAGGGCCAG	GCAGAG<br>GCA	4 (0)<br>2 (0)
Site 7-OT11	Intergenic	tgaGcCTGTgACAGGAGGGCCAG<br>GcCTGTgACAGGAGGGCCAG	CTGCCA<br>CTG	5 (0)<br>2 (0)
Site 7-OT12	Intergenic	cTGaGCTGTcACAGGAGGGCCAG<br>aGCTGTcACAGGAGGGCCAG	TGTCAA<br>TGT	3 (0)<br>2 (0)
*Site 7-OT13**	*SAPCD2P4*	cTtGGCTGgAACAGGAGGaCCAG<br>GGCTGgAACAGGAGGaCCAG	CCTTCC<br>CCT	4 (1)<br>2 (1)
Site 8	*SEC61B*	ATATTGGAGATGAGGGTGGCAAG<br>TTGGAGATGAGGGTGGCAAG	GCGCTG<br>GCG	–
Site 8-OT1	*PIGR*	cTATTGGgGATGAGGGTGGCAtG<br>TTGGgGATGAGGGTGGCAtG	AGGAGG<br>AGG	3 (1)<br>2 (1)
Site 8-OT2	Intergenic	tacTTGGAGATGgGGGTGGgAAG<br>TTGGAGATGgGGGTGGgAAG	GGCTTG<br>GGC	5 (1)<br>2 (1)
Site 8-OT3	*TCF7L2*	cgcTTGGcGATGAGGGTGGCAtG<br>TTGGcGATGAGGGTGGCAtG	GCCAAG<br>GCC	5 (1)<br>2 (1)
Site 8-OT4	*LOC100287189*	aTgcTGGAGATGAGGGTGGCAAG<br>cTGGAGATGAGGGTGGCAAG	GAGCTG<br>GAG	5 (1)<br>2 (1)
Site 8-OT5	Intergenic	tTccTGGAGATGAGGGTGGgAAG<br>cTGGAGATGAGGGTGGgAAG	GAAGTT<br>GAA	4 (1)<br>2 (1)
Site 8-OT6	*ADAMTS15*	gctTTGGtGATGAGGGTGGgAAG<br>TTGGtGATGAGGGTGGgAAG	GGCTAA<br>GGC	5 (1)<br>2 (1)
Site 8-OT7	Intergenic	aTgTgGGAGATGAGGGTGGCAgG<br>TgGGAGATGAGGGTGGCAgG	AGCGGA<br>AGC	4 (1)<br>2 (1)
Site 8-OT8	Intergenic	gccTTGGgGATGAGGGTGGgAAG<br>TTGGgGATGAGGGTGGgAAG	CAGAAG<br>CAG	5 (1)<br>2 (1)
Site 8-OT9	*FREM2*	tgATaGtAGATGAGGGTGGCAAG<br>TaGtAGATGAGGGTGGCAAG	GAAATA<br>GAA	4 (0)<br>2 (0)

Site 8-OT10	Intergenic	ggAcTGGAGATGgGGGTGGCAAG<br>cTGGAGATGgGGGTGGCAAG	CAGGAA<br>CAG	4 (0)<br>2 (0)
Site 8-OT11	Intergenic	gatTTaGAGATGAGGGTGcCAAG<br>TTaGAGATGAGGGTGcCAAG	GCTCAG<br>GCT	5 (1)<br>2 (1)
Site 8-OT12	*CMIP*	cTgaTGGAGATGAGGGTGGCAgG<br>aTGGAGATGAGGGTGGCAgG	TCGGAG<br>TCG	4 (1)<br>2 (1)
Site 8-OT13	Intergenic	gagTTGGAGcTGAGGGTGtCAAG<br>TTGGAGcTGAGGGTGtCAAG	GAAAGC<br>GAA	5 (1)<br>2 (1)
Site 8-OT14	*LGI4*	gcATTGGAGATGcGGGTGGCAAt<br>TTGGAGATGcGGGTGGCAAt	CTTTTT<br>CTT	4 (1)<br>2 (1)
Site 8-OT15	Intergenic	tTATTGGAGATaAGGGTGGgAAG<br>TTGGAGATaAGGGTGGgAAG	AGCTCT<br>AGC	3 (1)<br>2 (1)
Site 8-OT16	Intergenic	ggcTgGGAGAaGAGGGTGGCAAG<br>TgGGAGAaGAGGGTGGCAAG	GGAGGT<br>GGA	5 (0)<br>2 (0)
*Site 8-OT17**	Centromere	tTtTTGaAGATGAGGGTGGCAtG<br>TTGaAGATGAGGGTGGCAtG	GTCCCA<br>GTC	4 (1)<br>2 (1)
Site 8-OT18	Intergenic	tggTTaGAGATGAGGGTGtCAAG<br>TTaGAGATGAGGGTGtCAAG	AGGCAC<br>AGG	5 (1)<br>2 (1)
*Site 8-OT19**	*GRIA1*	taAaTGGAGATGAGGGTGGaAAG<br>aTGGAGATGAGGGTGGaAAG	ATGCAT<br>ATG	4 (1)<br>2 (1)
Site 8-OT20	*NCOA7*	cTccTGGAGATGAGGGTGGCAaG<br>cTGGAGATGAGGGTGGCAaG	GAAGGG<br>GAA	4 (1)<br>2 (1)
Site 8-OT21	*PDE1C*	AagTgGGAGATGAGGGTGGCAAG<br>TgGGAGATGAGGGTGGCAAG	GCTTAC<br>GCT	3 (0)<br>1 (0)
Site 8-OT22	*PDE1C*	AagTgGGAGATGcGGGTGGCAAG<br>TgGGAGATGcGGGTGGCAAG	GTGGCG<br>GTG	4 (0)<br>2 (0)
Site 8-OT23	Intergenic	tccTTtGAGATGAGGGTGcCAAG<br>TTtGAGATGAGGGTGcCAAG	GAAATA<br>GAA	5 (1)<br>2 (1)
*Site 8-OT24**	Intergenic	tacTTGGAGAaGAGGGaGGCAAG<br>TTGGAGAaGAGGGaGGCAAG	AGGTAG<br>AGG	5 (1)<br>2 (1)
Site 8-OT25	*DAP2IP*	ggccTGGAGATGAGGGTGGCAgG<br>cTGGAGATGAGGGTGGCAgG	CAGGCT<br>CAG	5 (1)<br>2 (1)
*Site 8-OT26**	*TTLL11*	AggaTGGAGATGAGGcTGGCAAG<br>aTGGAGATGAGGcTGGCAAG	TCTAAT<br>TCT	4 (1)<br>2 (1)
Site 8-OT27	Intergenic	AaATTGGccATGAGGGTGGCAAG<br>TTGGccATGAGGGTGGCAAG	TCGGTC<br>TCG	4 (0)<br>2 (0)

*site did not sequence well (mixed bases) with HTS primers that were tried, see **Supplementary Table 8**. These sites were excluded from further analysis.
^PAM proximal defined as positions within 10 bases of the PAM

Supplementary Table 5. List of target sites
(See separately attached Excel file.)

Supplementary Table 6. CHOPCHOPv3-identified off-target sites of sgRNAs targeting the *HBB* sickle-cell disease mutation

Target site or off-target site	Gene	Protospacer sequence (5' to 3')	PAM	Mismatches (PAM proximal^)	Previously validated?
HBB-eNme2-C	*HBB*	AGACTTCTCCACAGGAGTCAGGT	GCACCA	–	
HBB-Nme-OT1	*HBB*	AGACTTCTCCtCAGGAGTCAGaT	GCACCA	2(1)	Overlaps HBB-NRCH-OT2
HBB-Nme-OT2	Intergenic	ttACTTCTCCACAGcAGTCAGGT	TTTGAT	3 (1)	
HBB-Nme-OT3	*CNTN5*	AaACTTCTCCACtGGAGTCAGGg	CAGGAG	3 (1)	
HBB-Nme-OT4	Intergenic	gagCTTCTtCACAaGAGTCAGGT	AACCTA	5 (1)	
HBB-Nme-OT5	*CGNL1*	ccctTTCTCCACAGGAGTCAGGa	GAGACA	5 (1)	Overlaps HBB-NRCH-OT3
HBB-Nme-OT6	*NUP50*	tcACTTCTCCAGAGGtGTCAGGT	AACTAA	3 (1)	
HBB-Nme-OT7	Intergenic	ctgCTTCTCCAcAaGAGTCAGGT	ATCACT	5(1)	
HBB-Nme-OT8	Intergenic	AagtTTCTCCAGAGGAGTCAGGT	TAGGAG	3(0)	Overlaps HBB-NRCH-OT10
HBB-Nme-OT9	Intergenic	cttCTTCTCCAtAGGAGTCAGaT	GTGATG	5(1)	Overlaps HBB-NRCH-OT11
HBB-SpCas9-NRCH	*HBB*	TTCTCCACAGGAGTCAGGTG	CAC	–	
HBB-NRCH-OT1	*TET1*	TaaTCCACAGGAGTCAGGTG	CAC	2(0)	Y[4]
HBB-NRCH-OT2	*HBB*	TTCTCCtCAGGAGTCAGaTG	AGA	2(1)	Y[4]
HBB-NRCH-OT3	*CGNL1*	TTCTCCACAGGAGTCAGGaG	ATA	1(1)	
HBB-NRCH-OT4	*PCSK6*	TTCTCCAgAGGAGTCAGGgG	GGT	2(1)	Y[4]
HBB-NRCH-OT5	*CFDP1*	cTCcCCACAGGAGTCAGGTG	CCT	2(0)	Y[4]
HBB-NRCH-OT6	*ETFB*	TTCTCCtCAGGAGTCAGGaG	GGC	2(1)	Y[4]
HBB-NRCH-OT7	Intergenic	TTCTCCcCAGGAGcCAGGTG	GCC	2(1)	Y[4]
HBB-NRCH-OT8	*LOC105377396*	TTCTgCACAGGAGTCAtGTG	AAG	2(1)	Y[4]
HBB-NRCH-OT9	*LOC105374898*	TTCTCCcCgGGAGTCAGGTG	CAC	2(0)	Y[4]
HBB-NRCH-OT10	Intergenic	TTCTCCAgAGGAGTCAGGTt	AGG	2(1)	
HBB-NRCH-OT11	Intergenic	TTCTCCAtAGGAGTCAGaTG	TGA	2(1)	Y[4]

Supplementary Table 7. Plasmids and selection phage (SP) used in this work

Name	Usage (resistance)	Origin	ORF1 (prom [RBS] genes)	ORF2 (prom [RBS] genes)
pTPH353e	AP validation (carb^R)	SC101	P_{psp}[5] [SD8[6]] gIII(1-18)-NpuN-32aa linker-NpuC-gIII(18-425), luxAB	
pTPH353e-d	AP validation (carb^R)	SC101	P_{psp}[5] [SD8[6]] gIII(1-18)-NpuN-32aa linker-NpuC(C1A)-gIII(18-425), luxAB	
pTPH353e-d stops	AP validation (carb^R)	SC101	P_{psp}[5] [SD8[6]] gIII(1-18)-NpuN-32aa linker (double stop codon)-NpuC-gIII(18-425), luxAB	
pTPH401	AP validation (carb^R)	ColE1	P_{ProC}[7] [sd8[6]] gIII(1-18)-NpuN-64aa linker(Evo-1 protospacer, NNNNCC PAM)-NpuC-gIII(18-425), luxAB	P_{lac} Evo-1 sgRNA
pTPH400	AP validation (carb^R)	ColE1	P_{ProC}[7] [sd8[6]] gIII(1-18)-NpuN-121aa linker(Evo-1 protospacer, NNNNCC PAM)-NpuC-gIII(18-425), luxAB	P_{lac} Evo-1 sgRNA
pTPH397	AP validation (carb^R)	ColE1	P_{ProC}[7] [sd8[6]] gIII(1-18)-NpuN-32aa linker(Evo-1 protospacer, NNNNCC PAM)-NpuC-gIII(18-425), luxAB	P_{lac} Evo-1 sgRNA
pTPH397b	AP validation (carb^R)	ColE1	P_{ProC}[7] [sd8[6]] gIII(1-18)-NpuN-32aa linker(Evo-2 protospacer, NNNNCC PAM)-NpuC-gIII(18-425), luxAB	P_{lac} Evo-2 sgRNA
pTPH397c	AP validation (carb^R)	ColE1	P_{ProC}[7] [sd8[6]] gIII(1-18)-NpuN-32aa linker(Evo-3 protospacer, NNNNCC PAM)-NpuC-gIII(18-425), luxAB	P_{lac} Evo-3 sgRNA
pTPH405b	AP: ePACE1-2 (carb^R)	ColE1	P_{ProC}[7] [sd8[6]] gIII(1-18)-NpuN-32aa linker(Evo-2 protospacer, varied PAMs)-NpuC-gIII(18-425), luxAB	P_{lac} Evo-2 sgRNA
pTPH405c	AP: ePACE3 (carb^R)	ColE1	P_{ProC}[7] [sd8[6]] gIII(1-18 recoded)-NpuN-32aa linker(Evo-2 protospacer, varied PAMs)-NpuC-gIII(18-425), luxAB	P_{lac} Evo-2 sgRNA
pTPH412 WT sd8	CP validation (kan^R)	SC101	P_{psp}[5] [sd8[6]] TadABE8e-gp41-8N	
pTPH412 WT sd2	CP validation (kan^R)	SC101	P_{psp}[5] [sd2[6]] TadABE8e-gp41-8N	
pTPH412 R26G sd8	CP: ePACE3-5 (kan^R)	SC101	P_{psp}[5] [sd8[6]] TadABE8e(R26G)-gp41-8N	
pTPH412 R26G sd2	CP validation (kan^R)	SC101	P_{psp}[5] [sd2[6]] TadABE8e(R26G)-gp41-8N	
pTPH418b	AP: ePACE4-5 (carb^R)	ColE1	P_{ProC}[7] [sd8[6]] gIII(1-18 recoded)-NpuN-45aa linker(2 x Evo-4 protospacer, varied PAMs)-NpuC-gIII(18-425), luxAB	P_{lac} Evo-4 sgRNA
pTPH343c	CBE-PPA (spec^R)	ColE1	P_{BAD} [SD8[6]] rAPOBEC1-dSpCas9-UGI	P_{lac} CBE-PPA sgRNA
pTPH342	CBE-PPA library (carb^R)	SC101	CBE-PPA protospacer	P_{Pro1} [SD8] sfGFP (inactive)
pTPH413	ABE-PPA (cm^R)	p15A	P_{BAD} [sd4u[6]] TadABE8e-dNme2Cas9 variant	P_{lac} ABE-PPA sgRNA
pTPH424	ABE-PPA library (carb^R)	SC101	ABE-PPA protospacer	P_{Pro1} [SD8] sfGFP (inactive)
Nme-IVT	IVT template (carb^R)	pUC	$P_{T7(mutated)}$[8] Nme2ABE8e variant	
SpRY-IVT	IVT template (carb^R)	pUC	$P_{T7(mutated)}$[8] SpRYABE8e or SpRY-HF1-ABE8e variant	
Nme sgRNA	Mammalian guide expression (carb^R)	pUC	P_{hU6} Nme2Cas9 sgRNA	
SpRY sgRNA	Mammalian guide expression (carb^R)	pUC	P_{hU6} SpRY/SpRY-HF1 sgRNA	
Nme2ABE8e variants	Mammalian expression of adenine base editor (carb^R)	pUC	P_{CMV} Nme2ABE8e variant	
SpRYABE8e variants	Mammalian expression of adenine base editor (carb^R)	pUC	P_{CMV} SpRY/SpRY-HF1-ABE8e variant	
Nme2BE4 variants	Mammalian expression of cytosine base editor (carb^R)	pUC	P_{CMV} Nme2BE4 variant	
SpRYBE4 variants	Mammalian expression of adenine base editor (carb^R)	pUC	P_{CMV} SpRY/SpRY-HF1-BE4 variant	
Nme2 nuclease variants	Mammalian expression of nuclease (carb^R)	pUC	P_{CMV} Nme2Cas9 nuclease variant	
SpRY nuclease variants	Mammalian expression of nuclease (carb^R)	pUC	P_{CMV} SpRY/SpRY-HF1 nuclease variant	
SP391c	ePACE1-2, ΔgIII SP, backbone recoded[9]	M13 f1	P_{gIII} [SD4[4]] TadABE8e-dNme2Cas9 variant	
SP404	ePACE3-5, ΔgIII SP, backbone recoded[9]	M13 f1	P_{gIII} [SD4[4]] gp41-C-dNme2Cas9 variant	

Supplementary Table 8. Primers used in this work

Amplicon	Fw (5'-3')	Rv (5'-3')	Purpose
SP backbone	CACCGTTCATCTGTCCTCTTT	CGACCTGCTCCATGTTACTTAG	qPCR estimation of SP titer
SP insert	TAATGGAAACTTCCTCATGAAAAAGTCTTTAG	ACAGAGAGAATAACATAAAACAGGGAAGC	PCR amplification of SP insert for Sanger sequencing
BE-PPA library insert	ACACTCTTTCCCTACACGACGCTCTTCCGATCTNNNNCAATACGCAACGCCTCTC	TGGAGTTCAGACGTGTGCTCTTCCGATCCTTGTCTGTAAGCGGATGC	HTS of BE-PPA libraries (both CBE and ABE-PPE)
CBE-PPA 5N oligo library (1,024 members)	AGACTGAGCACGUGANNNNNTTAAGCCAGCCCCGACAC	ACGTGCTCAGTCUGGGCCATTGCGTTGCGCTCACTG	Cloning of 5N library for CBE-PPE validation
ABE-PPA pseudo-7N oligo library (512 members)	AAAGNNNNNNNNNNNNNNNACGCAATGGCCCAGACTGAGCACGTGANNNNNNNTTAAGCCAGCCCCGAC	CCAGTCGGGAAACCTGTC	KLD cloning of ABE-PPE library, first set of Ns replaced by UMI tag(s), second set of Ns replaced by target PAMs
HTS-1 genomic site	ACACTCTTTCCCTACACGACGCTCTTCCGATCTNNNNATTGCTCTTTTCTCCGCCCA	TGGAGTTCAGACGTGTGCTCTTCCGATCTTTCACAAAACAGGGGTGGCT	HTS of genomic target site, see **Supplementary Table 5.**
HTS-2 genomic site	ACACTCTTTCCCTACACGACGCTCTTCCGATCTNNNNGCTCAGAAAAAGGGCCCTGA	TGGAGTTCAGACGTGTGCTCTTCCGATCTGAGATTCAGTGTGGTGGGGG	“”
HTS-3 genomic site	ACACTCTTTCCCTACACGACGCTCTTCCGATCTNNNNAACTTTCTATCCGTCCGCGT	TGGAGTTCAGACGTGTGCTCTTCCGATCTGGCTGTAGAGGGAGACAAGC	“”
HTS-4 genomic site	ACACTCTTTCCCTACACGACGCTCTTCCGATCTNNNNGACGTCTTCTCCTGTGGTGG	TGGAGTTCAGACGTGTGCTCTTCCGATCTGGGTGTCTGGCTGGAATCTC	“”
HTS-5 genomic site	ACACTCTTTCCCTACACGACGCTCTTCCGATCTNNNNGGGCCTGGAAGTTCGCTAAT	TGGAGTTCAGACGTGTGCTCTTCCGATCTTGGATCGCTTTTCCGAGCTT	“”
HTS-6 genomic site	ACACTCTTTCCCTACACGACGCTCTTCCGATCTNNNNGGCTCCCTCTCCCAGTTACCG	TGGAGTTCAGACGTGTGCTCTTCCGATCTCACCACCATCCGCTCTGCCC	“”
HTS-7 genomic site	ACACTCTTTCCCTACACGACGCTCTTCCGATCTNNNNGGGACTCAGTTCCAACCCAAATGC	TGGAGTTCAGACGTGTGCTCTTCCGATCTGGCATCCACAAATCACCTGGAGAG	“”
HTS-8 genomic site	ACACTCTTTCCCTACACGACGCTCTTCCGATCTNNNNGAACCCAGGTAGCCAGAGAC	TGGAGTTCAGACGTGTGCTCTTCCGATCTTCCTTTCAACCCGAACGGAG	“”
HTS-9 genomic site	ACACTCTTTCCCTACACGACGCTCTTCCGATCTNNNNCCTTTCCTCTGCCATCACGTGC	TGGAGTTCAGACGTGTGCTCTTCCGATCTCCTAGAAAGGCATGGATGAGAGAAGC	“”
HTS-10 genomic site	ACACTCTTTCCCTACACGACGCTCTTCCGATCTNNNNCCAAGTTTTGGGCTCCAGTATGGTC	TGGAGTTCAGACGTGTGCTCTTCCGATCTGCCACCTGGTTTATGGGATTTGTTACAG	“”
HTS-11 genomic site	ACACTCTTTCCCTACACGACGCTCTTCCGATCTNNNNGGCGAGGCAGAGGGTCCAAA	TGGAGTTCAGACGTGTGCTCTTCCGATCTCTCCTTCTGGGGCCTTTTTCCC	“”
HTS-12 genomic site	ACACTCTTTCCCTACACGACGCTCTTCCGATCTNNNNCCCCCGCACTCCTTCTTC	TGGAGTTCAGACGTGTGCTCTTCCGATCTAATCTACCTCCGCGGACCT	“”
HTS-13 genomic site	ACACTCTTTCCCTACACGACGCTCTTCCGATCTNNNNGCGCACCTCATGGAATCCCTTC	TGGAGTTCAGACGTGTGCTCTTCCGATCTCTTGCCTCCACTGGTTGTGCAG	“”
HTS-14 genomic site	ACACTCTTTCCCTACACGACGCTCTTCCGATCTNNNNGGCACCACTGTAGTTTAGTGATCCC	TGGAGTTCAGACGTGTGCTCTTCCGATCTACCCTTGACCCCCTCCACCAG	“”
HTS-15 genomic site	ACACTCTTTCCCTACACGACGCTCTTCCGATCTNNNNCATTCCCTCTTTAGCCAGAGCCGG	TGGAGTTCAGACGTGTGCTCTTCCGATCTCAGATCTATTGGAATCCTGGAGTGACC	“”
HTS-16 genomic site	ACACTCTTTCCCTACACGACGCTCTTCCGATCTNNNNCAGCAGCTGAACAAGGACAC	TGGAGTTCAGACGTGTGCTCTTCCGATCTAAGGAGGAACAGGAGAGCCA	“”
HTS-17 genomic site	ACACTCTTTCCCTACACGACGCTCTTCCGATCTNNNNCAGTGAAATCACCCTGGGGGATCA	TGGAGTTCAGACGTGTGCTCTTCCGATCTGAGGTGGGGGTTAAAGCGGAG	“”
HTS-18 genomic site	ACACTCTTTCCCTACACGACGCTCTTCCGATCTNNNNGCTGTTTTCCCCTCTGAGCTATCG	TGGAGTTCAGACGTGTGCTCTTCCGATCTCCAGCTCTGTGGGAAGCAACTG	“”

HTS-19 genomic site	ACACTCTTTCCCTACACGACGCTCTTCCGATCTNNNNGCTCCTCTCTGTTTGGCCTT	TGGAGTTCAGACGTGTGCTCTTCCGATCTCAGCGTCTGCAAAGAGGAGA	“”
HTS-20 genomic site	ACACTCTTTCCCTACACGACGCTCTTCCGATCTNNNNCCTCCTGAAAATGCACCCTCTTCT	TGGAGTTCAGACGTGTGCTCTTCCGATCTGGTGCATTTTTTAATAGGGCTTGGGG	“”
HTS-21 genomic site	ACACTCTTTCCCTACACGACGCTCTTCCGATCTNNNNGGACCTTATCTCCTTTCATTGAGCACC	TGGAGTTCAGACGTGTGCTCTTCCGATCTCATACTCGCATGGCTACCTGGAC	“”
HTS-22 genomic site	ACACTCTTTCCCTACACGACGCTCTTCCGATCTNNNNGGGGCCTCCTGAGTTTCTCATCTG	TGGAGTTCAGACGTGTGCTCTTCCGATCTGGTTGCCCACCCTAGTCATTGGAG	“”
HTS-23 genomic site	ACACTCTTTCCCTACACGACGCTCTTCCGATCTNNNNGGAATAGCACCAGAATGTTCGAGGC	TGGAGTTCAGACGTGTGCTCTTCCGATCTGCCTACACTTAAAAACTTGACGTGGG	“”
HTS-24 genomic site	ACACTCTTTCCCTACACGACGCTCTTCCGATCTNNNNGGAAAAGAGGTTGTGAGTGGTCCAG	TGGAGTTCAGACGTGTGCTCTTCCGATCTAGAATGCAGGGCTTGTGTACTTATAGC	“”
HTS-25 genomic site	ACACTCTTTCCCTACACGACGCTCTTCCGATCTNNNNGGTGGAAGGTCCCTCCAGA	TGGAGTTCAGACGTGTGCTCTTCCGATCTCCTTCAACCTGACCTGGGAC	“”
HTS-26 genomic site	ACACTCTTTCCCTACACGACGCTCTTCCGATCTNNNNGGTAGTGCTTGAGACCGCCAG	TGGAGTTCAGACGTGTGCTCTTCCGATCTCCCTCCACTAAGAAGAACCTCTTTGTG	“”
HTS-27 genomic site	ACACTCTTTCCCTACACGACGCTCTTCCGATCTNNNNCACACTCCCAGCTTCACCTC	TGGAGTTCAGACGTGTGCTCTTCCGATCTTCGTCCAACTCAGCCTTGTC	“”
HTS-28 genomic site	ACACTCTTTCCCTACACGACGCTCTTCCGATCTNNNNCGCTAGACGGTAGAGCCTACTGC	TGGAGTTCAGACGTGTGCTCTTCCGATCTCCCATTGCAACTTCCAGTCCTGC	“”
HTS-29 genomic site	ACACTCTTTCCCTACACGACGCTCTTCCGATCTNNNNGGAATAGCACCAGAATGTTCGAGGC	TGGAGTTCAGACGTGTGCTCTTCCGATCTGCCTACACTTAAAAACTTGACGTGGG	“”
HTS-30 genomic site	ACACTCTTTCCCTACACGACGCTCTTCCGATCTNNNNGCCACACCCTAGGGTTG	TGGAGTTCAGACGTGTGCTCTTCCGATCTGGGAAAATAGACCAATAGGCAG	“”
Site 1-OT1 genomic site	ACACTCTTTCCCTACACGACGCTCTTCCGATCTNNNNTCACAAGCTCGGTGGTTCAA	TGGAGTTCAGACGTGTGCTCTTCCGATCTAAAACCCTGTGTGCTCCTGA	HTS of genomic target site, see **Supplementary Table 2.**
Site 1-OT2 genomic site	ACACTCTTTCCCTACACGACGCTCTTCCGATCTNNNNCGCCCTCAGAGACCCTTTTT	TGGAGTTCAGACGTGTGCTCTTCCGATCTAGGGAGAGACTGGATGGTGG	“”
Site 1-OT3 genomic site	ACACTCTTTCCCTACACGACGCTCTTCCGATCTNNNNCTCAGCCACCCAACATGCTA	TGGAGTTCAGACGTGTGCTCTTCCGATCTCACAGTCCAGTTGAGGAGGC	“”
Site 1-OT4 genomic site	ACACTCTTTCCCTACACGACGCTCTTCCGATCTNNNNCCCAGCTCCACTCCACTTTC	TGGAGTTCAGACGTGTGCTCTTCCGATCTCCCCAGGCCTTAACATACCC	“”
Site 1-OT5 genomic site	ACACTCTTTCCCTACACGACGCTCTTCCGATCTNNNNGCCCAGAGCTGAGTAATGCT	TGGAGTTCAGACGTGTGCTCTTCCGATCTGCCTCTGTCTGTGTACTCCC	“”
Site 1-OT6 genomic site	ACACTCTTTCCCTACACGACGCTCTTCCGATCTNNNNCTTAAGGTGATCCTCCCGCT	TGGAGTTCAGACGTGTGCTCTTCCGATCTCCACACAAGAAGAGGAGGGG	“”
Site 1-OT7 genomic site	ACACTCTTTCCCTACACGACGCTCTTCCGATCTNNNNTGGGGCTTCCTCTCCAGG	TGGAGTTCAGACGTGTGCTCTTCCGATCTGGTGCTGGAGGGCTTGTG	“”
Site 2-OT1 genomic site	ACACTCTTTCCCTACACGACGCTCTTCCGATCTNNNNGCTGAAGCTGAGGCAGTAGG	TGGAGTTCAGACGTGTGCTCTTCCGATCTCTAGGAGGGGATGCAGACCT	“”
Site 2-OT2 genomic site	ACACTCTTTCCCTACACGACGCTCTTCCGATCTNNNNTGGGACTTGAATGTGTTCACAT	TGGAGTTCAGACGTGTGCTCTTCCGATCTAGACCTTCAAACGGTAAAGTCCA	“”
Site 2-OT3 genomic site	ACACTCTTTCCCTACACGACGCTCTTCCGATCTNNNNCAGCTTCCGAGGTCAAGAGG	TGGAGTTCAGACGTGTGCTCTTCCGATCTTGCTGAAACCCAGTGAAGCA	“”
Site 2-OT4 genomic site	ACACTCTTTCCCTACACGACGCTCTTCCGATCTNNNNCCTTTGGCCTTCCTGATAGCA	TGGAGTTCAGACGTGTGCTCTTCCGATCTTGGGTGACAAAAGGGGACTC	“”
Site 2-OT5 genomic site	ACACTCTTTCCCTACACGACGCTCTTCCGATCTNNNNGCCCTCCACTAAGAGCAGAG	TGGAGTTCAGACGTGTGCTCTTCCGATCTTGATTGTGTCTCGTCCCACG	“”

Site 2-OT6 genomic site	ACACTCTTTCCCTACACGACGCTCTTCCGATCTNNNNGTGAAGGCACCAGTACTCGT	TGGAGTTCAGACGTGTGCTCTTCCGATCTTGCTTCTCAGTCAGTGGCTG	“”
Site 2-OT7 genomic site	ACACTCTTTCCCTACACGACGCTCTTCCGATCTNNNNCAAGTGATCCTGCCACCTCA	TGGAGTTCAGACGTGTGCTCTTCCGATCTAACTTAACCAAGAAGGCCAGGT	“”
Site 2-OT8 genomic site	ACACTCTTTCCCTACACGACGCTCTTCCGATCTNNNNACCTCCTCCCTGGGATGAAA	TGGAGTTCAGACGTGTGCTCTTCCGATCTCGTCAACCGTGGGAATGTTT	“”
Site 2-OT9 genomic site	ACACTCTTTCCCTACACGACGCTCTTCCGATCTNNNNTTCCTACCTCTGCCACTTGC	TGGAGTTCAGACGTGTGCTCTTCCGATCTCCCCCTTCCTCTCTATGCCA	“”
Site 2-OT10 genomic site	ACACTCTTTCCCTACACGACGCTCTTCCGATCTNNNNTCCTGCTCCATTACAGCTGC	TGGAGTTCAGACGTGTGCTCTTCCGATCTTGGGTGCAGGTCTGAATCAC	“”
Site 2-OT11 genomic site	ACACTCTTTCCCTACACGACGCTCTTCCGATCTNNNNAGAGTGACTGTGGAGGGGAG	TGGAGTTCAGACGTGTGCTCTTCCGATCTTGACAGTACCCGTGATATGGTC	“”
Site 2-OT12 genomic site	ACACTCTTTCCCTACACGACGCTCTTCCGATCTNNNNCCTGATGCCTGGGAAGTGAA	TGGAGTTCAGACGTGTGCTCTTCCGATCTGCATTTAGCCCTTCACCTCCT	“”
Transcript for *in vitro transcription*	TCGAGCTCGGTACCTAATACGACTCACTATAAGGAAATAAGAGAGAAAAGAAG	TTTTTTTTTTTTTTTTTTTTTTTTTTTTTTTTTTTTTTTTTTTTTTTTTTTTTTTTTTTTTTTTTTTTTTTTTTTTTTTTTTTTTTTTTTTTTTTTTTTTTTTTTTTTTTTTTTTTTTTTTTCTTCCTACTCAGGCTTTATTCAAAGACCA	Generate linear PCR product for IVT
NR-Site3-OT1 genomic site	ACACTCTTTCCCTACACGACGCTCTTCCGATCTNNNNCGAAATAGGCCCTCTGTCCC	TGGAGTTCAGACGTGTGCTCTTCCGATCTGCTTCCTGGAAGATGCCATG	
NR-Site3-OT2 genomic site	ACACTCTTTCCCTACACGACGCTCTTCCGATCTNNNNCACTCTGATAATGGGGGAGGC	TGGAGTTCAGACGTGTGCTCTTCCGATCTTCAGCTGCCCTAAGATGTCTG	
NR-Site3-OT3 genomic site	ACACTCTTTCCCTACACGACGCTCTTCCGATCTNNNNCTGACCCCTGACTTGTTCCC	TGGAGTTCAGACGTGTGCTCTTCCGATCTAAGAGATGCTTGGGCTGTGG	Also used for C-Site3-OT3, C-Site3-OT4, and S-Site-OT1
C-Site3-OT1 genomic site	ACACTCTTTCCCTACACGACGCTCTTCCGATCTNNNNTTCCCCATGGTAGCCTGAGA	TGGAGTTCAGACGTGTGCTCTTCCGATCTTTCACGTATCCATCCGTCTATTCA	
C-Site3-OT2 genomic site	ACACTCTTTCCCTACACGACGCTCTTCCGATCTNNNNTTCACAAAGCCAGGTGCAGT	TGGAGTTCAGACGTGTGCTCTTCCGATCTAGGCAGCCTGATTCCCAATG	
S-Site3-OT2 genomic site	ACACTCTTTCCCTACACGACGCTCTTCCGATCTNNNNCTCTGTGTCTCCAGTGCCTG	TGGAGTTCAGACGTGTGCTCTTCCGATCTCCTCCCTGAACACTGGTGAC	
S-Site3-OT3 genomic site	ACACTCTTTCCCTACACGACGCTCTTCCGATCTNNNNTTGCTGTGTGTGCATGCAC	TGGAGTTCAGACGTGTGCTCTTCCGATCTGTGAAAAGCTGGGGAGGGAA	Also used for HF-Site3-OT1
S-Site3-OT4 genomic site	ACACTCTTTCCCTACACGACGCTCTTCCGATCTNNNNAGGTTGGCTTTAGATCCCTGA	TGGAGTTCAGACGTGTGCTCTTCCGATCTTAACTCCCAAATGCACTGCCT	
S-Site3-OT5 genomic site	ACACTCTTTCCCTACACGACGCTCTTCCGATCTNNNNATCCCTGCTCCTGCTCATCT	TGGAGTTCAGACGTGTGCTCTTCCGATCTTGCCCGTAGTAATCCCAGCT	
HF-Site3-OT2 genomic site	ACACTCTTTCCCTACACGACGCTCTTCCGATCTNNNNAATGGCCAAGACCGCAAGAA	TGGAGTTCAGACGTGTGCTCTTCCGATCTGGTGCTCTGTCATCCCCATC	
HF-Site3-OT3 genomic site	ACACTCTTTCCCTACACGACGCTCTTCCGATCTNNNNCCACACCTGATGGCTCACTC	TGGAGTTCAGACGTGTGCTCTTCCGATCTGGCTCATTGCATCCTTGACC	
HF-Site3-OT4 genomic site	ACACTCTTTCCCTACACGACGCTCTTCCGATCTNNNNCCTTGGCTTGTGGACCAGTA	TGGAGTTCAGACGTGTGCTCTTCCGATCT GGTAGGCTGAATAATGGCCCC	
HF-Site3-OT5 genomic site	ACACTCTTTCCCTACACGACGCTCTTCCGATCTNNNNCGACATCCTGGGTCATTGCT	TGGAGTTCAGACGTGTGCTCTTCCGATCT CTCTCTGACTGGGCTGCTTT	
NR-Site4-OT1 genomic site	ACACTCTTTCCCTACACGACGCTCTTCCGATCTNNNNGGGTGGATCATGAGGTCAGG	TGGAGTTCAGACGTGTGCTCTTCCGATCTGATGCTCAGTCTGTCACCCA	

NR-Site4-OT2 genomic site	ACACTCTTTCCCTACACGACGCTCTTCCGATCTNNNNAAGCACAGAGATAAAAGGACAGA	TGGAGTTCAGACGTGTGCTCTTCCGATCTTCCCTCTGGCTTCTTTTAAGTTTT	
NR-Site4-OT3 genomic site	ACACTCTTTCCCTACACGACGCTCTTCCGATCTNNNNAGCACAGAGATAAAAGGACAGAA	TGGAGTTCAGACGTGTGCTCTTCCGATCTTCCCTCTGGCTTCTTTTAAGTTT	
NR-Site4-OT4 genomic site	ACACTCTTTCCCTACACGACGCTCTTCCGATCTNNNNCCGGAGGTCTCTACTTCCCA	TGGAGTTCAGACGTGTGCTCTTCCGATCTAGGGCCGCCATACCATATTG	Also used for C-Site4-OT2 and C-Site4-OT4
C-Site4-OT1 genomic site	ACACTCTTTCCCTACACGACGCTCTTCCGATCTNNNNCAACAAATGATGCTGGCCGC	TGGAGTTCAGACGTGTGCTCTTCCGATCTTTGTTTGGAGAGGGGAGTGC	
C-Site4-OT3 genomic site	ACACTCTTTCCCTACACGACGCTCTTCCGATCTNNNNTCCAATATGGTATGGCGGCC	TGGAGTTCAGACGTGTGCTCTTCCGATCTGAGGATCCCACGTTAGTGCC	
S-Site4-OT1 genomic site	ACACTCTTTCCCTACACGACGCTCTTCCGATCTNNNNTGCTCATGGGATAGTGCTGA	TGGAGTTCAGACGTGTGCTCTTCCGATCTTTTCTCTGCCAGCCCTAGGA	Also used for HF-Site4-OT1
S-Site4-OT2 genomic site	ACACTCTTTCCCTACACGACGCTCTTCCGATCTNNNNAGGCAGGAGAGTAACTGGTCT	TGGAGTTCAGACGTGTGCTCTTCCGATCTTCCCCTTACACAACACACCTG	
S-Site4-OT3 genomic site	ACACTCTTTCCCTACACGACGCTCTTCCGATCTNNNNCCGAGCCCTTTAGCGCTAAT	TGGAGTTCAGACGTGTGCTCTTCCGATCTTTTGCGGCCCAAATCTCCTT	
S-Site4-OT4 genomic site	ACACTCTTTCCCTACACGACGCTCTTCCGATCTNNNNTCTCTGGGATCCTCAGCCTG	TGGAGTTCAGACGTGTGCTCTTCCGATCTCCATGTTCTCGCCAAAGCTG	
NR-Site5-OT1 genomic site	ACACTCTTTCCCTACACGACGCTCTTCCGATCTNNNNCCACCCAGAACCATCTCCCT	TGGAGTTCAGACGTGTGCTCTTCCGATCTCACTGTGGCTGGGAATCCTT	
NR-Site5-OT2 genomic site	ACACTCTTTCCCTACACGACGCTCTTCCGATCTNNNNTGAGAACAAGCTCAGCCCAG	TGGAGTTCAGACGTGTGCTCTTCCGATCTACTCCGTAGCATGGCTGTTC	
C-Site5-OT1 genomic site	ACACTCTTTCCCTACACGACGCTCTTCCGATCTNNNNGGTGACTCCATTGCCAAGGA	TGGAGTTCAGACGTGTGCTCTTCCGATCTTACTCCTCAGGCACTCACCA	
C-Site5-OT2 genomic site	ACACTCTTTCCCTACACGACGCTCTTCCGATCTNNNNACCATGCTTGTGGACCACA	TGGAGTTCAGACGTGTGCTCTTCCGATCTCCCATGCCCATTCTGCCTAT	
C-Site5-OT3 genomic site	ACACTCTTTCCCTACACGACGCTCTTCCGATCTNNNNCCCCACAGGCACCTTTTGA	TGGAGTTCAGACGTGTGCTCTTCCGATCTGACAATTCGCTGTCCACGTG	
C-Site5-OT4 genomic site	ACACTCTTTCCCTACACGACGCTCTTCCGATCTNNNNACAGAATATTCCACTGTTGATGAAAA	TGGAGTTCAGACGTGTGCTCTTCCGATCTGACACTTTGCTTGCTTCTGTGT	
S-Site5-OT1 genomic site	ACACTCTTTCCCTACACGACGCTCTTCCGATCTNNNNGACCCAACCTGAGACCAGTG	TGGAGTTCAGACGTGTGCTCTTCCGATCTACAGAGGCTAGCCAGTACCT	
S-Site5-OT2 genomic site	ACACTCTTTCCCTACACGACGCTCTTCCGATCTNNNNGAGGCCTGTTGCAGAGTAGA	TGGAGTTCAGACGTGTGCTCTTCCGATCTTGTTTTGGGGAAGGAAGGCTT	
S-Site5-OT3 genomic site	ACACTCTTTCCCTACACGACGCTCTTCCGATCTNNNNCCAACTCCGAGCTCCTTCTC	TGGAGTTCAGACGTGTGCTCTTCCGATCTTGTAGGCTGACAATGGCTGG	
S-Site5-OT4 genomic site	ACACTCTTTCCCTACACGACGCTCTTCCGATCTNNNNGTGAAGCCAAGGTGAGCAGA	TGGAGTTCAGACGTGTGCTCTTCCGATCTCAGCCTCAGCAAGACCTCAC	
HF-Site5-OT1	ACACTCTTTCCCTACACGACGCTCTTCCGATCTNNNNACCTGCTCCACATGGTGAAT	TGGAGTTCAGACGTGTGCTCTTCCGATCTTGGATCTTGCCTCTTGAGCA	
HF-Site5-OT2	ACACTCTTTCCCTACACGACGCTCTTCCGATCTNNNNGGTCACTTCCTGTGTTTAATGGC	TGGAGTTCAGACGTGTGCTCTTCCGATCTATCGGAGCATGAGAGAAGGC	
NR-Site6-OT1	ACACTCTTTCCCTACACGACGCTCTTCCGATCTNNNNCTGTGAGGCTCAGTTTCTGA	TGGAGTTCAGACGTGTGCTCTTCCGATCTTGTGCATCTAGTCACCCTACAC	
NR-Site6-OT2	ACACTCTTTCCCTACACGACGCTCTTCCGATCTNNNNGCTGCAGACCTTTGGACTCA	TGGAGTTCAGACGTGTGCTCTTCCGATCTTGTCACTGGGGAAGGGTTTG	Also used for C-Site6-OT3
NR-Site6-OT3	ACACTCTTTCCCTACACGACGCTCTTCCGATCTNNNNGCACGATTGACAGGTCATGG	TGGAGTTCAGACGTGTGCTCTTCCGATCTGACTATAAGTGCCTGCCACCA	

C-Site6-OT1	ACACTCTTTCCCTACACGACGCTCTTCCGATCTNNNNCGCACACTCTGTCAAATGGC	TGGAGTTCAGACGTGTGCTCTTCCGATCTTGGGAGCCAGAGAAGGAAAG	
C-Site6-OT2	ACACTCTTTCCCTACACGACGCTCTTCCGATCTNNNNGGAGGTGCCAGGTTCCTTT	TGGAGTTCAGACGTGTGCTCTTCCGATCTACTAGGTGGAGTCCGGATGA	
C-Site6-OT4	ACACTCTTTCCCTACACGACGCTCTTCCGATCTNNNNGCATTCGCATGGTGCAGTAG	TGGAGTTCAGACGTGTGCTCTTCCGATCTTTTCCTTCTGCCACACCCAG	
S-Site6-OT1	ACACTCTTTCCCTACACGACGCTCTTCCGATCTNNNNAGCTCAAGCAGTAAGTCAGCA	TGGAGTTCAGACGTGTGCTCTTCCGATCTAGTTGACAGGGGATTGGCTT	
S-Site6-OT2	ACACTCTTTCCCTACACGACGCTCTTCCGATCTNNNNTACTAGCAGCAGGAGCCTGT	TGGAGTTCAGACGTGTGCTCTTCCGATCTCCAGCCAGATGACTGACACT	
S-Site6-OT3	ACACTCTTTCCCTACACGACGCTCTTCCGATCTNNNNCAGTAGCTGGGCATCCCTTG	TGGAGTTCAGACGTGTGCTCTTCCGATCTTGACCCTTTCTGGCTTCAGAC	
S-Site6-OT4	ACACTCTTTCCCTACACGACGCTCTTCCGATCTNNNNAAGCGATCCACTCACCTTGG	TGGAGTTCAGACGTGTGCTCTTCCGATCTTCGTGGCCTTCTTTCTTTCCA	
S-Site6-OT5	ACACTCTTTCCCTACACGACGCTCTTCCGATCTNNNNGGCAAAGGGATGGATTGGGA	TGGAGTTCAGACGTGTGCTCTTCCGATCTTAGCTTTGTGGCATCAGGTGA	
HF-Site6-OT1	ACACTCTTTCCCTACACGACGCTCTTCCGATCTNNNNCATCATTTGCATGCAGACTTGT	TGGAGTTCAGACGTGTGCTCTTCCGATCTTTGTTATGGTCAATTATCAGGATGGA	
HBB-NRCH-OT1 genomic site	ACACTCTTTCCCTACACGACGCTCTTCCGATCTNNNNTGGGCTTGTTTGGTACTTCC	TGGAGTTCAGACGTGTGCTCTTCCGATCTACCATTTGGCAAACACCACA	
HBB-NRCH-OT2 genomic site	ACACTCTTTCCCTACACGACGCTCTTCCGATCTNNNNAAACAGCCCAAGGGACAGAG	TGGAGTTCAGACGTGTGCTCTTCCGATCTAGCATAAAAGGCAGGGCAGA	Also used for HBB-Nme-OT1
HBB-NRCH-OT3 genomic site	ACACTCTTTCCCTACACGACGCTCTTCCGATCTNNNNCACTTAGGATGGAGGCACGG	TGGAGTTCAGACGTGTGCTCTTCCGATCTCATCTCTCATCCACCTGCCC	Also used for HBB-Nme-OT5
HBB-NRCH-OT4 genomic site	ACACTCTTTCCCTACACGACGCTCTTCCGATCTNNNNGGTAGGCAGGGGGTTCAATT	TGGAGTTCAGACGTGTGCTCTTCCGATCTAGCCAAGTGGTTGTGAGGAG	
HBB-NRCH-OT5 genomic site	ACACTCTTTCCCTACACGACGCTCTTCCGATCTNNNNCTGTTGCCACAGGGAAGTGA	TGGAGTTCAGACGTGTGCTCTTCCGATCTTGTTGGCACCTGTAGTCCCA	
HBB-NRCH-OT6 genomic site	ACACTCTTTCCCTACACGACGCTCTTCCGATCTNNNNAGCCACATCTTGCTCTTGCT	TGGAGTTCAGACGTGTGCTCTTCCGATCTCAGTCTCCTAAGGTGCTGGG	
HBB-NRCH-OT7 genomic site	ACACTCTTTCCCTACACGACGCTCTTCCGATCTNNNNGTCCCCACTCTGTTGGAAGG	TGGAGTTCAGACGTGTGCTCTTCCGATCTGAGCACCCCACTCCAGAAAA	
HBB-NRCH-OT8 genomic site	ACACTCTTTCCCTACACGACGCTCTTCCGATCTNNNNAGGGACTGAAGCCACCTTTT	TGGAGTTCAGACGTGTGCTCTTCCGATCTCAGGAGTCACGTGTCAAGGT	
HBB-NRCH-OT9 genomic site	ACACTCTTTCCCTACACGACGCTCTTCCGATCTNNNNTGCCTCCTTTGAGCCATTTGA	TGGAGTTCAGACGTGTGCTCTTCCGATCTGCATGGTGAGTTTAAGGGCC	
HBB-NRCH-OT10 genomic site	ACACTCTTTCCCTACACGACGCTCTTCCGATCTNNNNACAGGTATGAGCCACTGTGC	TGGAGTTCAGACGTGTGCTCTTCCGATCTGCAAGAAAGGGCATGGTGTG	Also used for HBB-Nme-OT8
HBB-NRCH-OT11 genomic site	ACACTCTTTCCCTACACGACGCTCTTCCGATCTNNNNAACCAGTAAGTCAGGCCTGC	TGGAGTTCAGACGTGTGCTCTTCCGATCTTTGGGCCACAAGGTCTTTCA	Also used for HBB-Nme-OT9
HBB-Nme-OT2 genomic site	ACACTCTTTCCCTACACGACGCTCTTCCGATCTNNNNTTGCAATGGAACCATCTTTCTGG	TGGAGTTCAGACGTGTGCTCTTCCGATCTTGGATGGTTCACATGGCACT	
HBB-Nme-OT3 genomic site	ACACTCTTTCCCTACACGACGCTCTTCCGATCTNNNNGGTTGTGAGGTAGGGTTGGG	TGGAGTTCAGACGTGTGCTCTTCCGATCTTGTTGTATGGGTGCTGCTGT	
HBB-Nme-OT4 genomic site	ACACTCTTTCCCTACACGACGCTCTTCCGATCTNNNNATCTGAGGTGCCTTGAGGAC	TGGAGTTCAGACGTGTGCTCTTCCGATCTGCTGAGCGGAGGGAGTAATT	
HBB-Nme-OT6 genomic site	ACACTCTTTCCCTACACGACGCTCTTCCGATCTNNNNATGTCTGTCTGGAAGGGAGC	TGGAGTTCAGACGTGTGCTCTTCCGATCTCACTGACCCTGAGCCTGTAC	

HBB-Nme-OT7 genomic site	ACACTCTTTCCCTACACGACGCTCTTCCGATCTNNNNACGCACTCCTCTTTCTCTCAC	TGGAGTTCAGACGTGTGCTCTTCCGATCTCAAACAAAAGGGAGCCAGGC	
Site 7-OT1 genomic site	ACACTCTTTCCCTACACGACGCTCTTCCGATCTNNNNCGCAGTGGAGGAGTTGGAAA	TGGAGTTCAGACGTGTGCTCTTCCGATCTGCCACCTGCGTGAAAATGTT	
Site 7-OT2 genomic site	ACACTCTTTCCCTACACGACGCTCTTCCGATCTNNNNCTGGGGATTGCCTGAGGAAG	TGGAGTTCAGACGTGTGCTCTTCCGATCTTCCAAGTCAGACTCACGGGA	
Site 7-OT3 genomic site	ACACTCTTTCCCTACACGACGCTCTTCCGATCTNNNNCTTGGCTTGGGTCTGAGTGT	TGGAGTTCAGACGTGTGCTCTTCCGATCTGGTCACCCGAGAATGGAGAC	
Site 7-OT4 genomic site	ACACTCTTTCCCTACACGACGCTCTTCCGATCTNNNNGGAGTTGAATCTGACCCCCG	TGGAGTTCAGACGTGTGCTCTTCCGATCTCTCCACTGTGGGCTTCTCAA	
Site 7-OT5 genomic site	ACACTCTTTCCCTACACGACGCTCTTCCGATCTNNNNTGAGGTTGTTCACTGGATCCA	TGGAGTTCAGACGTGTGCTCTTCCGATCTCAGATGAAGTCAGGGTGAGGC	
Site 7-OT6 genomic site	ACACTCTTTCCCTACACGACGCTCTTCCGATCTNNNNTCCTGCTCAGTTCCAGTTGC	TGGAGTTCAGACGTGTGCTCTTCCGATCTTGGCCTCACTTTGTCACACC	
Site 7-OT7 genomic site	ACACTCTTTCCCTACACGACGCTCTTCCGATCTNNNNAGCTGGAGCCTCATCTTTGG	TGGAGTTCAGACGTGTGCTCTTCCGATCTCTCTACCACATCCCAGCACC	
Site 7-OT8 genomic site	ACACTCTTTCCCTACACGACGCTCTTCCGATCTNNNNGGCTGGTAGCTTTTGTACTGGA	TGGAGTTCAGACGTGTGCTCTTCCGATCTCCATATCTGTCCACAAATGAGTCC	
Site 7-OT9 genomic site	ACACTCTTTCCCTACACGACGCTCTTCCGATCTNNNNCAAGGGGAAAAGGCCAGAGG	TGGAGTTCAGACGTGTGCTCTTCCGATCTTGACTTTGTGGCCAACTTGC	
Site 7-OT10 genomic site	ACACTCTTTCCCTACACGACGCTCTTCCGATCTNNNNTAGACAAAGGATGTTGGGGCC	TGGAGTTCAGACGTGTGCTCTTCCGATCTAGCAGGAGACAGACTCAGCA	
Site 7-OT11 genomic site	ACACTCTTTCCCTACACGACGCTCTTCCGATCTNNNNCCACGACTGGAGCTGAAGAA	TGGAGTTCAGACGTGTGCTCTTCCGATCTATTCAAGCCTGCTGGGGTG	
Site 7-OT12 genomic site	ACACTCTTTCCCTACACGACGCTCTTCCGATCTNNNNTTCTTGCCACTCTTGGGGAC	TGGAGTTCAGACGTGTGCTCTTCCGATCTGGCCCCAAAGAGTTCCTCTC	
Site 8-OT1 genomic site	ACACTCTTTCCCTACACGACGCTCTTCCGATCTNNNNCCTTCAAGGGACCCATGAGG	TGGAGTTCAGACGTGTGCTCTTCCGATCTAATCATGTCGATCCTGGGGC	
Site 8-OT2 genomic site	ACACTCTTTCCCTACACGACGCTCTTCCGATCTNNNNGCTGGAAGGGCCCATCTATT	TGGAGTTCAGACGTGTGCTCTTCCGATCTTGTAAGGCCAGTGTCCAAAGG	
Site 8-OT3 genomic site	ACACTCTTTCCCTACACGACGCTCTTCCGATCTNNNNGGGGGACTAAGCAGAGGGAT	TGGAGTTCAGACGTGTGCTCTTCCGATCTACAAAGCCCTTCCACCCAAA	
Site 8-OT4 genomic site	ACACTCTTTCCCTACACGACGCTCTTCCGATCTNNNNCTGGGAGAGTGTCCTGAGGA	TGGAGTTCAGACGTGTGCTCTTCCGATCTTTGCTGGAAAACTGCCCTCA	
Site 8-OT5 genomic site	ACACTCTTTCCCTACACGACGCTCTTCCGATCTNNNNTCCTGCACAGAAGATGCCAG	TGGAGTTCAGACGTGTGCTCTTCCGATCTGCTCTGTGAGTGCCTTGCTA	
Site 8-OT6 genomic site	ACACTCTTTCCCTACACGACGCTCTTCCGATCTNNNNGCATAGCTCAGGGGATGTGT	TGGAGTTCAGACGTGTGCTCTTCCGATCTGAAAGCCTCACACTGCTCCT	
Site 8-OT7 genomic site	ACACTCTTTCCCTACACGACGCTCTTCCGATCTNNNNAGAGGGAGGGTGTATGCCTT	TGGAGTTCAGACGTGTGCTCTTCCGATCTGCCAGGTCTCTGCAGTTTCA	
Site 8-OT8 genomic site	ACACTCTTTCCCTACACGACGCTCTTCCGATCTNNNNGTAGGGGCTGTAGGGTCTGT	TGGAGTTCAGACGTGTGCTCTTCCGATCTTGTTGTTACTGTGGACCCCT	
Site 8-OT9 genomic site	ACACTCTTTCCCTACACGACGCTCTTCCGATCTNNNNAGTGGTCTTACCTGAGGCCT	TGGAGTTCAGACGTGTGCTCTTCCGATCTACAGGATCCACCATGACACG	
Site 8-OT10 genomic site	ACACTCTTTCCCTACACGACGCTCTTCCGATCTNNNNTGTGGGAGAACGTCGAAGTC	TGGAGTTCAGACGTGTGCTCTTCCGATCTCACAAGGTGCAGAGTCCCTT	
Site 8-OT11 genomic site	ACACTCTTTCCCTACACGACGCTCTTCCGATCTNNNNTTGCACTTGCTAGCCATGGA	TGGAGTTCAGACGTGTGCTCTTCCGATCTTTCTGTGGATGTCTGTGGGC	

Site 8-OT12 genomic site	ACACTCTTTCCCTACACGACGCTCTTCCGATCTNNNNGGGTGGGGAGTTTCAGGAAG	TGGAGTTCAGACGTGTGCTCTTCCGATCTTCACCCCACACTGGTAGACT	
Site 8-OT13 genomic site	ACACTCTTTCCCTACACGACGCTCTTCCGATCTNNNNCTAGCTGTGTCTTGCCTGCT	TGGAGTTCAGACGTGTGCTCTTCCGATCTCTGCTGGTTCTTTTTCGCCC	
Site 8-OT14 genomic site	ACACTCTTTCCCTACACGACGCTCTTCCGATCTNNNNCACCCACTCCCATCAACACC	TGGAGTTCAGACGTGTGCTCTTCCGATCTACTCCGGCAGTGGTAGTACT	
Site 8-OT15 genomic site	ACACTCTTTCCCTACACGACGCTCTTCCGATCTNNNNACCAGCAGTGCAATCTAGACTT	TGGAGTTCAGACGTGTGCTCTTCCGATCTAACAAGCCTGTTTGAGGATGC	
Site 8-OT16 genomic site	ACACTCTTTCCCTACACGACGCTCTTCCGATCTNNNNCCACCACCTCCACATGGATC	TGGAGTTCAGACGTGTGCTCTTCCGATCTTGTGAAGCAGAGAGGTCAGC	
Site 8-OT17 genomic site	ACACTCTTTCCCTACACGACGCTCTTCCGATCTNNNNAGCTTTCCTAGGGAGGGAGG	TGGAGTTCAGACGTGTGCTCTTCCGATCTCCCCTTCTAACACCCCTTGC	
Site 8-OT18 genomic site	ACACTCTTTCCCTACACGACGCTCTTCCGATCTNNNNCTGTCCTGCCATTCCCCTAC	TGGAGTTCAGACGTGTGCTCTTCCGATCTTCACCCAACTCAGCGTTTGA	
Site 8-OT20 genomic site	ACACTCTTTCCCTACACGACGCTCTTCCGATCTNNNNTCCCCATTTCACACTCTCCC	TGGAGTTCAGACGTGTGCTCTTCCGATCTGCCTGGGTGACAAGAGCAA	
Site 8-OT21 genomic site	ACACTCTTTCCCTACACGACGCTCTTCCGATCTNNNNAGCCTCAGCCCATACAGAGA	TGGAGTTCAGACGTGTGCTCTTCCGATCTCCGATGGCCATCAGGATCAT	Also used for Site 8-OT22
Site 8-OT23 genomic site	ACACTCTTTCCCTACACGACGCTCTTCCGATCTNNNNGCAGTAAAGTGATGCCCCCT	TGGAGTTCAGACGTGTGCTCTTCCGATCTAAGTTTCGCTAGGGTGCAGT	
Site 8-OT25 genomic site	ACACTCTTTCCCTACACGACGCTCTTCCGATCTNNNNGAGGCTGTGTCCATGATGCT	TGGAGTTCAGACGTGTGCTCTTCCGATCTCTCTTTCAGCTGGGTCAGGG	
Site 8-OT27 genomic site	ACACTCTTTCCCTACACGACGCTCTTCCGATCTNNNNGATAGTGGTGCTGGCTGAGG	TGGAGTTCAGACGTGTGCTCTTCCGATCTTGGGTTCCCATTCACCAAGG	

Supplementary Table 9. Chemically-synthesized guide RNAs used for HDFa cells

Site/Guide ID*	Protospacer sequence (5'-3')	sgRNA scaffold (5'-3')
ACC-1/Nme1	CGCAAAGCTGCATCCACCCCCCG	GTTGTAGCTCCCTTTCTCATTTCGGAAACGAAATGAGAACCGTTGCTA CAATAAGGCCGTCTGAAAAGATGTGCCGCAACGCTCTGCCCCTTAAAG CTTCTGCTTTAAGGGGCATCGTTTATTTT^
GCA-1/Nme11	GGGTCCAAAGCAGGATGACAGGCA	GTTGTAGCTCCCTTTCTCATTTCGGAAACGAAATGAGAACCGTTGCTA CAATAAGGCCGTCTGAAAAGATGTGCCGCAACGCTCTGCCCCTTAAAG CTTCTGCTTTAAGGGGCATCGTTTATTTT^
TCA-1/Nme14	TGGCTACAGCAACAGGGTGGTGG	GTTGTAGCTCCCTTTCTCATTTCGGAAACGAAATGAGAACCGTTGCTA CAATAAGGCCGTCTGAAAAGATGTGCCGCAACGCTCTGCCCCTTAAAG CTTCTGCTTTAAGGGGCATCGTTTATTTT^
CCG-1/Nme18	GTCTCCGCTTTAACCCCCACCTC	GTTGTAGCTCCCTTTCTCATTTCGGAAACGAAATGAGAACCGTTGCTA CAATAAGGCCGTCTGAAAAGATGTGCCGCAACGCTCTGCCCCTTAAAG CTTCTGCTTTAAGGGGCATCGTTTATTTT^
GCG-1/Nme22	GGTGTGCAGACGGCAGTCACTAG	GTTGTAGCTCCCTTTCTCATTTCGGAAACGAAATGAGAACCGTTGCTA CAATAAGGCCGTCTGAAAAGATGTGCCGCAACGCTCTGCCCCTTAAAG CTTCTGCTTTAAGGGGCATCGTTTATTTT^
GCT-1/Nme29	GCACAACCAGTGGAGGCAAGAGG	GTTGTAGCTCCCTTTCTCATTTCGGAAACGAAATGAGAACCGTTGCTA CAATAAGGCCGTCTGAAAAGATGTGCCGCAACGCTCTGCCCCTTAAAG CTTCTGCTTTAAGGGGCATCGTTTATTTT^
GCT-2/Nme30	GAAATGGCACACACATCCCTCGT	GTTGTAGCTCCCTTTCTCATTTCGGAAACGAAATGAGAACCGTTGCTA CAATAAGGCCGTCTGAAAAGATGTGCCGCAACGCTCTGCCCCTTAAAG CTTCTGCTTTAAGGGGCATCGTTTATTTT^
ACC-1/SpRY1	GCTGCATCCACCCCCCGAGG	SpCas9 sgRNA EZ Kit scaffold from Synthego (with 2'-O-Methyl and 3' phosphorothioate bond modifications)
GCA-1/SpRY4	AAGCAGGATGACAGGCAGGG	SpCas9 sgRNA EZ Kit scaffold from Synthego (with 2'-O-Methyl and 3' phosphorothioate bond modifications)
TCA-1/SpRY7	CAGCAACAGGGTGGTGGACC	SpCas9 sgRNA EZ Kit scaffold from Synthego (with 2'-O-Methyl and 3' phosphorothioate bond modifications)
CCG-1/SpRY9	GCTTTAACCCCCACCTCCAG	SpCas9 sgRNA EZ Kit scaffold from Synthego (with 2'-O-Methyl and 3' phosphorothioate bond modifications)
GCG-1/SpRY10	CAGACGGCAGTCACTAGGGG	SpCas9 sgRNA EZ Kit scaffold from Synthego (with 2'-O-Methyl and 3' phosphorothioate bond modifications)
GCT-1/SpRY12	CCAGTGGAGGCAAGAGGGCG	SpCas9 sgRNA EZ Kit scaffold from Synthego (with 2'-O-Methyl and 3' phosphorothioate bond modifications)
GCT-2/SpRY13	GCACACACATCCCTCGTGCA	SpCas9 sgRNA EZ Kit scaffold from Synthego (with 2'-O-Methyl and 3' phosphorothioate bond modifications)

*See Supplementary Table 5 for list of target sites

^Nme2Cas9 chemically-synthesized sgRNAs ordered from IDT, including 2'-O-Methyl and 3' phosphorothioate bond modifications, as previously described[10]

Supplementary Note 1. ePACE pressure regulation

As IPP devices are sensitive to changes in pressure at valves and in connected media bottles, we developed an 8-channel pressure regulator that can be used to regulate these pressures through the eVOLVER framework. The device consists of sets of two proportional valves that can limit air flow from a high-pressure source and a vent at atmospheric pressure. By connecting an electronic pressure gauge to the output of this valve configuration, it is possible to implement proportional-integral-derivative (PID) control over the valves in order to set the output pressure to any desired level between the input and atmospheric pressure. We validated the functionality of this device by regulating pressure at 1.5 psi over 24 hours, and compared the performance of our device with that of a fixed, manually set regulator (PARKER-WATTS R25-02A) connected to the benchtop air supply (Supplementary Figure 3). The average pressure with PID control was 1.498 psi with an RMS error of 0.0086 psi, while the fixed regulator had an average pressure of 1.706 psi with an RMS error of 0.2220 psi. Large pressure deviations (>0.5 psi) that can affect the performance of the devices were observed with the fixed regulator, but were successfully eliminated with our automated pressure regulator scheme. We further characterized the effects of pressure changes at various locations in the system in order to optimize performance of the IPP devices for the course of a PACE experiment (Supplementary Figure 3).

Supplementary Note 2. ePACE2 recombination and cheating

During ePACE2, evolving Nme2Cas9 variants on the SP appeared to propagate well in all lagoons on targeted PAMs (each of the eight N_3YTN PAMs). Phage were sampled from some lagoons, and the insert was amplified via PCR. Following agarose gel electrophoresis, we found that these SP pools appeared to lose the expected Nme2Cas9 insert, as the resulting bands no longer corresponded to the correct insert size (**Supplementary Fig. 8a**). Sanger sequencing of the incorrectly sized band revealed a region of nucleotide homology between the N-terminus of the gIII construct on the AP and gVI in the phage genome (**Supplementary Fig. 8b,c**). This site of homology was likely acting as a recombination site enabling some phage to incorporate the gIII-C half into the SP genome. As gIII-N is constitutively expressed in the original SAC-PACE selection, this enables phage to propagate in a selection genomic siteree manner. For subsequent evolutions, we altered the codon usage of the N-terminus of gIII within the AP, such that the nucleotide homology was no

longer present (pTPH412, **Supplementary Table 7**). Following this alteration, recombination was no longer observed.

Supplementary Note 3. Validation of the split base editor SAC-PACE selection

To enable control over the expression of active enzyme in the SAC-PACE selection, we split Nme2ABE8e at the linker sequence between TadABE8e and Nme2Cas9. The TadABE8e-half was linked to the N-terminal half of the gp41-8 intein (gp41-8N), and this entire construct (TadABE8e-gp41-8N) was placed on a complementary plasmid (CP) under the control of a psp-promoter and a user-defined ribosome binding sequence. The C-terminal half of the base editor (dNme2Cas9) was linked to the C-terminal half of the gp41-8 intein (gp41-8C), and this construct (gp41-8C-dNme2Cas9) was recloned into the SP architecture (SP404, **Supplementary Table 7**). The split SAC-PACE selection was then validated by overnight propagation using new split-SP and host cells containing both AP and CP.

While testing the split SAC-PACE selection, we wanted to select a TadA variant with the highest Cas-*dependent* activity to limit bottlenecking the selection at the deamination step. In addition to TadABE8e, we tested the TadABE8e-R26G point mutant that had converged in prior evolutions (**Supplementary Fig. 6a, 10a**). TadABE8e-R26G enabled 10- to 20 genomic siteold stronger propagation compared to wild-type TadABE8e in a Cas-dependent manner, with no propagation in host-cells lacking Nme2Cas9. Moving forward, we chose to use TadABE8e-R26G in split base editor SAC-PACE selections (split SAC-PACE).

Supplementary Note 4. PAM-specific activity of ePACE4 evolved variants observed in ABE-PPA.

Activity improvements of ePACE4 variants on specific PAMs appeared to be agnostic of the PAM targeted during evolution, with most variants preferring N_4<u>CA</u> > N_4<u>CC</u> > N_4<u>CT</u> > N_4<u>CG</u>. The exceptions were variants evolved on the N_3<u>TCG</u> PAM, which exhibited N_4<u>CG</u> activity comparable to or better than activity on the other three N_4<u>CD</u> PAMs. This result would suggest that binding of the position 6 G is distinct from binding to the other three nucleobases. In line with this hypothesis, the mutation profiles in the PID are relatively conserved between variants evolved on the N_3<u>TCA</u> and N_3<u>TCT</u> APs (S933R, R1033S/G, Q1047R); however, additional mutations outside of the three seen in those variants converged in the N_3<u>TCG</u> trajectory (D873V, E932K, D961G, N1031D/S, K1044R, E1045A, K1077E) (**Extended Data Fig. 3c**). We note, however, that the difference in activity between

the variants was nuanced, as the overall trend reflects general improvements to activity on all N_4CN PAMs (**Extended Data Fig. 3b**).

Supplementary Note 5. Reversion analysis of eNme2-C RuvC/HNH domain mutations.

Simple reversion of the RuvC-inactivating mutation D16A in eNme2-C did not yield a robust nuclease Cas9. Upon reversion of the eight mutations in the RuvC/HNH domains and their associated linker regions to their wild-type residues, the resulting variant eNme2-C.NR had robust nuclease activity across N_4CN PAM sites. However, reversion of these mutations had a detrimental effect on base editing activity, as the ABE8e version of eNme2-C.NR had a 1.8 genomic siteold reduction in adenine base editing activity relative to eNme2-C-ABE8e (**Extended Data Fig. 7e**). These results would suggest that some or all the mutations in the RuvC/HNH domains are important for Nme2Cas9 activities necessary for base editing, but detrimental to the subsequent activation or catalytic activity of Nme2Cas9 nuclease.

To further explore this idea and to potentially find an optimal dual base editor/nuclease variant, we generated the set of eight single-point reversion variants of mutations in the RuvC/HNH domains of eNme2-C and evaluated them as nucleases and ABEs (**Extended Data Fig. 7e,f**). Only two of the eight single-point reversion variants, eNme2-C V696F and eNme2-C R711G, showed significant rescue of nuclease activity (12.5- and 4.4 genomic siteold improvement over eNme2-C, respectively). Conversely, most of the reversions reduced ABE efficiency relative to eNme2-C-ABE8e. Notably, none of the eight variants outperformed eNme2-C as an ABE or eNme2-C.NR as a nuclease, highlighting the importance of the amino acid identities at these RuvC/HNH positions in differentiating between base editor and nuclease activities of evolved Nme2Cas9.

Supplementary Note 6. Analysis and limitations of BE-PPA for evaluating Nme2Cas9 PAM compatibility.

All Nme2Cas9 variants (wild-type and evolved) were profiled using ABE-PPA on a single protospacer ("ABE-PPA", see **Supplementary Table 5**) flanked by 512 unique PAMs (pTPH424, see **Supplementary Tables 1 and 7**). The 512 unique PAMs are only a subset of the theoretical PAM space potentially encompassed by Nme2Cas9 (a six base pair region encompasses 4,096 targetable sequences). We designed the library to observe PAM compatibilities primarily at PAM positions 4-7 (NNN<u>NNNN</u>, 256 combinations), as we hypothesized that the positions that would most likely alter their nucleotide preference during

evolution are the positions canonically recognized by the wild-type enzyme (PAM positions 5 and 6, ± 1 base). We also included two groups of sequences at PAM positions 1-3 (ACGNNNN or CATNNNN), giving the total 512 PAM sequences, although these positions were pooled during analysis. We limited the library size to 512 members for throughput-purposes, as this number allows for profiling of up to 8 variants on an Illumina MiSeq kit (15 m reads, 1.9 m reads per variant, ~4,000 reads per PAM assuming equal distribution). We note that by limiting the analysis to these positions, it is possible that the PAM compatibility observed is biased by the identity of the bases chosen for positions 1-3, the selected protospacer, or the target adenine position. A larger library may be useful for more comprehensive PAM profiling of final variants. Nevertheless, the subset library used in this work provided a rapid, high-throughput method for quickly filtering evolved variants with desired PAM compatibilities and high efficiencies.

To analyze BE-PPA sequenced files, the demultiplexed fastq files were filtered using the seqkit package/grep function[11] to search for two flank sequences near either end of the amplicon. For ABE-PPA profiled variants, groups of PAMs were UMI-tagged, and the specific UMI tag was used in place of one of the flank sequences. Filtered files were then binned into individual fastq files per PAM using the same function. The resulting PAM-specific fastq files were analyzed using standard CRISPResso2[12] analysis.

Supplementary Note 7. Design error for the N_4CN trajectory dual PAM split SAC-PACE APs.

When designing the dual PAM split SAC-PACE APs (pTPH418b, see **Supplementary Table 5**), the identity of PAM positions 1-3 were set as CTT and AGG for the two target PAMs, both of which fall on the non-coding strand. The TT nucleotides of the CTT-containing PAM occupy codon positions two and three for an arbitrary codon within the AP linker. Notably, when the target PAM is designed to be 3'-CTTACN-5', the 3'-TTA-5' nucleotides introduce an additional stop codon in the PAM (5'-TAA-3' on coding strand), preventing proper correction of the AP. As such, none of the dual PAM split SAC-PACE APs containing an A at PAM position 4 were able to support phage propagation, as observed.

Supplementary Note 8. Evolved Nme2Cas9 amino acid sequences.

eNme2-C:

MAAFKSNPINYILGLDIGIASVGWAMVEIDEEGNPIRLIDLGVRVFERAEVPKTGDSLAMARRL
ARSVRRLTRRRAHRLLRARRLLKREGVLQAADFDENGLITSLPNTPWQLRAAALDRKLTPLE
WSAVLLHLIKHRGYLSQRKNEGETAAKELGALLKGVANNAHALQTGDFRTPAELALNKFEKE
SGHIRNQRGDYSHTFSRKDLQAELILLFEKQKEFGNPHVSGGLKEGIETLLMTQRPALSGDA
VQKMLGHCTLEPTEPKAAKNTYTAERFIWLTKLNNLRILEQGSERPLTDTERSTLMDEPYRK
SKLTYAQARKLLGLEDTAFFKGLRYGKDNAEASTLMEMKAYHAISRALEKEGLKDKKSPLNL
SSELQDEIGTAFSLFKTDEDITGRLKDRVQPEILEALLKHISFDKFVQISLKALRRIVPLMEQGK
RYDEACAEIYGVHYGKKNTEEKIYLPPIPADEIRNPVVLRALSQARKVINGVVRRYGSPARIHI
ETAREVGKSFKDRKEIAKRQEENRKDREKAAAKFREYFPNFVGEPKSKDILKLRLYEQQHGK
CLYSGKEINLVRLNEKGYVEIDHALPFSRTWDDSFNNKVLVLGSENQNKGNQTPYEYFNGK
DNSREWQEFKARVETSRFPSSKKQRILLQKFDEDGFKECNLNDTRYVNRFLCQFVADHILLT
GKGKRRVVASNGQITNLLRGFWRLRKVRAENDRHHALDAVVVACSTVAMQQKITRFVRYKE
MNAFDGKTVDKETGKVLYQKTHFPQPWEFFAQEVMIRVFGKPDGKPEFEEADTPEKLRTLL
AEKLSSRPEAVHEYVTPLFVSRAPNRKMSGAHKDTLRSAKRFVKHNEKISVKRVWLTEIKLA
DLENMVNYKNGREIELYEALKARLEAYGGNAKQAFDPKDNPFYKKGGQLVKAVRVEKTQKS
GVLLNKKNAYTIADNGDMVRVDVFCKVDKKGKNQYFIVPIYAWQVAENILPDIDCKGYRIDDS
YTFCFSLHKYDLIAFQKDEKSKVEFAYYINCDSSSGGFYLAWHDKGSREQRFRISTQNLALIQ
KYQVNELGKEIRPCRLKKRPPVR

eNme2-C.NR:

MAAFKPNPINYILGLDIGIASVGWAMVEIDEEENPIRLIDLGVRVFERAEVPKTGDSLAMARRL
ARSVRRLTRRRAHRLLRARRLLKREGVLQAADFDENGLITSLPNTPWQLRAAALDRKLTPLE
WSAVLLHLIKHRGYLSQRKNEGETAAKELGALLKGVANNAHALQTGDFRTPAELALNKFEKE
SGHIRNQRGDYSHTFSRKDLQAELILLFEKQKEFGNPHVSGGLKEGIETLLMTQRPALSGDA
VQKMLGHCTLEPTEPKAAKNTYTAERFIWLTKLNNLRILEQGSERPLTDTERSTLMDEPYRK
SKLTYAQARKLLGLEDTAFFKGLRYGKDNAEASTLMEMKAYHAISRALEKEGLKDKKSPLNL
SSELQDEIGTAFSLFKTDEDITGRLKDRVQPEILEALLKHISFDKFVQISLKALRRIVPLMEQGK
RYDEACAEIYGVHYGKKNTEEKIYLPPIPADEIRNPVVLRALSQARKVINGVVRRYGSPARIHI
ETAREVGKSFKDRKEIEKRQEENRKDREKAAAKFREYFPNFVGEPKSKDILKLRLYEQQHGK
CLYSGKEINLVRLNEKGYVEIDHALPFSRTWDDSFNNKVLVLGSENQNKGNQTPYEYFNGK
DNSREWQEFKARVETSRFPRSKKQRILLQKFDEDGFKECNLNDTRYVNRFLCQFVADHILLT
GKGKRRVFASNGQITNLLRGFWGLRKVRAENDRHHALDAVVVACSTVAMQQKITRFVRYKE
MNAFDGKTIDKETGKVLHQKTHFPQPWEFFAQEVMIRVFGKPDGKPEFEEADTPEKLRTLLA
EKLSSRPEAVHEYVTPLFVSRAPNRKMSGAHKDTLRSAKRFVKHNEKISVKRVWLTEIKLAD
LENMVNYKNGREIELYEALKARLEAYGGNAKQAFDPKDNPFYKKGGQLVKAVRVEKTQKSG
VLLNKKNAYTIADNGDMVRVDVFCKVDKKGKNQYFIVPIYAWQVAENILPDIDCKGYRIDDSY
TFCFSLHKYDLIAFQKDEKSKVEFAYYINCDSSSGGFYLAWHDKGSREQRFRISTQNLALIQK
YQVNELGKEIRPCRLKKRPPVR

eNme2-T.1:

MAAFKPNPINYILGLDIGIASVGWAMVEIDEEENPIRLIDLGVRVFKRAEVPKTGDSLAMARRL
ARSMRRLTRRRAHRLLRARRLLKREGVLQAADFDENGLIKSLPNTPWQLRAAALDRKLAPL
EWSAVLLHLIKHRGYLSQRKNEGETAGKKLGALLKGVANNAHALQTGDFRTPAELALNKFEK
ESGHIRNQRGDYSHTFSRKDLQAELILLFEKQKEFGNPHVSGGLKEGIETLLMTQRPALSGD

AVQKMLGHCTFEPAEPKAAKNTYTAERFIWLTKLNNLRILEQGSERPLTDTERATLMDEPYR
KSKLTYAQARKLLGLEDTAFFKGLRYGKDNAEASTLMEMKAYHAISRALEKEGLKDKKSPLN
LSSELQDEIGTAFSLFKTDEDIAGRLKDRVQPEILEALLKNISFDKFVQISLKSLRRIVPLMEQG
KRYDEACAEIYGDRYGKKNTEAKIYLPPIPADEIRNPVVLRALSQTRKVINGVVRRYGSPARIH
IETAREVGKSFKDRKEIEKRQEENRKDREKAAAKFREYFPNFVGEPKSKDILKLRLYEQQHG
KCLYSGKEINLVRLNEKGYVEIDHALPFSRTWDDSFNNKVLVLGSENQNKGNQTPYEYFNG
KDNPREWQEFKARVETSRFPRSKKQRILLQKFDEDGFKECNLNDTRYVSRFLCQFVADHILL
TGKGKRRVFASNGQITNLLRGFWGLRKVRAENARHHALDAVVVACSTVAMQQKITRFVRYK
EMNAFDGKTIDKETGKALYQKTRFPQPWEFFAQEVMIRVFGKPDGKPEFEEADTPEKLRTLL
AEKLSSRPEAAHEYVTPLFVSRAPNRKMSGAHKATLRSAKRFVKHNEKVSVKRVLLTEIKLA
DLENMVNYKNGREIELYEALKARLEAYGGNAKQAFDPKDNPFYKKGGQLVKAVRVEKTQES
GVLLNKKNAYTIADNGDRVRVDVFCKVDKKGKNQYFIVPIYAWQVAENILPDIDCKGYRIDDS
YTFCFSLHRYDLIAFQKDEKSKVEFAYYINCNASNGYFYLAWHDKGSKEQQFSISTQNLVLIQ
KYQVSELGKEIRPCRLKKRPPVR

eNme2-T.2:

MAAFKPNPINYILGLDIGIASVGWAMVEIDEEENPIRLIDLGVRVFKRAEVPKTGDSLAMARKL
ARSMRRLTRRRAHRLLRARRLLKREGVLQAADFDENGLIKSLPNTPWQLRATALDRKLAPLE
WSAVLLHLIKHRGYLSQRKNEGETANKKLGALLKGVANNAHALQTGDFRTPAELALNKFEKE
SGHIRNQRGDYSHTFSRKDLQAELILLFEKQKDFGNPHVSGGLKEGIETLLMTQRPALSGDA
VQKMLGHCTFEPAEPKAAKNTYTAERFIWLTKLNNLRILEQGSERPLTDTERATLMDEPYRK
SKLTYAQARKLLGLEDTAFFKGLRYGKDNAEASTLMEMKAYHAISRALEKEGLKDKKSPLNL
SSELQDEIGTAFSLFKTDEDIAGRLKDRVQPEILEALLKHISFDKFVQISLKALRRIVPLMEQGK
RYDEACAEIYGDRYGKKNTEKKIYLPPIPADEIRNPVVLRALSQARKVINGVVRRYGSPARIHI
ETAREVGKSFKDRKEIEKRQEENRKDREKAAAKFREYFPNFVGEPKSKDILKLRLYEQQHGK
CLYSGKEINLVRLNEKGYVEIDHALPFSRTWDDSFNNKVLVLGSENQNKGNQTPYEYFNGK
DNSREWQEFKARVETSRFPRSKKQRILLQKFDEDGFKECNLNDTRYVSRFLCQFVADHILLT
GKGKRRVFASNGQITNLLRGFWGLRKVRAENARHHSLDAVVVACSTVAMQQKITRFVRYKE
MNAFDGKTIDKETGKVLHQRTHFPQPWEFFAQEVMIRVFGKPDGKPEFEEADTPEKLRTLL
AEKLSIRPEAVHEYVTPLFVSRAPNRKMSGAHKATLRSAKRFVKHNEKISVKRVWLTEIKLAD
LENMVNYKNGREIELYEALKARLEAYGGNAKQAFDPKDNPFYKKGGQLVKAVRVEKTQKSG
VLLNKRNAYTIADNGDRVRVDVFCKVDKKGKNQYFIVPIYAWQVAENILPDIDCKGYRIDDSY
TFCFSLHRYDLIAFQKDEKSKVEFAYYINCNASNGNFYLAWHDKGSKEQQFCISTQNLVLIQK
YQVNELGKEIRPCRMKKRPPVR

Supplementary References

1 Wang, T., Badran, A. H., Huang, T. P. & Liu, D. R. Continuous directed evolution of proteins with improved soluble expression. *Nat Chem Biol* **14**, 972-980, doi:10.1038/s41589-018-0121-5 (2018).

2 Miller, S. M., Wang, T. & Liu, D. R. Phage-assisted continuous and non-continuous evolution. *Nature protocols* **15**, 4101-4127, doi:10.1038/s41596-020-00410-3 (2020).

3 Labun, K. *et al.* CHOPCHOP v3: expanding the CRISPR web toolbox beyond genome editing. *Nucleic Acids Res* **47**, W171-W174, doi:10.1093/nar/gkz365 (2019).

4 Newby, G. A. *et al.* Base editing of haematopoietic stem cells rescues sickle cell disease in mice. *Nature* **595**, 295-302, doi:10.1038/s41586-021-03609-w (2021).

5 Brissette, J. L., Weiner, L., Ripmaster, T. L. & Model, P. Characterization and sequence of the Escherichia coli stress-induced psp operon. *J Mol Biol* **220**, 35-48, doi:10.1016/0022-2836(91)90379-k (1991).

6 Ringquist, S. *et al.* Translation initiation in Escherichia coli: sequences within the ribosome-binding site. *Mol Microbiol* **6**, 1219-1229, doi:10.1111/j.1365-2958.1992.tb01561.x (1992).

7 Davis, J. H., Rubin, A. J. & Sauer, R. T. Design, construction and characterization of a set of insulated bacterial promoters. *Nucleic Acids Res* **39**, 1131-1141, doi:10.1093/nar/gkq810 (2011).

8 Gaudelli, N. M. *et al.* Directed evolution of adenine base editors with increased activity and therapeutic application. *Nature Biotechnology* **38**, 892-900, doi:10.1038/s41587-020-0491-6 (2020).

9 Richter, M. F., Zhao, K.T., Eton, E., Lapinaite, A., Newby, G.A., Thuronyi, B.W., Wilson, C., Zeng, J., Bauer, D.E., Doudna, J.A, Liu, D.R. Continuous evolution of an adenine base editor with enhanced Cas domain compatibility and activity. *Nature Biotechnology*, in press (2020).

10 Huang, T. P., Newby, G. A. & Liu, D. R. Precision genome editing using cytosine and adenine base editors in mammalian cells. *Nature Protocols* **16**, 1089-1128, doi:10.1038/s41596-020-00450-9 (2021).

11 Shen, W., Le, S., Li, Y. & Hu, F. SeqKit: A Cross-Platform and Ultrafast Toolkit for FASTA/Q File Manipulation. *PLOS ONE* **11**, e0163962, doi:10.1371/journal.pone.0163962 (2016).

12 Clement, K. *et al.* CRISPResso2 provides accurate and rapid genome editing sequence analysis. *Nature Biotechnology* **37**, 224-226, doi:10.1038/s41587-019-0032-3 (2019).
