# Supplementary material for: High-throughput continuous evolution of compact Cas9 variants targeting single-nucleotide-pyrimidine PAMs
Source: Nat Biotechnol. 2022 Sep 8;41(1):96–107. doi: 10.1038/s41587-022-01410-2 (PMC9849140; doi:10.1038/s41587-022-01410-2)
Supplement: Supplementary file 2 — Reporting Summary [file 41587_2022_1410_MOESM2_ESM.pdf]

nature research

Corresponding author(s): David R. Liu, Ahmad S. Khalil

Last updated by author(s): Jun 28, 2022

Reporting Summary

Nature Research wishes to improve the reproducibility of the work that we publish. This form provides structure for consistency and transparency in reporting. For further information on Nature Research policies, see our Editorial Policies and the Editorial Policy Checklist.

Statistics

For all statistical analyses, confirm that the following items are present in the figure legend, table legend, main text, or Methods section.

n/a	Confirmed	
☐	☒	The exact sample size (*n*) for each experimental group/condition, given as a discrete number and unit of measurement
☐	☒	A statement on whether measurements were taken from distinct samples or whether the same sample was measured repeatedly
☐	☒	The statistical test(s) used AND whether they are one- or two-sided<br>*Only common tests should be described solely by name; describe more complex techniques in the Methods section.*
☒	☐	A description of all covariates tested
☒	☐	A description of any assumptions or corrections, such as tests of normality and adjustment for multiple comparisons
☐	☒	A full description of the statistical parameters including central tendency (e.g. means) or other basic estimates (e.g. regression coefficient) AND variation (e.g. standard deviation) or associated estimates of uncertainty (e.g. confidence intervals)
☐	☒	For null hypothesis testing, the test statistic (e.g. *F*, *t*, *r*) with confidence intervals, effect sizes, degrees of freedom and *P* value noted<br>*Give P values as exact values whenever suitable.*
☒	☐	For Bayesian analysis, information on the choice of priors and Markov chain Monte Carlo settings
☒	☐	For hierarchical and complex designs, identification of the appropriate level for tests and full reporting of outcomes
☒	☐	Estimates of effect sizes (e.g. Cohen's *d*, Pearson's *r*), indicating how they were calculated

Our web collection on statistics for biologists contains articles on many of the points above.

Software and code

Policy information about availability of computer code

Data collection	MiSeq Control software (3.1) was used on the Illumina MiSeq sequencers to collect the high-throughput sequencing data. Default Bio-Rad CFX96 Touch Real-Time PCR Detection System software was used to collect qPCR data.
Data analysis	CRISPResso2 was used to analyze high-throughput sequencing files and quantify editing activity. The seqkit analysis package was used to sort and filter BE-PPA sequencing files. Aside from GUIDE-Seq plots, all statistical analysis and plotting were done using GraphPad Prism 7. All custom data analysis scripts as well as eVOLVER code used in this set of experiments are available on GitHub (https://github.com/khalillab/ePACE-Nme2Cas9-analysis).

For manuscripts utilizing custom algorithms or software that are central to the research but not yet described in published literature, software must be made available to editors and reviewers. We strongly encourage code deposition in a community repository (e.g. GitHub). See the Nature Research guidelines for submitting code & software for further information.

Data

Policy information about availability of data

All manuscripts must include a data availability statement. This statement should provide the following information, where applicable:
- Accession codes, unique identifiers, or web links for publicly available datasets
- A list of figures that have associated raw data
- A description of any restrictions on data availability

High-throughput DNA sequencing FASTQ files are available from the NCBI SRA under BioProject PRJNA853525. Other data are available from the corresponding authors upon reasonable request. Plasmids encoding select SAC-PACE components and evolved Nme2Cas9 genome editing agents have been deposited at Addgene for distribution.

Field-specific reporting

Please select the one below that is the best fit for your research. If you are not sure, read the appropriate sections before making your selection.

☒ Life sciences ☐ Behavioural & social sciences ☐ Ecological, evolutionary & environmental sciences

For a reference copy of the document with all sections, see nature.com/documents/nr-reporting-summary-flat.pdf

Life sciences study design

All studies must disclose on these points even when the disclosure is negative.

Sample size	Sample sizes were determined based on literature precedence for directed evolution (n = 2) and mammalian cell genome editing (n = 3). BE-PPA experiments (aside from validation), GUIDE-Seq, and mRNA dose titration into primary human fibroblasts were only performed with one replicate (per site for the mRNA experiment) and were limited this way due to the throughput and associated costs.
Data exclusions	No data was excluded from analysis.
Replication	With the exception of evolutions (where stochasticity is expected and replication is not standard in the field), GUIDE-Seq (n = 1 is standard in the field), ABE-PPA profiling (used primarily as an intermediate assay), and mRNA dose titration (only one replicate but two sites tested and averaged), all experiments were replicated at least once. Each measurement for IPP device calibration was done in triplicate, and three devices were used to characterize design performance over time. All attempts at replication were successful.
Randomization	Bacteria and/or mammalian cells used for these experiments were grown under identical conditions, so randomization was not used.
Blinding	Bacteria and/or mammalian cells used for these experiments were grown under identical conditions, so blinding was not used.

Reporting for specific materials, systems and methods

We require information from authors about some types of materials, experimental systems and methods used in many studies. Here, indicate whether each material, system or method listed is relevant to your study. If you are not sure if a list item applies to your research, read the appropriate section before selecting a response.

Materials & experimental systems

n/a	Involved in the study	
☒	☐	Antibodies
☐	☒	Eukaryotic cell lines
☒	☐	Palaeontology and archaeology
☒	☐	Animals and other organisms
☒	☐	Human research participants
☒	☐	Clinical data
☒	☐	Dual use research of concern

Methods

n/a	Involved in the study	
☒	☐	ChIP-seq
☒	☐	Flow cytometry
☒	☐	MRI-based neuroimaging

Eukaryotic cell lines

Policy information about cell lines

Cell line source(s)	HEK293T (ATCC), HEK293T with HBB mutation (generated previously), HDFa (ATCC), U2OS (ATCC), Huh7 (previous gift from Erik Sontheimer Lab at UMMS via ATCC).
Authentication	HEK293T, HDFa, U2OS and Huh7 cell lines were authenticated by the supplier using STR analysis (ATCC).
Mycoplasma contamination	All cell lines tested negative for mycoplasma.
Commonly misidentified lines (See ICLAC register)	None used.
